# Cardiovascular and mortality outcomes with JAK versus TNF inhibitors in rheumatoid arthritis: a target trial emulation (CARDINAL-RA)

**DOI:** 10.3389/fimmu.2026.1908442

**Published:** 2026-09-30

**Authors:** Rachael McClenahan, Laith Alamlih

**Affiliations:** 1 Department of Internal Medicine, Reading Hospital, West Reading, PA, United States; 2 Internal Medicine Department, Faculty of Medicine, Al-Quds University, Jerusalem, Palestine; 3 Rheumatology Department, Al-Mezan Specialty Hospital, Hebron, Palestine

**Keywords:** cardiovascular safety, electronic health records, Janus kinase inhibitors, major adverse cardiovascular events, real-world evidence, rheumatoid arthritis, target trial emulation, tumor necrosis factor inhibitors

## Abstract

**Objectives:**

This study aims to compare cardiovascular and mortality outcomes of Janus kinase inhibitors (JAKi) versus tumor necrosis factor inhibitors (TNFi) in rheumatoid arthritis (RA) at elevated cardiovascular risk using target trial emulation.

**Methods:**

Within TriNetX, adults with RA aged ≥50 years and ≥1 cardiovascular risk factor initiating JAKi or TNFi were compared in a new-user, active-comparator design. Improved 1:1 propensity score matching incorporated demographics, cardiovascular risk factors, comorbidities, individual conventional synthetic DMARDs, oral corticosteroids, prior non-TNF biologic exposure, and baseline inflammatory markers. The primary outcome was MACE (nonfatal myocardial infarction or nonfatal ischemic stroke); the secondary outcomes were all-cause mortality, heart failure hospitalization, and venous thromboembolism (VTE), with cataract as a prespecified negative control. Follow-up began 90 days after initiation. Cox models estimated hazard ratios, with 5-year Kaplan–Meier cumulative incidences and absolute risk differences per 1,000. Cohorts were separately matched by agent, sex, cardiovascular-risk stratum, and index era; E-values quantified sensitivity to unmeasured confounding.

**Results:**

In 18,757 matched pairs, JAKi was not associated with excess MACE (HR 1.002, 0.923–1.088; +2.11 per 1,000). All-cause mortality, a secondary outcome, was higher with JAKi (HR 1.158, 1.058–1.268; *p* = 0.0014; +10.4 per 1,000; E-value 1.586), whereas heart failure hospitalization (HR 1.019, 0.955–1.088) and VTE (HR 1.054, 0.961–1.156) did not differ; the negative control showed no excess (HR 0.934, 0.867–1.007). No agent showed a significant excess in MACE or mortality (mortality HR 1.052 tofacitinib, 1.213 baricitinib, and 1.090 upadacitinib); the only nominally significant agent-level finding was VTE with baricitinib (HR 1.364, 1.028–1.809; 198 events). A three-component composite including death was non-significant (HR 1.058, 0.992–1.127). No consistent effect modification was seen by sex, cardiovascular risk burden, or index year.

**Conclusion:**

JAKi initiation was not associated with excess MACE (primary outcome), heart failure hospitalization, or VTE. A modest excess in all-cause mortality was observed as a secondary outcome, with no signal for any individual agent. This association was of modest robustness (E-value 1.586) and was not reproduced in any agent-specific cohort, so residual channeling bias cannot be excluded. The null MACE result should not be read as class-wide cardiovascular safety reassurance nor the mortality signal as an established causal effect.

## Highlights

- The primary outcome, MACE, showed no excess with JAKi, nor did heart failure hospitalization or venous thromboembolism. A modest excess in all-cause mortality was seen as a secondary outcome, unadjusted for multiple comparisons.
- No individual agent carried a significant mortality signal; the only nominally significant agent-level finding was venous thromboembolism with baricitinib in a small matched cohort.
- These findings do not support the class-wide avoidance of JAK inhibitors, but neither do they establish differential safety between agents; continued mortality surveillance is warranted, recognizing the modest robustness of the mortality signal (E-value 1.586).

## Introduction

Rheumatoid arthritis (RA) affects approximately 1.3 million US adults and is a leading cause of disability globally (1). Beyond joint disease, its chronic systemic inflammation confers a 1.5- to 2.0-fold increased risk of cardiovascular mortality, with cardiovascular disease accounting for 40%–50% of excess mortality (2, 3). This reflects the interplay of inflammation, traditional risk factors, and therapy: tumor necrosis factor inhibitors (TNFi) and methotrexate may reduce cardiovascular risk, whereas corticosteroids and some NSAIDs increase it (2–4).

For inadequate response to conventional synthetic DMARDs (csDMARDs), guidelines recommend escalation to biologic or targeted synthetic DMARDs (5). TNFi have anchored advanced RA therapy for two decades, with robust efficacy and, in some studies, modest cardiovascular protection (4, 6). More recently, Janus kinase inhibitors (JAKi)—tofacitinib, baricitinib, and upadacitinib—have emerged as oral alternatives with efficacy comparable to TNFi in randomized trials (7, 8), but their real-world cardiovascular safety remains uncertain.

In 2021, the FDA issued a boxed warning for JAKi in RA patients aged ≥50 years with ≥1 cardiovascular risk factor, based on ORAL Surveillance, in which tofacitinib 10 mg twice daily showed numerically higher MACE and venous thromboembolism (VTE) than TNFi (9).

Despite its regulatory impact, ORAL Surveillance has limitations restricting generalizability. It evaluated only tofacitinib, including a dose no longer approved for RA, and enrolled a small, selected population with limited power for subgroup analyses by ASCVD status, age, and sex. Its median 4-year follow-up may miss longer-term risk, and the absence of absolute risk differences limits clinical interpretation (9, 10). *Post-hoc* analyses suggest excess MACE may concentrate in patients with established ASCVD, though estimates remain imprecise (10). Real-world comparisons are inconclusive: registry and claims analyses generally show no significant MACE difference (11–14) but are limited by short follow-up, misclassification, and low power (11–14), while baricitinib and upadacitinib data remain underpowered and inconsistently apply causal-inference methods (11, 15).

To address these gaps, we conducted a large real-world comparative effectiveness study using target trial emulation, a causal-inference framework approximating a randomized trial by aligning treatment initiation and follow-up to minimize immortal-time bias and using propensity scores to reduce confounding by indication (16, 17). Electronic health records enable long-term safety evaluation in large, diverse, routine-practice populations (18, 19). Within TriNetX, we compared 5-year cardiovascular and mortality outcomes among RA patients aged ≥50 years with ≥1 cardiovascular risk factor initiating JAKi versus TNFi, estimating absolute risk differences and assessing effect modification by sex, JAKi agent, baseline cardiovascular-risk burden in ASCVD-free patients, and index era (16).

## Methods

### Study design and data source

We conducted a retrospective comparative effectiveness study using a target trial emulation framework to reduce bias in observational data. The data came from TriNetX, a federated network of de-identified electronic health records from over 275 million patients across more than 50 US healthcare organizations. Analyses ran within the platform using standardized ontologies, with outputs limited to aggregate data, and followed STROBE and RECORD guidelines. Every cohort—main matched, three agent-specific, two sex strata, three ASCVD-free risk strata, two index-era strata, and six agent-by-era cells—was built as an independent network query rather than by subsetting one extraction (main cohort July 23, 2026; all stratified analyses July 25, 2026). Because contributing organizations upload continuously, each query returns the eligible population as of its run date, so pre-matching denominators differ between analyses: the eligible TNFi pool ranges from 55,266 (main cohort) to 57,077 (baricitinib and upadacitinib). **Supplementary Table S21** shows the list of pre-matching pool and extraction date for every cohort.

The denominators therefore do not sum as they would within a single extraction. Comparable stratified denominators consistently exceed the main-cohort figure and are never smaller: the TNFi pool is 55,266 in the main cohort against 56,175, 57,077 and 57,077 in the agent cohorts, 57,074 across the sex strata, and 55,766 across the index-era strata; the JAKi pool is 19,485 against 20,285 and 19,841 respectively. This is the expected signature of later extraction from a growing network, not double counting—within a single extraction, mutually exclusive strata sum to slightly less than the total because some patients have unrecorded sex (**Table 1**; 15,498 + 3,974 = 19,472 of 19,485 JAKi initiators). The agent-specific cohorts sum higher for a second reason: each indexes on first use of that agent, so patients switching between JAKi enter more than one cohort.

**Table 1:** Baseline characteristics before and after propensity score matching.

Characteristic	Before matching				After matching			
	JAKi (*n* = 19,485)	TNFi (*n* = 55,266)	*P*	SMD	JAKi (*n* = 18,757)	TNFi (*n* = 18,757)	*P*	SMD
Demographics								
Age, current, years, mean ± SD	67.3 ± 9.23	68.1 ± 9.56	<0.0001	0.0834	67.4 ± 9.23	67.2 ± 9.42	0.0100	0.0266
Age at index, years, mean ± SD	62.3 ± 9.14	61.5 ± 9.51	<0.0001	0.0767	62.3 ± 9.15	62.4 ± 9.44	0.2547	0.0118
Female	15,498 (79.538)	41,668 (75.395)	<0.0001	0.0993	14,862 (79.234)	14,898 (79.426)	0.6462	0.0047
Male	3,974 (20.395)	13,557 (24.530)	<0.0001	0.0992	3,883 (20.702)	3,842 (20.483)	0.6006	0.0054
Unknown gender	13 (0.067)	41 (0.074)	0.7386	0.0028	12 (0.064)	17 (0.091)	0.3530	0.0096
White	12,952 (66.472)	38,194 (69.109)	<0.0001	0.0565	12,478 (66.525)	12,484 (66.556)	0.9477	0.0007
Black or African American	2,692 (13.816)	8,251 (14.930)	0.0002	0.0318	2,608 (13.904)	2,574 (13.723)	0.6109	0.0053
Asian	839 (4.306)	1,639 (2.966)	<0.0001	0.0716	788 (4.201)	762 (4.062)	0.5000	0.0070
American Indian or Alaska Native	138 (0.708)	474 (0.858)	0.0465	0.0170	133 (0.709)	167 (0.890)	0.0487	0.0204
Native Hawaiian or Other Pacific Islander	52 (0.267)	158 (0.286)	0.6663	0.0036	51 (0.272)	51 (0.272)	1.0000	<0.0001
Other race	482 (2.474)	1,749 (3.165)	<0.0001	0.0418	468 (2.495)	476 (2.538)	0.7920	0.0027
Unknown race	2,330 (11.958)	4,801 (8.687)	<0.0001	0.1077	2,231 (11.894)	2,243 (11.958)	0.8484	0.0020
Hispanic or Latino	1,444 (7.411)	4,374 (7.914)	0.0241	0.0189	1,383 (7.373)	1,360 (7.251)	0.6483	0.0047
Not Hispanic or Latino	12,791 (65.645)	37,011 (66.969)	0.0008	0.0280	12,331 (65.741)	12,266 (65.394)	0.4800	0.0073
Unknown ethnicity	5,250 (26.944)	13,881 (25.117)	<0.0001	0.0416	5,043 (26.886)	5,131 (27.355)	0.3068	0.0106
Comorbidities (ICD-10-CM)								
Hypertensive diseases (I10–I1A)	12,765 (65.512)	35,714 (64.622)	0.0253	0.0187	12,295 (65.549)	12,320 (65.682)	0.7858	0.0028
Disorders of lipoprotein metabolism (E78)	10,932 (56.105)	28,423 (51.429)	<0.0001	0.0939	10,478 (55.862)	10,580 (56.406)	0.2886	0.0110
Overweight and obesity (E66)	5,890 (30.228)	15,211 (27.523)	<0.0001	0.0597	5,638 (30.058)	5,782 (30.826)	0.1062	0.0167
Type 2 diabetes mellitus (E11)	4,770 (24.480)	13,084 (23.675)	0.0233	0.0188	4,589 (24.466)	4,580 (24.418)	0.9139	0.0011
Ischemic heart diseases (I20–I25)	3,356 (17.224)	9,058 (16.390)	0.0072	0.0223	3,217 (17.151)	3,183 (16.970)	0.6407	0.0048
Nicotine dependence (F17)	3,304 (16.957)	10,027 (18.143)	0.0002	0.0312	3,192 (17.018)	3,217 (17.151)	0.7316	0.0035
Chronic kidney disease (N18)	1,839 (9.438)	4,308 (7.795)	<0.0001	0.0586	1,756 (9.362)	1,778 (9.479)	0.6974	0.0040
Heart failure (I50)	1,557 (7.991)	3,116 (5.638)	<0.0001	0.0935	1,471 (7.842)	1,507 (8.034)	0.4917	0.0071
Acute kidney failure (N17)	1,422 (7.298)	2,905 (5.256)	<0.0001	0.0842	1,343 (7.160)	1,360 (7.251)	0.7343	0.0035
Atrial fibrillation and flutter (I48)	1,271 (6.523)	3,192 (5.776)	0.0002	0.0311	1,225 (6.531)	1,236 (6.590)	0.8186	0.0024
Tobacco use (Z72.0)	1,166 (5.984)	3,325 (6.016)	0.8705	0.0014	1,120 (5.971)	1,128 (6.014)	0.8618	0.0018
Systemic lupus erythematosus (M32)	1,031 (5.291)	1,896 (3.431)	<0.0001	0.0912	955 (5.091)	917 (4.889)	0.3676	0.0093
Cerebral infarction (I63)	682 (3.500)	1,954 (3.536)	0.8173	0.0019	662 (3.529)	672 (3.583)	0.7804	0.0029
Systemic sclerosis (M34)	217 (1.114)	376 (0.680)	<0.0001	0.0460	197 (1.050)	204 (1.088)	0.7253	0.0036
Dermatopolymyositis (M33)	209 (1.073)	213 (0.385)	<0.0001	0.0808	168 (0.896)	151 (0.805)	0.3391	0.0099
Antiphospholipid syndrome (D68.61)	112 (0.575)	305 (0.552)	0.7118	0.0031	105 (0.560)	109 (0.581)	0.7839	0.0028
Psoriatic arthropathy (L40.5)	80 (0.411)	151 (0.273)	0.0030	0.0235	74 (0.395)	73 (0.389)	0.9341	0.0009
Ankylosing spondylitis (M45)	66 (0.339)	92 (0.166)	<0.0001	0.0343	57 (0.304)	52 (0.277)	0.6315	0.0050
Crohn’s disease (K50)	28 (0.144)	76 (0.138)	0.8422	0.0017	27 (0.144)	35 (0.187)	0.3092	0.0105
Ulcerative colitis (K51)	24 (0.123)	69 (0.125)	0.9544	0.0005	23 (0.123)	25 (0.133)	0.7727	0.0030
Medications (RxNorm)								
Prednisone	11,837 (60.749)	30,074 (54.417)	<0.0001	0.1284	11,242 (59.935)	11,409 (60.825)	0.0779	0.0182
Methotrexate	9,733 (49.951)	27,965 (50.601)	0.1190	0.0130	9,304 (49.603)	9,261 (49.374)	0.6570	0.0046
Methylprednisolone	8,583 (44.049)	18,461 (33.404)	<0.0001	0.2199	8,058 (42.960)	8,132 (43.354)	0.4405	0.0080
Hydroxychloroquine	6,951 (35.674)	16,764 (30.333)	<0.0001	0.1138	6,602 (35.198)	6,647 (35.437)	0.6269	0.0050
Dexamethasone	5,687 (29.187)	12,306 (22.267)	<0.0001	0.1588	5,367 (28.613)	5,487 (29.253)	0.1718	0.0141
Leflunomide	4,459 (22.884)	8,508 (15.395)	<0.0001	0.1913	4,136 (22.050)	4,213 (22.461)	0.3392	0.0099
Sulfasalazine	3,154 (16.187)	7,151 (12.939)	<0.0001	0.0922	2,973 (15.850)	2,985 (15.914)	0.8654	0.0018
Abatacept	2,393 (12.281)	1,855 (3.356)	<0.0001	0.3371	1,916 (10.215)	1,676 (8.935)	<0.0001	0.0435
Tocilizumab	1,602 (8.222)	1,032 (1.867)	<0.0001	0.2934	1,254 (6.686)	972 (5.182)	<0.0001	0.0637
Rituximab	754 (3.870)	569 (1.030)	<0.0001	0.1845	616 (3.284)	482 (2.570)	<0.0001	0.0424
Laboratory values								
C-reactive protein (TNX Curated 9063), mean ± SD	17.1 ± 33.3	16.1 ± 31.0	0.0048	0.0298	17.2 ± 33.7	16.2 ± 31.3	0.0190	0.0308
Median (IQR)	6.2 (13.7)	6.6 (13.1)	—	—	6.2 (13.8)	6.6 (13.1)	—	—
Patients with a result	12,096 (62.079)	30,695 (55.540)	—	—	11,518 (61.406)	11,620 (61.950)	—	—
Erythrocyte sedimentation rate, mm/h (TNX Curated 9066), mean ± SD	27.9 ± 24.8	27.5 ± 24.2	0.1099	0.0172	28.0 ± 24.8	27.8 ± 24.6	0.5819	0.0074
Median (IQR)	20 (30)	20 (28)	—	—	20 (29)	20 (28)	—	—
Patients with a result	11,727 (60.185)	31,482 (56.964)	—	—	11,188 (59.647)	11,132 (59.349)	—	—

Values are *n* (%) unless otherwise stated. Categorical variables were compared using Pearson’s *χ*² test and continuous variables using a two-sample *t*-test. Standardized mean differences and *P*-values were calculated from the exported counts and summary statistics; the source export provides counts, percentages, and distributional summaries only. All post-match standardized mean differences were below the conventional 0.10 threshold (maximum 0.0637). The three non-TNF biologics retained the largest residual post-match differences, all in the same direction (JAKi more exposed): tocilizumab SMD 0.0637, abatacept SMD 0.0435 and rituximab SMD 0.0424. Each remained statistically distinguishable at this cohort size (*P* < 0.0001), indicating residual channeling by disease severity in the expected direction. Baseline heart failure was well balanced in the revised cohort (7.842% vs. 8.034%; SMD 0.0071). Baseline C-reactive protein and erythrocyte sedimentation rate were both included in the propensity score model. Comparisons of the two analyte values were restricted to patients with a recorded result. The proportion of patients with a recorded result was imbalanced before matching and balanced after it: C-reactive protein 62.079% vs. 55.540% before matching (SMD 0.1331) and 61.406% vs. 61.950% after matching (SMD 0.0112); erythrocyte sedimentation rate 60.185% vs. 56.964% before matching (SMD 0.0654) and 59.647% vs. 59.349% after matching (SMD 0.0061). Erythrocyte sedimentation rate entered the propensity score model both as a continuous variable and as the strata <50, 50–100, and >100 mm/h; only the continuous summary is tabulated here.

SMD, standardized mean difference.

These differences affect pre-matching denominators only. Every estimate reported in this paper derives from a matched cohort, the propensity score was re-estimated within each cohort, and no reported comparison depends on the size of another cohort’s eligible pool. As only de-identified data were used, institutional review board approval was not required (45 CFR 46.104(d)(4)).

### Study population

We identified adults with RA (ICD-10-CM M05.x, M06.x; ICD-9-CM 714.0–714.9) initiating JAKi or TNFi between January 1, 2012 and December 31, 2024. Consistent with FDA guidance and ORAL Surveillance, inclusion required age ≥50 years and ≥1 cardiovascular risk factor (smoking, hypertension, diabetes, dyslipidemia, obesity, chronic kidney disease, or established ASCVD). We excluded prior JAKi or TNFi use, concomitant use of both classes, other inflammatory arthritides (IBD, psoriatic arthritis and ankylosing spondylitis), <365 days of baseline data, active malignancy within 5 years, or end-stage renal disease. A 365-day baseline period ensured a new-user design and covariate ascertainment; all included patients were therefore naive to both JAK and TNF inhibitors at index, with prior exposure to either class having been excluded rather than adjusted for.

### Treatment definitions

JAKi exposure comprised tofacitinib, baricitinib, and upadacitinib. TNFi exposure comprised adalimumab, etanercept, infliximab, certolizumab pegol, and golimumab.

### Outcomes

The primary outcome was MACE (nonfatal myocardial infarction or nonfatal ischemic stroke); cardiovascular death was excluded given limited EHR reliability. Secondary outcomes were all-cause mortality, heart failure hospitalization, and venous thromboembolism. Cataract was analyzed as a prespecified negative-control outcome, selected because it has no plausible mechanistic link to JAK or TNF inhibition and would therefore expose gross residual confounding by differential healthcare contact. Outcomes other than all-cause mortality were ascertained from ICD-10-CM diagnosis codes recorded after the landmark; all-cause mortality was ascertained from vital status recorded within the network. Exposures were defined by RxNorm ingredient codes and covariates and outcomes by ICD-10-CM diagnosis codes and TriNetX curated laboratory concepts, as listed in **Table 1**. Each outcome was analyzed in the full matched cohort, and patients with the outcome recorded at baseline were not excluded from the analysis of that outcome; reported estimates therefore represent the first event recorded during follow-up rather than strictly incident events. Outcome ascertainment began 90 days after initiation and continued up to 5 years or until outcome, death, loss to follow-up, or study end.

### Baseline covariates and analysis framework

To address confounding by indication, a 1:1 propensity-score-matched cohort was constructed by logistic regression. We term this the improved propensity-score model because it extends prior TriNetX analyses by adding individual disease-modifying and corticosteroid medications, prior non-TNF biologic exposure, and baseline inflammatory markers. Covariates, ascertained over the 365-day baseline window, comprised demographics (age at index, sex, race, and ethnicity), cardiovascular risk factors and comorbidities (hypertension, dyslipidemia, overweight/obesity, type 2 diabetes, nicotine dependence, tobacco use, ischemic heart disease, heart failure, atrial fibrillation, cerebral infarction, chronic kidney disease, and acute kidney failure), related rheumatologic and autoimmune diagnoses (SLE [M32], antiphospholipid syndrome [D68.61], psoriatic arthropathy [L40.5], systemic sclerosis [M34], and dermatopolymyositis [M33]), individual csDMARDs (methotrexate, hydroxychloroquine, leflunomide, and sulfasalazine), individual oral corticosteroids (prednisone, methylprednisolone, and dexamethasone), prior non-TNF biologic DMARD exposure (abatacept, tocilizumab, and rituximab), and baseline inflammatory markers (C-reactive protein, continuous, and erythrocyte sedimentation rate, continuous and stratified as <50, 50–100, and >100 mm/h). C-reactive protein and erythrocyte sedimentation rate were recorded for 11,518 (61.4%) and 11,188 (59.6%) matched JAKi initiators and 11,620 (62.0%) and 11,132 (59.3%) matched TNFi initiators, respectively, so capture was closely comparable across arms. Because inflammatory markers are recorded only when clinically ordered, roughly 40% of matched patients in each arm had no baseline value. Laboratory covariates were incorporated as values recorded during the 365-day baseline window. The platform does not impute missing laboratory data, and no imputation was performed by the investigators; patients without a recorded value therefore did not contribute to these covariates, so the absence of a result acted as an implicit category rather than being replaced by a substituted value. Availability of a result was imbalanced before matching (standardized mean difference 0.133 for C-reactive protein) and balanced after it (0.011), so differential capture is unlikely to have driven the balance achieved; adjustment for baseline inflammatory activity is nonetheless limited rather than partial: a baseline value was available for a little more than half of the cohort, and inclusion in the propensity score did not guarantee balance, as the baricitinib cohorts show. This is revisited in the “Limitations”. Matching used 1:1 nearest-neighbor matching on the logit of the propensity score (caliper 0.10 SD, greedy TriNetX implementation). Balance was assessed by standardized mean differences (SMDs), with SMD <0.10 prespecified as adequate. Separately matched cohorts using the same covariates were built for each agent (tofacitinib, baricitinib, and upadacitinib vs. TNFi), for each subgroup (sex; cardiovascular-risk-burden strata within ASCVD-free patients), and index era (pre-2021 versus post-2021 index year), including each agent within index-year strata. Each agent-specific cohort indexes on first use of that agent, so patients who switched between JAK inhibitors are eligible for more than one agent cohort; the agent cohorts therefore sum to more patients than the pooled JAKi population.

### Statistical analysis and follow-up

Time-to-event analyses compared pooled JAKi versus TNFi using Cox proportional hazards models with HRs and 95% CIs; Kaplan–Meier curves and log-rank tests compared survival. Outcomes were expressed as HRs, 5-year Kaplan–Meier cumulative incidence (100% minus 5-year survival probability), and absolute risk differences per 1,000. Hazard ratios are the primary measure of association throughout. Kaplan–Meier cumulative incidences and the absolute risk differences derived from them are reported to aid clinical interpretation, but because they are evaluated at a fixed 5-year horizon on a diminishing risk set they are more sensitive to differential availability of observation than the hazard ratios and should be read as supporting rather than primary quantities. Kaplan–Meier curves for MACE, all-cause mortality, heart failure hospitalization, and venous thromboembolism were plotted in R (ggplot2) as step functions from the platform’s day-level survival function and its 95% confidence bounds across the interval from day 90 (the landmark) to day 1,825. The platform writes a row whenever either arm’s estimate changes and leaves the unchanged arm’s cells empty; such cells denote no change rather than missing data, and the preceding value was carried forward accordingly. Every plotted point is therefore platform output, and no value is interpolated. Interval survival estimates were not exported for the three-component composite or the negative-control outcome, for which only endpoint estimates are reported and no curve is shown. The platform does not export risk-set counts, so conventional numbers-at-risk cannot be tabulated beneath the curves, and person-time at risk cannot be derived; in place of person-time, we report the duration of available electronic health record observation after the index date, which measures how long a patient’s records persist in the network rather than time at risk. Subgroup analyses used separately matched cohorts, so formal interaction testing was not performed and results are interpreted by effect-size patterns. Exposure was defined by recorded prescription orders. Follow-up was intention-to-treat, without censoring for discontinuation or switching. Proportional hazards were assessed with the proportionality test reported by the platform for each Cox model; this is a platform-generated test rather than a user-computed scaled Schoenfeld residual test. The assumption was satisfied (*p* > 0.05) for all primary and secondary outcomes in the main matched cohort and in all agent-specific, sex, cardiovascular risk, and index-year strata, with the exception of upadacitinib MACE (*p* = 0.037), upadacitinib venous thromboembolism (*p* < 0.0001), and the upadacitinib three-component composite (*p* = 0.011), heart failure hospitalization in the post-2021 stratum (*p* = 0.030), and upadacitinib post-2021 venous thromboembolism in the agent-by-era analysis (*p* = 0.013), whose HRs are interpreted as time-averaged. A prespecified sensitivity analysis used a three-component MACE composite (MI, ischemic stroke, or all-cause mortality); because cardiovascular-specific death is unreliable in TriNetX, all-cause mortality served as a pragmatic substitute, yielding an upper bound on the cardiovascular signal that incorporates non-cardiovascular deaths from infection and malignancy (9, 20, 21). E-values quantified the minimum unmeasured-confounding strength needed to explain associations. Significance was two-sided *p <*0.05; no multiple-comparison adjustment was applied, as the primary outcome was prespecified and other analyses exploratory.

## Results

### Study cohorts and propensity score matching

Of 1,290,739 RA patients in TriNetX, 752,837 met eligibility (age ≥50 years with ≥1 cardiovascular risk factor). After exclusions, 19,485 JAKi and 55,266 TNFi initiators were eligible for matching. Improved 1:1 propensity score matching yielded 18,757 matched pairs, forming the analytic cohort for the 90-day landmark analysis (**Figure 1**). Separately matched agent-specific cohorts comprised 13,078 pairs for tofacitinib, 1,720 for baricitinib, and 7,927 for upadacitinib versus TNFi. All post-matching standardized mean differences in the main cohort were below 0.1, indicating adequate balance (**Table 1**); the largest residual imbalances were in prior non-TNF biologic exposure, which was included among the matching covariates and remained modestly more frequent among JAKi initiators for all three agents—abatacept 10.215% versus 8.935% (SMD 0.044), tocilizumab 6.686% versus 5.182% (SMD 0.064), and rituximab 3.284% versus 2.570% (SMD 0.042)—a consistently directional residual indicating channeling of more refractory patients toward JAKi. Balance in the stratified cohorts was adequate with four exceptions, all confined to baricitinib. Baseline C-reactive protein remained substantially imbalanced in the post-2021 baricitinib cohort (40.4 ± 60.4 versus 21.7 ± 37.3 mg/L; SMD 0.373; **Supplementary Table S7**), the largest residual difference in any cohort in this study, and in the pooled baricitinib agent cohort (33.3 ± 54.9 versus 19.8 ± 39.1 mg/L; SMD 0.283; **Supplementary Table S2**). Prior abatacept exposure remained imbalanced in the pre-2021 baricitinib cohort (16.6% versus 12.6%; SMD 0.115; **Supplementary Table S6**). These three residuals all lie in the direction of greater disease severity or refractoriness among baricitinib initiators, indicating that this agent entered every comparison with a higher inflammatory and treatment history burden than any available matched TNFi population despite both markers being included in the propensity score. The fourth exception, erythrocyte sedimentation rate in the post-2021 baricitinib cohort (29.9 ± 25.2 versus 32.7 ± 27.7 mm/h; SMD 0.102), ran in the opposite direction; the two markers were recorded in partly non-overlapping subsets of patients, and the C-reactive protein distribution in this cohort was strongly right-skewed, consistent with a subgroup with acute intercurrent illness at index. All baricitinib estimates are interpreted in light of these residuals. A small number of standardized mean differences elsewhere in the stratified cohorts exceed 0.10 only because one arm contained fewer than the platform’s minimum reportable count, which is returned as 10 or 0; these reflect count obfuscation rather than measurable imbalance and are flagged in the corresponding supplementary tables. Baseline characteristics for the agent-specific, sex-stratified, cardiovascular-risk-stratified, and index-era matched cohorts appear in **Supplementary Tables S1**–**S16**. The mean duration of available electronic health record observation after the index date was 1,171.5 ± 652.5 days for JAKi and 1,080.0 ± 651.7 days for TNFi (median 1,320 versus 1,111 days; **Supplementary Table S17**). This metric reflects how long a patient’s records persist in the network rather than person-time at risk, which cannot be derived from the platform’s exports; the longer availability of observation in the JAKi arm is considered in the “Limitations”.

**Figure 1:**
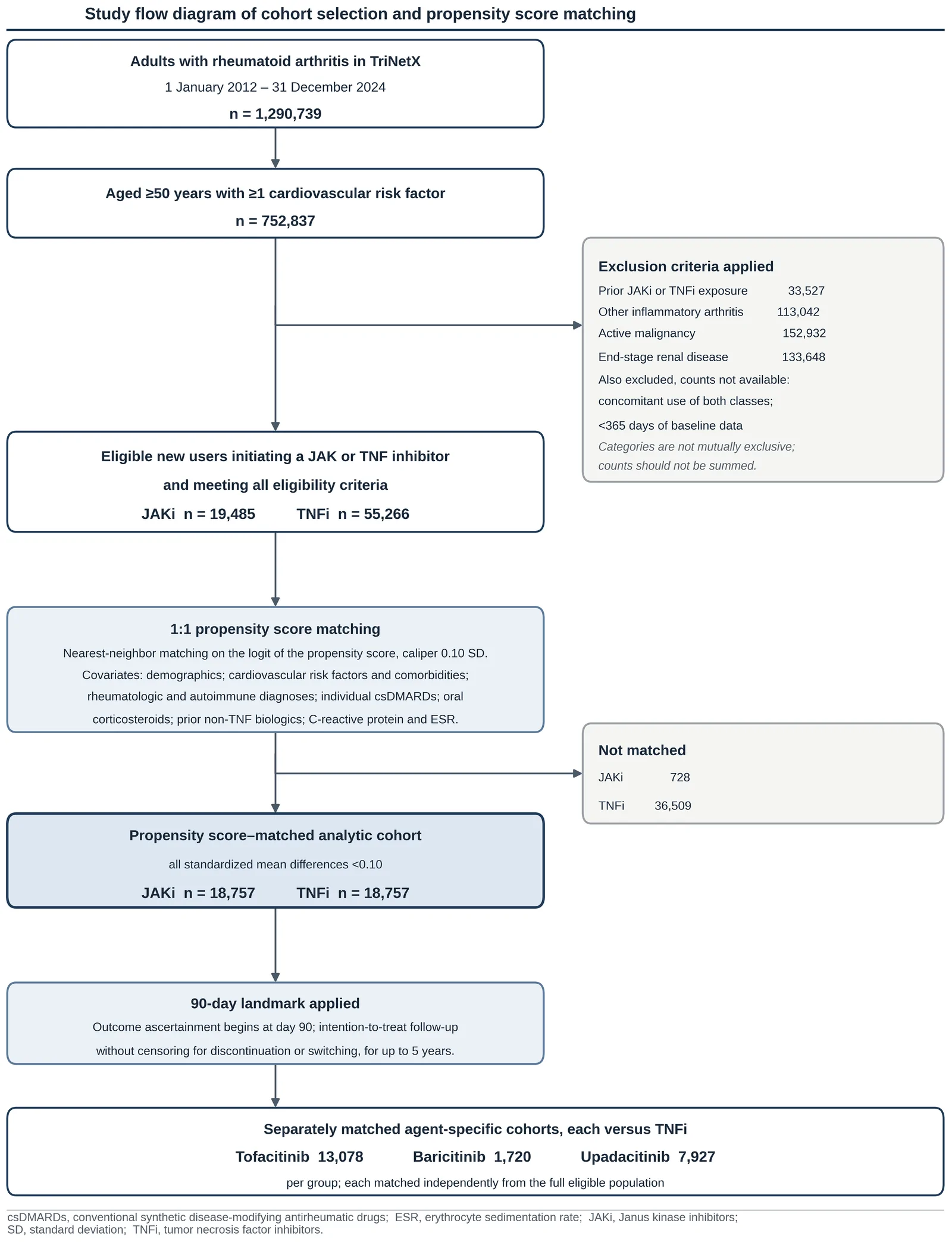
Study flow diagram of cohort selection and propensity score matching. Of 1,290,739 patients with rheumatoid arthritis identified within the TriNetX federated electronic health record network between January 1, 2012 and December 31, 2024, 752,837 met the prespecified eligibility criteria (age ≥50 years with at least one cardiovascular risk factor). Patients were excluded for prior JAKi or TNFi exposure (*n* = 33,527), other inflammatory arthritides (*n* = 113,042), active malignancy (*n* = 152,932), or end-stage renal disease (*n* = 133,648); patients with concomitant use of both drug classes or fewer than 365 days of baseline data were also excluded, but per-criterion counts for these two categories are not available. Exclusion categories are not mutually exclusive, so the individual counts should not be summed. The remaining eligible patients who went on to initiate one of the two drug classes comprised 19,485 JAKi initiators and 55,266 TNFi initiators available for matching. Improved 1:1 nearest-neighbor propensity score matching (caliper 0.10 SD on the logit of the propensity score), incorporating demographics, cardiovascular risk factors, comorbidities, rheumatologic and autoimmune diagnoses, individual conventional synthetic DMARDs (methotrexate, hydroxychloroquine, leflunomide, and sulfasalazine), individual oral corticosteroids (prednisone, methylprednisolone, and dexamethasone), prior non-TNF biologic exposure (abatacept, tocilizumab, and rituximab), and baseline inflammatory markers (C-reactive protein and erythrocyte sedimentation rate), yielded 18,757 matched pairs with all standardized mean differences below 0.10. Outcome ascertainment began 90 days after treatment initiation (landmark analysis), with intention-to-treat–style follow-up for up to 5 years. Separately constructed 1:1 propensity-score-matched agent-specific cohorts were generated for tofacitinib (*n* = 13,078 per group), baricitinib (*n* = 1,720 per group), and upadacitinib (*n* = 7,927 per group), each compared with TNFi and each matched independently from the full eligible population rather than by subsetting the main matched cohort. csDMARDs, conventional synthetic disease-modifying antirheumatic drugs; ESR, erythrocyte sedimentation rate; JAKi, Janus kinase inhibitors; RA, rheumatoid arthritis; SD, standard deviation; TNFi, tumor necrosis factor inhibitors.

### Primary outcome: MACE

In the matched cohort (18,757 per group), 1,191 MACE events occurred among JAKi and 1,109 among TNFi initiators. JAKi was not associated with an increase in MACE (HR 1.002, 95% CI 0.923–1.088; log-rank *p* = 0.9579). The 5-year Kaplan–Meier cumulative incidence was 9.474% versus 9.263%, an absolute difference of +0.211% (+2.11 events per 1,000 over 5 years; NS). The proportional hazards assumption was satisfied (proportionality *p* = 0.192) (**Table 2**; **Figure 2**).

**Table 2:** Primary and secondary outcomes in the main matched cohort.

Outcome	Events, JAKi	Events, TNFi	Cum. incidence, % JAKi	Cum. incidence, % TNFi	Δ per 1,000	HR	95% CI	Log-rank *P*	PH *P*
MACE (myocardial infarction or ischemic stroke)	1,191	1,109	9.474	9.263	+2.11	1.002	0.923–1.088	0.9579	0.1918
All-cause mortality	1,071	843	8.922	7.881	+10.41	1.158	1.058–1.268	0.0014	0.2981
Heart failure hospitalization	1,887	1,753	14.263	13.887	+3.76	1.019	0.955–1.088	0.5653	0.4684
Venous thromboembolism	954	849	7.459	6.931	+5.28	1.054	0.961–1.156	0.2631	0.1724
3-component MACE (including death)	2,032	1,780	16.151	15.267	+8.84	1.058	0.992–1.127	0.0844	0.6141
Cataract (negative-control outcome)	1,386	1,387	10.689	11.299	−6.10	0.934	0.867–1.007	0.0739	0.5348

*n* = 18,757 per group. Five-year Kaplan–Meier estimates with Cox proportional hazards ratios; JAKi versus TNFi. Cumulative incidence is derived from the Kaplan–Meier survival function rather than from crude event proportions. The proportional hazards assumption is satisfied for every outcome in the main cohort (all *P* ≥ 0.17). Cataract was prespecified as a negative-control outcome. Its hazard ratio lies near unity and, if anything, favors JAKi, providing no evidence that differential surveillance inflated event capture in the JAKi arm. Crude event counts were near-identical (1,386 versus 1,387); the negative Kaplan–Meier risk difference therefore reflects differential follow-up rather than a difference in the number of events observed. Hazard ratios are the primary measure of association; five-year cumulative incidences and the derived risk differences are supporting quantities and are more sensitive to differential availability of observation.

MACE, major adverse cardiovascular events (myocardial infarction or ischemic stroke); PH, proportional hazards.

**Figure 2:**
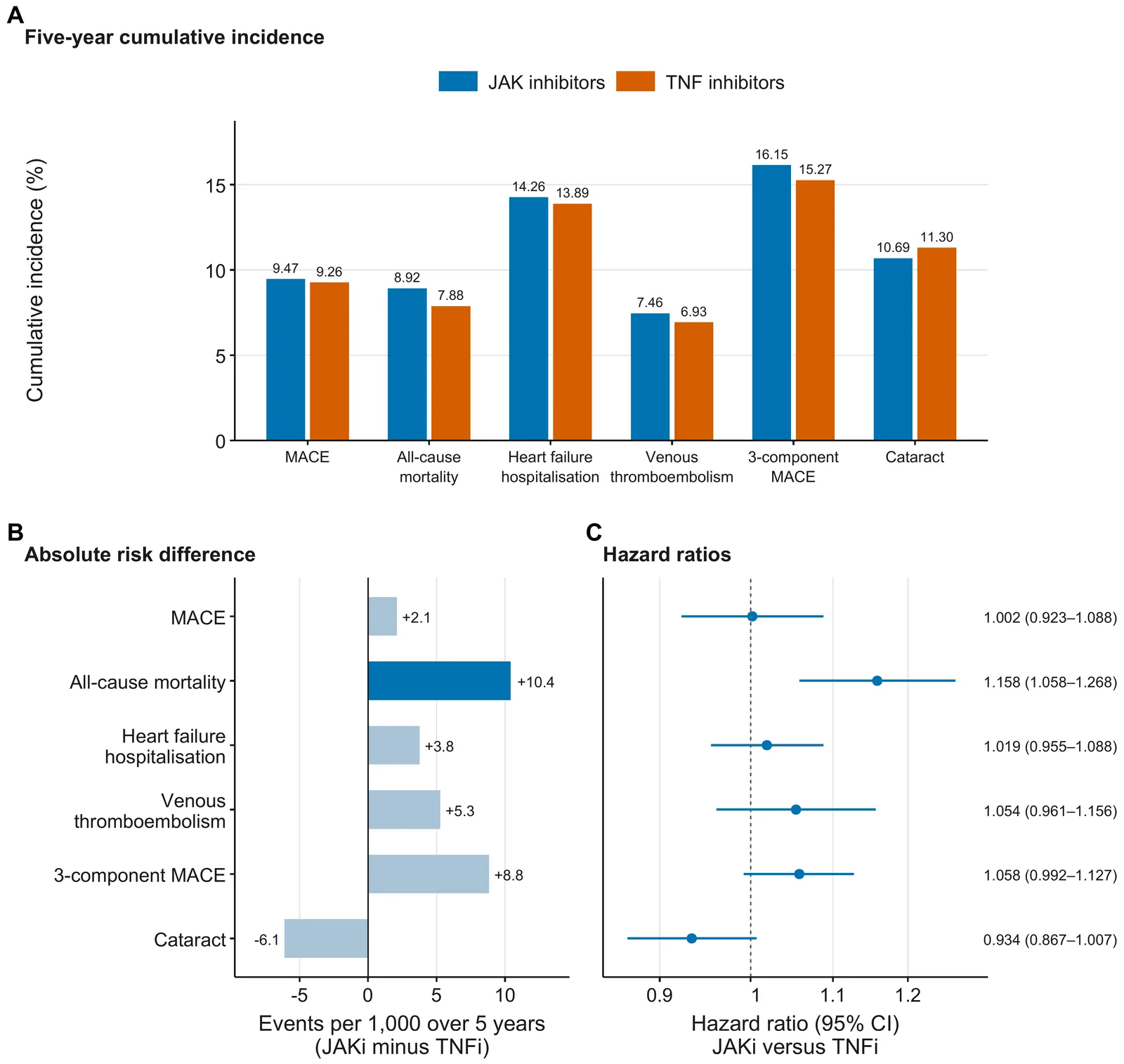
Cardiovascular and mortality outcomes comparing JAK inhibitors with TNF inhibitors in patients with rheumatoid arthritis: improved propensity-score-matched analysis (*n* = 18,757 pairs). **(A)** Five-year Kaplan–Meier cumulative incidence of major adverse cardiovascular events (MACE; nonfatal myocardial infarction or ischemic stroke), all-cause mortality, heart failure hospitalization, venous thromboembolism, the three-component MACE composite (nonfatal myocardial infarction, ischemic stroke, or all-cause mortality), and the prespecified negative-control outcome of cataract in JAKi versus TNFi initiators. **(B)** Absolute risk differences expressed as additional events per 1,000 patients over 5 years (JAKi minus TNFi) on the Kaplan–Meier scale. **(C)** Forest plot of hazard ratios with 95% confidence intervals derived from Cox proportional hazards models. The 5-year Kaplan–Meier cumulative incidence values were MACE 9.474% vs. 9.263% (HR 1.002, 95% CI 0.923–1.088; +2.11/1,000; NS), heart failure hospitalization 14.263% vs. 13.887% (HR 1.019, 95% CI 0.955–1.088; +3.76/1,000; NS), venous thromboembolism 7.459% vs. 6.931% (HR 1.054, 95% CI 0.961–1.156; +5.28/1,000; NS), all-cause mortality 8.922% vs. 7.881% (HR 1.158, 95% CI 1.058–1.268; +10.41/1,000), and the three-component MACE composite 16.151% vs. 15.267% (HR 1.058, 95% CI 0.992–1.127; +8.84/1,000; NS). The prespecified negative-control outcome, cataract, showed no excess (10.689% vs. 11.299%; HR 0.934, 95% CI 0.867–1.007; −6.10/1,000; NS). Follow-up began 90 days after treatment initiation (landmark analysis) and continued for up to 5 years with intention-to-treat–style censoring; statistical significance was set at log-rank *p <*0.05. Data source: TriNetX global federated research network. Improved 1:1 nearest-neighbor propensity score matching (caliper 0.10 SD on the logit of the propensity score) incorporated demographics, cardiovascular risk factors, RA-related and rheumatologic diagnoses, comorbidities, individual conventional synthetic DMARDs (methotrexate, hydroxychloroquine, leflunomide, and sulfasalazine), individual oral corticosteroids (prednisone, methylprednisolone, and dexamethasone), prior non-TNF biologic exposure (abatacept, tocilizumab, and rituximab), and baseline inflammatory markers (C-reactive protein and erythrocyte sedimentation rate); this yielded 18,757 well-balanced pairs from the pre-matching populations of 19,485 JAKi and 55,266 TNFi initiators, with all post-matching standardized mean differences <0.10, indicating adequate covariate balance. CI, confidence interval; csDMARDs, conventional synthetic disease-modifying antirheumatic drugs; ESR, erythrocyte sedimentation rate; HR, hazard ratio; JAKi, Janus kinase inhibitors; KM, Kaplan–Meier; MACE, major adverse cardiovascular events; NS, not statistically significant (log-rank *p* ≥ 0.05); RA, rheumatoid arthritis; SD, standard deviation; SMD, standardized mean difference; TNFi, tumor necrosis factor inhibitors; VTE, venous thromboembolism.

### Key secondary outcomes

All-cause mortality was significantly higher with JAKi (1,071 vs. 843 deaths; HR 1.158, 95% CI 1.058–1.268; *p* = 0.0014; 5-year cumulative incidence 8.922% vs. 7.881%; +10.41 per 1,000). Neither heart failure hospitalization (1,887 vs. 1,753 events; HR 1.019, 95% CI 0.955–1.088; *p* = 0.5653; 14.263% vs. 13.887%; +3.76 per 1,000) nor venous thromboembolism (954 vs. 849 events; HR 1.054, 95% CI 0.961–1.156; *p* = 0.2631; 7.459% vs. 6.931%; +5.28 per 1,000) differed significantly. The prespecified negative-control outcome, cataract, showed no excess with JAKi (1,386 vs. 1,387 events; HR 0.934, 95% CI 0.867–1.007; *p* = 0.0739; 10.689% vs. 11.299%; −6.10 per 1,000), arguing against gross residual confounding by overall healthcare contact. Proportional hazards tests were satisfied for all secondary outcomes (*p* > 0.05) (**Table 2**; **Figure 2**; **Supplementary Figure S1**).

### Drug-specific JAKi analyses

In separately matched cohorts (**Table 3**; **Figure 3**; **Supplementary Figures S2**, **S5**), no agent showed a significant MACE excess. Tofacitinib (*n* = 13,078 per group) showed a small, non-significant increase (988 vs. 861 events; HR 1.038, 95% CI 0.948–1.138; *p* = 0.4202; 5-year MACE-free survival 90.110% vs. 90.573%). Baricitinib (*n* = 1,720 per group) had a larger point estimate but remained non-significant (145 vs. 130 events; HR 1.084, 95% CI 0.855–1.373; *p* = 0.5065; 86.188% vs. 87.515%). Upadacitinib (*n* = 7,927 per group) was numerically lower, without significance (362 vs. 374 events; HR 0.870, 95% CI 0.753–1.006; *p* = 0.0597; 90.631% vs. 90.589%); the proportional hazards assumption was violated for this comparison (proportionality *p* = 0.037), so the estimate is interpreted as time-averaged.

**Table 3:** Agent-specific outcomes, all index years combined.

Outcome	Events, JAKi	Events, TNFi	Survival, % JAKi	Survival, % TNFi	HR	95% CI	Log-rank *P*	PH *P*
Tofacitinib—*n* = 13,078 per group								
MACE	988	861	90.110	90.573	1.038	0.948–1.138	0.4202	0.3741
All-cause mortality	879	738	91.104	91.391	1.052	0.954–1.160	0.3105	0.1352
Heart failure hospitalization	1,479	1,306	85.739	86.350	1.044	0.969–1.125	0.2531	0.7755
Venous thromboembolism	790	690	92.208	92.649	1.044	0.943–1.156	0.4071	0.2372
3-component MACE	1,678	1,465	83.430	83.870	1.028	0.958–1.102	0.4475	0.8838
Baricitinib—*n* = 1,720 per group								
MACE	145	130	86.188	87.515	1.084	0.855–1.373	0.5065	0.7221
All-cause mortality	135	105	87.034	88.822	1.213	0.939–1.565	0.1381	0.4904
Heart failure hospitalization	238	248	79.378	78.376	0.931	0.779–1.113	0.4320	0.5866
Venous thromboembolism	115	83	89.371	92.706	1.364	1.028–1.809	0.0306	0.2386
3-component MACE	247	219	77.286	78.987	1.091	0.909–1.308	0.3500	0.9513
Upadacitinib—*n* = 7,927 per group								
MACE	362	374	90.631	90.589	0.870	0.753–1.006	0.0597	0.0370^a^
All-cause mortality	269	218	92.217	93.251	1.090	0.912–1.303	0.3441	0.3100
Heart failure hospitalization	632	608	86.237	85.513	0.953	0.852–1.065	0.3935	0.9344
Venous thromboembolism	308	299	92.579	92.953	0.932	0.795–1.093	0.3867	<0.0001^a^
3-component MACE	570	551	84.966	85.455	0.922	0.820–1.037	0.1767	0.0106^a^

Each agent cohort was constructed and matched 1:1 to TNFi initiators independently. Five-year Kaplan–Meier estimates with Cox hazard ratios; JAKi versus TNFi. Each agent-specific cohort was constructed and propensity-score-matched independently from the outset, drawing on the full eligible population rather than on the main matched cohort. The agent-specific cohort sizes are therefore not expected to sum to the main matched cohort, and the same TNFi initiator may serve as a comparator in more than one agent analysis; the three agent analyses are consequently not statistically independent of one another. No agent-specific mortality signal reaches significance in time-to-event analysis (tofacitinib *P* = 0.311, baricitinib *P* = 0.138, and upadacitinib *P* = 0.344). The baricitinib venous thromboembolism association rests on 115 versus 83 events in the smallest agent cohort and is reported as hypothesis-generating; baseline C-reactive protein remained imbalanced after matching in this cohort alone among the three agent-specific cohorts (mean 33.3 versus 19.8 mg/L; standardized mean difference 0.283), indicating residual confounding by disease activity in the direction that would inflate this estimate. Survival is derived from the Kaplan–Meier survival function rather than from crude event proportions. Hazard ratios are the primary measure of association; five-year Kaplan–Meier survival estimates are supporting quantities and are more sensitive to differential availability of observation.

CI, confidence interval; HR, hazard ratio; MACE, major adverse cardiovascular events (myocardial infarction or ischemic stroke); PH, proportional hazards.

^a^ Proportional hazards assumption violated (*P* < 0.05). Hazard ratios for upadacitinib MACE, venous thromboembolism, and three-component MACE are time-averaged summaries over a period in which the survival curves converge or cross and should not be read as constant effects. For venous thromboembolism and three-component MACE, the hazard ratio favors upadacitinib while both the crude event counts and the 5-year Kaplan–Meier estimates favor the comparator; the Kaplan–Meier estimates should be preferred for these two outcomes.

**Figure 3:**
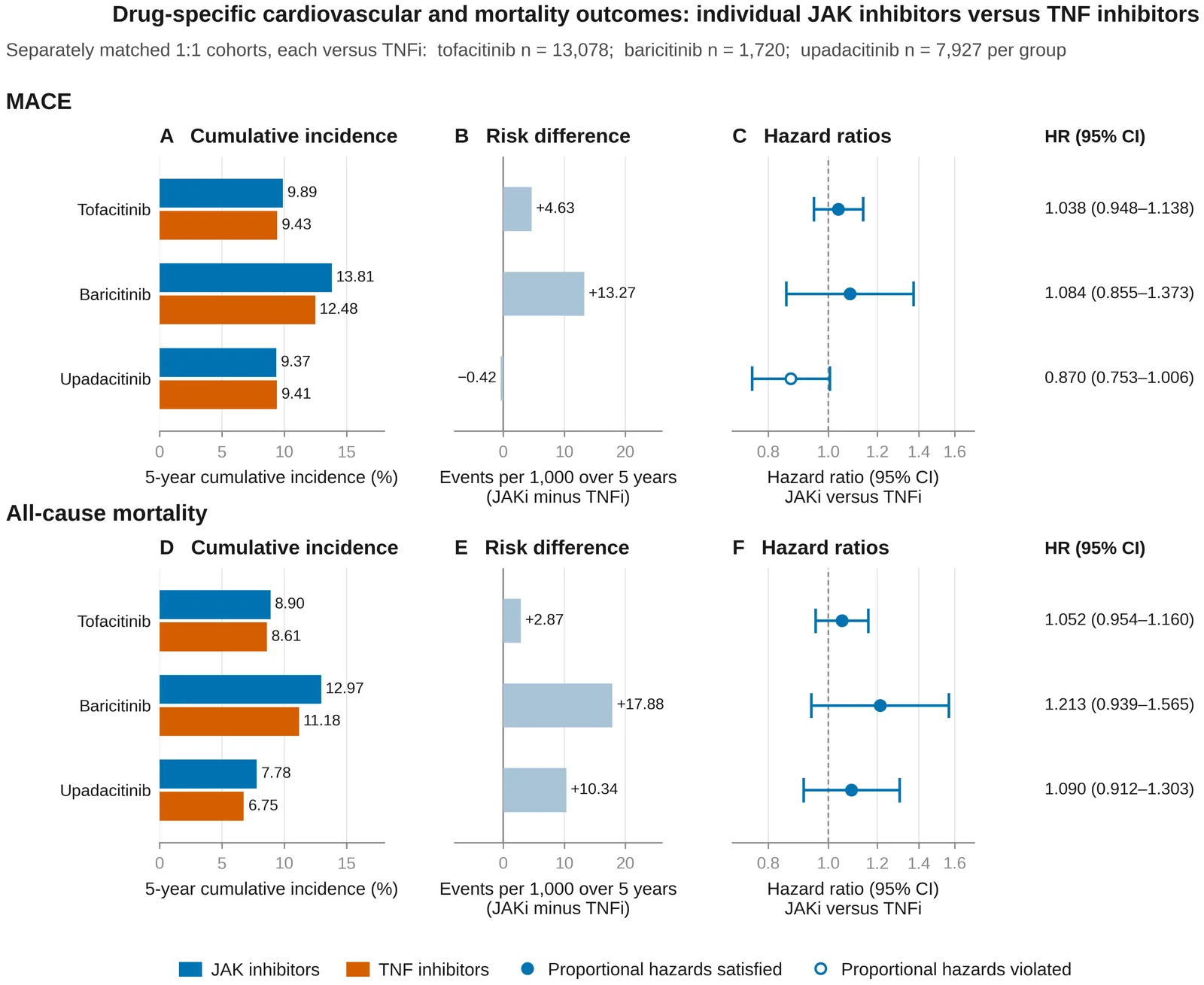
Drug-specific cardiovascular and mortality outcomes: individual JAK inhibitors versus TNF inhibitors in patients with rheumatoid arthritis. Separately constructed 1:1 improved propensity-score-matched cohorts compared tofacitinib (*n* = 13,078 per group), baricitinib (*n* = 1,720 per group), and upadacitinib (*n* = 7,927 per group) initiators with TNFi initiators. **(A)** Five-year Kaplan–Meier cumulative incidence of MACE. **(B)** Absolute risk difference for MACE (JAKi minus TNFi). **(C)** Forest plot of hazard ratios for MACE. **(D)** Five-year Kaplan–Meier cumulative incidence of all-cause mortality. **(E)** Absolute risk difference for all-cause mortality. **(F)** Forest plot of hazard ratios for all-cause mortality. Agents appear in the same order in every panel. The 5-year Kaplan–Meier cumulative incidence values were as follows: MACE—tofacitinib 9.890% vs. 9.427% (HR 1.038, 95% CI 0.948–1.138; +0.46%, +4.63/1,000; NS), baricitinib 13.812% vs. 12.485% (HR 1.084, 95% CI 0.855–1.373; +1.33%, +13.27/1,000; NS), and upadacitinib 9.369% vs. 9.411% (HR 0.870, 95% CI 0.753–1.006; −0.04%, −0.42/1,000; NS). All-cause mortality—tofacitinib 8.896% vs. 8.609% (HR 1.052, 95% CI 0.954–1.160; +0.29%, +2.87/1,000; NS), baricitinib 12.966% vs. 11.178% (HR 1.213, 95% CI 0.939–1.565; +1.79%, +17.88/1,000; NS), and upadacitinib 7.783% vs. 6.749% (HR 1.090, 95% CI 0.912–1.303; +1.03%, +10.34/1,000; NS). Follow-up began 90 days after treatment initiation (landmark analysis); intention-to-treat-style analysis was applied with up to 5 years of follow-up. Statistical significance was set at log-rank *p <*0.05. Tofacitinib has predominant JAK1/JAK3 activity, baricitinib has JAK1/JAK2 activity, and upadacitinib is preferentially JAK1-selective; direct comparisons between agents are limited because each comparison used a separately constructed matched cohort. Open markers denote comparisons in which the proportional hazards assumption was violated on the platform proportionality test—here upadacitinib MACE (*p* = 0.037)—whose hazard ratios are interpreted as time-averaged estimates. CI, confidence interval; HR, hazard ratio; JAKi, Janus kinase inhibitors; KM, Kaplan–Meier; MACE, major adverse cardiovascular events (nonfatal myocardial infarction or ischemic stroke); NS, not statistically significant (log-rank *p* ≥ 0.05); TNFi, tumor necrosis factor inhibitors.

All-cause mortality did not differ significantly for any individual agent. Tofacitinib showed no excess (879 vs. 738 deaths; HR 1.052, 95% CI 0.954–1.160; *p* = 0.3105; 5-year survival 91.104% vs. 91.391%). Baricitinib had the largest point estimate (135 vs. 105 deaths; HR 1.213, 95% CI 0.939–1.565; *p* = 0.1381; 87.034% vs. 88.822%), though the wider confidence interval and smaller sample warrant caution. Upadacitinib showed no significant difference (269 vs. 218 deaths; HR 1.090, 95% CI 0.912–1.303; *p* = 0.3441; 92.217% vs. 93.251%). Heart failure hospitalization and the three-component composite were non-significant for all three agents. The proportional hazards assumption was violated for upadacitinib venous thromboembolism (proportionality *p <*0.0001) and for the upadacitinib three-component composite (*p* = 0.011), so both estimates are interpreted as time-averaged. The only significant agent-specific finding was an excess of venous thromboembolism with baricitinib (115 vs. 83 events; HR 1.364, 95% CI 1.028–1.809; *p* = 0.0306; 89.371% vs. 92.706%), which rests on 198 events and is therefore hypothesis-generating.

### Subgroup analyses

The strata were matched separately, so interaction was not formally tested and estimates are compared descriptively. Sex-stratified analyses (**Table 4**; **Supplementary Figure S3**) showed no significant MACE differences in either sex. Among women (*n* = 15,551 per group), 5-year MACE-free survival was 90.930% versus 91.319% (HR 1.028, 95% CI 0.938–1.127; *p* = 0.5534), and among men (*n* = 3,983 per group) it was 87.408% versus 87.516% (HR 1.005, 95% CI 0.860–1.174; *p* = 0.9485). All-cause mortality was significantly higher with JAKi among women (815 vs. 663 deaths; HR 1.123, 95% CI 1.013–1.244; *p* = 0.0270; 91.962% vs. 92.711%) and showed a non-significant trend in the same direction among men (HR 1.161, 95% CI 0.986–1.368; *p* = 0.0732; 87.214% vs. 88.452%). The three-component composite was borderline in women (HR 1.065, 95% CI 0.991–1.144; *p* = 0.0879) and non-significant in men (HR 1.066, 95% CI 0.946–1.200; *p* = 0.2938).

**Table 4:** Prespecified subgroup and index-year analyses of 5-year outcomes after JAK inhibitor versus TNF inhibitor initiation in independently propensity-score-matched strata^a^.

Stratum/outcome	Events, JAKi/TNFi^b^	5-yr survival, % JAKi/TNFi^c^	Δ per 1,000^d^	HR (95% CI)^e^	Log-rank *P*	PH *P*
Sex						
Female (*n* = 15,551)						
MACE	955/866	90.930/91.319	+3.89	1.028 (0.938–1.127)	0.5534	0.2923
All-cause mortality	815/663	91.962/92.711	+7.49	1.123 (1.013–1.244)	0.0270	0.3359
3-component MACE	1,600/1,394	84.850/85.877	+10.27	1.065 (0.991–1.144)	0.0879	0.2776
Male (*n* = 3,983)						
MACE	328/308	87.408/87.516	+1.08	1.005 (0.860–1.174)	0.9485	0.8913
All-cause mortality	324/257	87.214/88.452	+12.38	1.161 (0.986–1.368)	0.0732	0.3281
3-component MACE	582/511	78.008/78.953	+9.45	1.066 (0.946–1.200)	0.2938	0.7139
ASCVD-free cardiovascular risk burden^f,g^						
1 risk factor (*n* = 5,880)						
MACE	122/100	96.478/97.028	+5.50	1.126 (0.865–1.467)	0.3778	0.2456
All-cause mortality	189/189	94.670/94.328	−3.42	0.919 (0.751–1.124)	0.4103	0.5370
3-component MACE	286/273	92.021/91.906	−1.15	0.966 (0.818–1.140)	0.6812	0.4753
2 risk factors (*n* = 4,550)						
MACE	162/129	94.363/95.167	+8.04	1.139 (0.904–1.436)	0.2696	0.5357
All-cause mortality	225/184	92.394/93.249	+8.55	1.109 (0.913–1.348)	0.2972	0.4340
3-component MACE	357/286	87.902/89.475	+15.73	1.133 (0.970–1.324)	0.1149	0.3558
≥3 risk factors (*n* = 2,508)						
MACE	124/101	91.965/93.204	+12.39	1.119 (0.860–1.456)	0.4010	0.1093
All-cause mortality	134/136	91.562/90.921	−6.41	0.896 (0.706–1.138)	0.3694	0.2504
3-component MACE	232/211	85.587/86.233	+6.46	1.007 (0.836–1.213)	0.9419	0.1160
Index year^h^						
Pre-2021 (*n* = 8,437)						
MACE	744/790	90.212/89.688	−5.24	0.946 (0.856–1.046)	0.2807	0.9927
All-cause mortality	716/670	90.682/91.296	+6.14	1.079 (0.971–1.199)	0.1560	0.2549
Heart failure hospitalization	1,068/1,016	86.134/86.926	+7.92	1.063 (0.976–1.159)	0.1614	0.7083
Venous thromboembolism	606/576	92.069/92.493	+4.24	1.061 (0.947–1.190)	0.3058	0.5287
3-component MACE	1,313/1,307	83.084/83.231	+1.47	1.010 (0.936–1.090)	0.7981	0.9011
Post-2021 (*n* = 10,230)						
MACE	493/529	89.970/89.648	−3.22	0.974 (0.862–1.102)	0.6796	0.6754
All-cause mortality	378/372	91.295/91.766	+4.71	1.061 (0.920–1.225)	0.4145	0.2105
Heart failure hospitalization^i^	888/901	84.401/83.955	−4.46	1.042 (0.950–1.144)	0.3795	0.0295
Venous thromboembolism	406/431	92.147/91.675	−4.72	0.988 (0.862–1.131)	0.8573	0.4438
3-component MACE	792/826	83.519/83.705	+1.86	1.003 (0.909–1.105)	0.9587	0.6695

Follow-up began at the 90-day landmark and continued for up to 5 years; all analyses are intention-to-treat. Three-component MACE comprises myocardial infarction, ischemic stroke, or all-cause mortality.

ASCVD, atherosclerotic cardiovascular disease; CI, confidence interval; HR, hazard ratio; JAKi, Janus kinase inhibitors; MACE, major adverse cardiovascular events (myocardial infarction or ischemic stroke); PH, proportional hazards; RF, risk factor; TNFi, tumor necrosis factor inhibitors; VTE, venous thromboembolism.

^a^ Each stratum was constructed and propensity-score-matched independently from the outset, not derived by subsetting the main matched cohort (18,757 pairs). Stratum sizes do not sum to the main cohort for two reasons. The propensity score was re-estimated within each stratum, so a patient unmatched in the main model may be matched in a stratum model and *vice versa*, and the stratified cohorts were extracted separately from the main cohort (Methods; **Supplementary Table S21**). Sex strata total 19,534 pairs, ASCVD-free risk strata 12,938, and index-year strata 18,667. The same comparator may appear in more than one stratum, and stratum estimates are not constrained to bracket the main-cohort estimate.

^b^ Crude counts across the whole window, unadjusted for differential follow-up time; they may diverge from time-to-event estimates. In the one-risk-factor stratum, mortality events were identical (189 versus 189; crude *P* = 1.0000), yet the Kaplan–Meier difference is −3.42 per 1,000 and the hazard ratio 0.919. The Kaplan–Meier convention is used throughout for consistency with the Cox models.

^c^ Kaplan–Meier estimate at 5 years, from the survival function rather than crude event proportions. Cumulative incidence is 100 minus the value shown.

^d^ (TNFi survival − JAKi survival) × 10, per 1,000 patients. Positive values denote higher risk with JAK inhibitors.

^e^ Interaction was not formally tested, as strata were matched separately and are not directly comparable. *P*-values are descriptive and unadjusted for multiplicity.

^f^ Patients without established atherosclerotic cardiovascular disease at baseline, stratified by count of baseline risk factors (smoking, hypertension, diabetes, dyslipidemia, obesity, and chronic kidney disease). Parent-cohort eligibility required at least one risk factor, so the lowest stratum comprises patients with exactly one; no risk-factor-free stratum exists.

^g^ Neither outcome shows a monotonic gradient across increasing risk burden: MACE 1.126, 1.139, and 1.119 and mortality 0.919, 1.109, and 0.896 across one, two, and three or more risk factors. All six confidence intervals include unity.

^h^ Defined by calendar year of initiation (pre-2021, index date before January 1, 2021; post-2021, on or after). Every patient has an index date and falls into exactly one era.

^i^ Proportional hazards were violated (proportionality *P* = 0.0295), the only violation in this table; the hazard ratio is a weighted average over the window, not a constant effect. Its direction (1.042) is also opposite to both the crude counts (888 versus 901) and the Kaplan–Meier difference (−4.46 per 1,000), each favoring JAK inhibitors.

### Risk-gradient analysis in ASCVD-free patients

Among ASCVD-free patients, no stratum showed a significant MACE difference (**Table 4**), though absolute 5-year risks rose steeply with cardiovascular burden—one risk factor (*n* = 5,880 per group): 5-year MACE-free survival 96.478% versus 97.028% (HR 1.126, 95% CI 0.865–1.467; *p* = 0.3778), two risk factors (*n* = 4,550 per group): 94.363% versus 95.167% (HR 1.139, 95% CI 0.904–1.436; *p* = 0.2696), and three or more risk factors (*n* = 2,508 per group): 91.965% versus 93.204% (HR 1.119, 95% CI 0.860–1.456; *p* = 0.4010). The MACE point estimates were therefore consistently near 1.12 across strata, without a monotonic gradient. All-cause mortality showed no significant difference in any stratum and no consistent direction (one risk factor HR 0.919, 95% CI 0.751–1.124; two risk factors HR 1.109, 95% CI 0.913–1.348; and ≥3 risk factors HR 0.896, 95% CI 0.706–1.138) as did the three-component composite. All proportional hazards tests were satisfied (*p* > 0.05).

### Index-year strata

Because the JAKi prescribing patterns changed after the 2021 boxed warning, separately matched cohorts were built by index era (**Table 4**). The two era cohorts comprise 18,667 pairs in total rather than 18,757. Every patient has an index date and falls into exactly one era, so the 90-pair difference does not reflect any patient being excluded; it arises because the propensity score was re-estimated within each era so that a patient left unmatched in the main model may be matched in an era model and vice versa and because the era cohorts were extracted separately from the main cohort (“Methods”; **Supplementary Table S21**; **Table 4**, footnote a). Neither era showed a significant difference in MACE (pre-2021, *n* = 8,437 per group: HR 0.946, 95% CI 0.856–1.046; post-2021, *n* = 10,230 per group: HR 0.974, 95% CI 0.862–1.102), all-cause mortality (HR 1.079, 95% CI 0.971–1.199 and HR 1.061, 95% CI 0.920–1.225, respectively), heart failure hospitalization, venous thromboembolism, or the three-component composite; the proportional hazards assumption was violated only for post-2021 heart failure hospitalization (*p* = 0.030). Agent-by-era analyses (**Supplementary Table S18**; **Supplementary Figure S4**) yielded 'three significant clusters: tofacitinib pre-2021 all-cause mortality (HR 1.123, 95% CI 1.004-1.256; *p* = 0.0428); baricitinib pre-2021 all-cause mortality (HR 2.271, 95% CI 1.432-3.602; *p* = 0.0003) and three-component MACE (HR 1.540, 95% CI 1.119-2.121; *p* = 0.0077), both derived from only 469 pairs and 85 and 157 events, respectively; and lower risk with upadacitinib post-2021 for MACE (HR 0.825, 95% CI 0.700–0.973; *p* = 0.0221), all-cause mortality (HR 0.815, 95% CI 0.667–0.996; *p* = 0.0451), and three-component MACE (HR 0.831, 95% CI 0.727–0.950; *p* = 0.0065). The proportional hazards assumption held across the agent-by-era comparisons with one exception, upadacitinib post-2021 venous thromboembolism (*p* = 0.013), whose hazard ratio is interpreted as time-averaged. Because agent and index era are strongly correlated with calendar time prescribing, these estimates are era-confounded and hypothesis-generating.

### Sensitivity analysis

In a prespecified three-component MACE composite (MI, ischemic stroke, or all-cause mortality), JAKi showed a non-significant excess (2,032 vs. 1,780 events; 16.151% vs. 15.267%; HR 1.058, 95% CI 0.992–1.127; *p* = 0.0844; +8.84 per 1,000), directionally consistent with the mortality signal but not confirming a discrete cardiovascular excess; this contrasts with the fully null two-component result (HR 1.002; *p* = 0.9579) (**Table 2**; **Figure 2**). In the main matched cohort, the proportional hazards assumption held for both composites (*p* > 0.05). E-values indicated that the surviving associations require only modest unmeasured confounding to explain: main-cohort all-cause mortality had an E-value of 1.586 (1.306 at the confidence limit) and female all-cause mortality 1.495 (1.128). Larger E-values attached to the small-sample, era-stratified estimates (**Supplementary Table S19**), including baricitinib pre-2021 mortality (3.970; 2.219 at the limit), baricitinib pre-2021 three-component MACE (2.452; 1.484), and baricitinib venous thromboembolism (2.069; 1.198). The E-values for the remaining nominally significant era-stratified estimates were more modest—tofacitinib pre-2021 mortality 1.495 (1.067 at the limit) and, for the protective upadacitinib post-2021 estimates, 1.719 (1.197) for MACE, 1.755 (1.068) for all-cause mortality, and 1.698 (1.288) for the three-component composite—indicating that these associations could likewise be accounted for by modest unmeasured confounding.

## Discussion

In this 5-year target trial emulation of older RA patients with cardiovascular risk factors, JAKi initiation was associated with a modest excess in all-cause mortality (HR 1.158; +10.4 per 1,000 over 5 years) versus TNFi. After improved propensity-score matching incorporating individual csDMARDs, corticosteroids, prior non-TNF biologic exposure, and C-reactive protein, the apparent MACE excess was fully attenuated (HR 1.002), and neither heart failure hospitalization (HR 1.019) nor VTE (HR 1.054) differed. A prespecified negative-control outcome, cataract, showed no excess (HR 0.934), arguing against gross differential healthcare contact between the arms. Agent-specific analyses showed no significant mortality signal for any individual JAKi (tofacitinib HR 1.052, baricitinib HR 1.213, and upadacitinib HR 1.090), and the only nominally significant agent-level finding was venous thromboembolism with baricitinib (HR 1.364) on 198 events. The narrowing of the signal to all-cause mortality alone, under a model that left MACE, heart failure, and VTE null, argues against a discrete atherothrombotic effect since identical adjustment did not attenuate all outcomes equally. Nonetheless, the corresponding E-value (1.586; 1.306 at the confidence limit) indicates that a confounder of only modest strength could account for the surviving association, so it is best read as a signal warranting surveillance rather than an established causal effect.

These findings extend and refine—rather than directly contradict—the cardiovascular safety concerns raised by ORAL Surveillance, the randomized non-inferiority trial that prompted the 2021 FDA boxed warning for JAKi in rheumatoid arthritis patients aged ≥50 years with at least one cardiovascular risk factor (9). In ORAL Surveillance, combined tofacitinib doses (5 and 10 mg twice daily) showed numerically higher MACE rates than TNFi (HR 1.33, 95% CI 0.91–1.94), with non-inferiority not demonstrated; venous thromboembolism risk was dose-dependent, reaching significance only at the 10-mg twice-daily dose—no longer approved for rheumatoid arthritis (9). Our real-world findings align with this signal only in part: the persistent all-cause mortality excess is directionally consistent with the broader JAKi safety profile in ORAL Surveillance and with subsequent TriNetX-based analyses by Shih et al. (22) and Huang et al. (23), both reporting higher mortality with JAKi than TNFi in RA. However, our two-component MACE result (nonfatal MI or ischemic stroke) was essentially null (HR 1.002), and heart failure hospitalization—elevated under an earlier specification of this cohort—no longer differed, in contrast to the three-component MACE signal in ORAL Surveillance. This divergence has several methodological and definitional explanations.

Two factors likely explain why our primary two-component MACE result diverges from ORAL Surveillance and earlier real-world signals. First, endpoint definition: ORAL Surveillance operationalizes MACE as a three-component composite including cardiovascular death, whereas our primary endpoint was restricted to nonfatal MI and ischemic stroke because cardiovascular-specific mortality cannot be reliably ascertained from TriNetX. Substituting all-cause mortality into a three-component composite moved the estimate upward but not to significance (HR 1.058, 95% CI 0.992–1.127); because such a composite captures non-cardiovascular deaths—particularly from infection and malignancy, both independently affected by JAKi (9, 21)—it represents an upper bound on the cardiovascular signal rather than a like-for-like replication of the adjudicated ORAL composite (9). That even this upper bound fails to reach significance leaves the cardiovascular contribution in doubt. Second, confounder specification: Relative to an earlier version of this analysis reported as a preprint (24), the estimates reported here were derived under a propensity-score model extended in two documented stages—first by adding individual csDMARDs, oral corticosteroids and baseline erythrocyte sedimentation rate, and subsequently by adding prior non-TNF biologic exposure and C-reactive protein. Across these stages the MACE association attenuated to the null, heart failure hospitalization fell from 1.111 to 1.019 and the three-component composite from 1.084 to 1.058, both losing significance, while all-cause mortality fell from 1.205 to 1.158 and persisted (**Supplementary Table S20**). The preprint analysis differed from these in cohort derivation and in the handling of prevalent events as well as in covariate specification, so it is not directly comparable and is not tabulated; the successive attenuation across the two comparable specifications is nonetheless consistent with severity proxies embedded within the model rather than applied *post hoc*, removing a substantial part of the apparent cardiovascular signal. The most parsimonious interpretation is that the JAKi safety signal is confined to all-cause mortality, encompassing both cardiovascular and non-cardiovascular mechanisms rather than a discrete excess in nonfatal MI or stroke. Sensitivity to severity proxies supports channeling bias as a partial explanation, though a true treatment-associated component cannot be excluded.

Agent-specific analyses revealed heterogeneity in point estimates that should nonetheless be read cautiously since no agent reached significance for either MACE or all-cause mortality. Upadacitinib, a preferentially JAK1-selective inhibitor, showed no significant excess in either MACE (HR 0.870, 95% CI 0.753–1.006) or all-cause mortality (HR 1.090, 95% CI 0.912–1.303), with a numerically lower MACE point estimate that is consistent with long-term follow-up of the SELECT program (25). This pattern is compatible with the hypothesis that greater JAK1 selectivity may reduce off-target effects on hematopoiesis, lipid metabolism, and platelet function, although mechanistic causality cannot be inferred from observational data, and the upadacitinib cohort is concentrated in the post-2021 era, so calendar time effects cannot be separated from any agent effect (20). Tofacitinib, with predominant JAK1/JAK3 activity, showed no significant excess in either MACE (HR 1.038, 95% CI 0.948–1.138) or all-cause mortality (HR 1.052, 95% CI 0.954–1.160); the mortality signal observed in this cohort under an earlier model specification did not survive improved adjustment, and its only remnant was a nominally significant pre-2021 estimate (HR 1.123, 95% CI 1.004–1.256) partially concordant with the mortality and broader safety signals reported in ORAL Surveillance (9). Baricitinib retained the largest mortality point estimate (HR 1.213, 95% CI 0.939–1.565) without reaching significance and was the sole agent with a nominally significant finding of any kind—venous thromboembolism (HR 1.364, 95% CI 1.028–1.809)—which should be interpreted with caution given the smaller matched sample (*n* = 1,720 per group), only 198 events, the wider confidence interval, and the likelihood that channeling bias is more pronounced for an agent often prescribed to refractory patients. Notably, the baricitinib mortality estimate was the least stable in this analysis, its excess hazard falling by more than two-fifths (1.370 to 1.213) as severity proxies entered the propensity score; this instability, together with the residual imbalance in baseline C-reactive protein in this cohort, indicates that early estimates for this agent were substantially inflated by channeling toward more refractory patients. Mechanistically, JAK2 inhibition may affect erythropoiesis and thrombotic pathways, providing biological plausibility for excess thrombotic events, although confirmation in head-to-head studies is needed (20).

Across all outcomes, the modest excess in all-cause mortality with JAKi initiation (HR 1.158, 95% CI 1.058–1.268; absolute excess 10.4 per 1,000 over 5 years) is the only association that survived improved adjustment and is therefore the most clinically relevant finding. Prior real-world evidence for a JAKi mortality signal is mixed. Two TriNetX-based analyses reported higher mortality with JAKi than with TNFi in RA (22, 23), although these share our data source and cannot be considered fully independent. The US Medicare- and claims-based STAR-RA study (Khosrow-Khavar et al.) reported an all-cause mortality HR of 1.20 (95% CI 0.98–1.46) for tofacitinib versus TNFi—a point estimate close to our pooled class estimate, though likewise not reaching statistical significance (14). In contrast, the US Corrona registry analysis (Kremer et al.) found no excess mortality with tofacitinib versus biologic DMARDs (adjusted HR 0.91, 95% CI 0.59–1.42) (26), a null result that our own tofacitinib-specific estimate (HR 1.052, 95% CI 0.954–1.160) now more closely resembles. Corrona captures prospectively recorded disease activity (CDAI/DAS28) and seropositivity that TriNetX does not, and the divergence is consistent with residual channeling bias by disease severity in EHR data. The persistence of the pooled mortality signal after improved adjustment for csDMARDs and corticosteroids suggests a possible treatment-associated component, but its confinement to the pooled analysis—absent in every agent-specific cohort—is equally compatible with residual channeling bias toward sicker patients receiving JAKi. Mechanistically, JAK-STAT signaling is central to host defense and tumor surveillance, and excess malignancy and serious infection signals observed in ORAL Surveillance and registry data provide biological plausibility for non-cardiovascular contributions (9, 20, 21). Continued pharmacovigilance for infection and malignancy is warranted in JAKi-treated patients.

Subgroup analyses showed no consistent effect modification by sex or baseline cardiovascular risk burden after improved propensity score matching. Sex-stratified MACE did not differ in either sex, and although all-cause mortality reached nominal significance among women (HR 1.123, 95% CI 1.013–1.244), the male estimate was of similar magnitude and direction without reaching significance (HR 1.161, 95% CI 0.986–1.368), indicating a shared class-level effect rather than true sex-specific modification. Among ASCVD-free patients, no MACE or mortality signal reached significance in any risk-burden stratum; absolute risks rose with cardiovascular burden as expected, but the MACE hazard ratios were near-identical across strata (1.126, 1.139, and 1.119) and the mortality estimates showed no gradient. In contrast to *post-hoc* ORAL Surveillance signals suggesting risk concentration in patients with established ASCVD or high cardiovascular burden (10), our analysis does not support sex or baseline cardiovascular risk burden as a basis for differential JAKi prescribing. Index-year stratification likewise showed no difference in either era, and the nominally significant agent-by-era estimates—baricitinib pre-2021 mortality (HR 2.271) and the apparently protective upadacitinib post-2021 estimates (MACE HR 0.825, mortality HR 0.815)—arise from cohorts in which agent and calendar time are inseparable, so they are era-confounded and hypothesis-generating rather than evidence of differential agent safety.

This study has several strengths. It analyzed a large real-world cohort of RA patients aged ≥50 years with ≥1 cardiovascular risk factor—the population in whom JAKi safety concerns are most relevant—using a target trial emulation with a new-user, active-comparator design and a 90-day landmark that minimizes immortal-time bias and early-event influence. The improved propensity-score model incorporated individual csDMARDs, oral corticosteroids, prior non-TNF biologic exposure, and baseline C-reactive protein and ESR—severity markers that independently affect cardiovascular and metabolic risk; to our knowledge, this is the largest real-world JAKi safety analysis to embed treatment-history and inflammatory severity proxies within the model itself rather than as post-matching adjustments, and the resulting attenuation of the apparent class-level MACE and heart failure signals is its principal methodological contribution. The inclusion of cataract as a prespecified negative-control outcome, which showed no excess with JAKi (HR 0.934, 95% CI 0.867–1.007), provides direct evidence against gross residual confounding by differential healthcare contact, although only for diagnosis-coded outcomes and not for mortality, which is ascertained separately. The proportional hazards assumption, tested by the platform’s proportionality test, held for all primary and secondary outcomes in the main matched cohort, with non-proportionality confined to selected upadacitinib and era-stratified comparisons, whose hazard ratios are interpreted as time-averaged. Finally, reporting both relative and absolute estimates supports clinical interpretation, and separately matched agent-specific, subgroup, and index-year cohorts allowed the detection of heterogeneity that class-level analysis would conceal.

### Limitations

This study also has important limitations. First, despite improved adjustment, residual confounding remains likely, and the E-value for the surviving mortality association (1.586, falling to 1.306 at the confidence limit) indicates that an unmeasured confounder of only modest strength would suffice to explain it. Unmeasured covariates including disease activity scores, seropositivity status, smoking pack-years, body mass index, and lipid levels are not reliably captured in TriNetX. The inflammatory markers that were included were recorded for only approximately 60% of matched patients in each arm, so the propensity score could draw on a baseline inflammatory value for little more than half the cohort. Inclusion in the model did not guarantee balance: in the baricitinib cohorts, C-reactive protein remained substantially imbalanced after matching (SMD 0.283 pooled, 0.373 post-2021) despite entering the propensity score, showing that where the underlying severity difference was largest the adjustment failed. Adjustment for baseline disease activity is therefore better described as limited than as partial, and residual channeling by disease severity remains a live alternative explanation for the surviving mortality association rather than a remote one. Disease duration could likewise not be ascertained: the earliest recorded rheumatoid arthritis code marks entry into a contributing healthcare organization rather than true disease onset, so duration is left-truncated to an unknown and variable degree. Because disease duration determines both line of therapy and mortality risk, its absence contributes directly to the channeling that baseline biologic exposure captures only in part. Channeling bias may therefore persist, as JAKi are more often prescribed to patients with refractory or aggressive disease who may carry higher baseline mortality risk independent of treatment; consistent with this, prior abatacept, tocilizumab, and rituximab exposure remained modestly imbalanced in the same direction after matching.

Second, exposure was defined by recorded prescription orders and analyzed under an intention-to-treat framework without censoring for discontinuation or switching. Real-world persistence on biologic and targeted synthetic DMARDs over a 5-year horizon is limited (27, 28): a systematic review and meta-analysis of persistence with biological agents in rheumatoid arthritis reported 5-year retention of approximately 41%–66% depending on agent and line of therapy (27). A substantial proportion of our cohort would therefore be expected to switch or stop within the observation window. The platform does not record whether or when an index drug was discontinued, so treatment persistence could not be quantified and an as-treated analysis censoring at discontinuation or switch was not feasible. The resulting exposure misclassification is expected to be largely non-differential and therefore to attenuate true differences toward the null. It is most consequential for the agent-specific comparisons, in which patients who switch between JAK inhibitors contribute to more than one matched cohort.

Third, death is a competing event for the nonfatal MACE endpoint, and the platform supports neither subdistribution hazard models nor the patient-level data required to fit them. Two consequences follow. Cause-specific hazard ratios are estimated among patients still alive and event-free, so the greater mortality in the JAKi arm removes patients from the risk set before a nonfatal event can occur, which attenuates the MACE hazard ratio toward the null. Conversely, cumulative incidence derived as one minus the Kaplan–Meier survival function treats death as censoring and therefore overestimates incidence and does so more in the arm with more competing events. These two effects act in opposite directions, and both are bounded by the modest absolute mortality difference between arms (1.04 percentage points at 5 years), so neither is likely to alter the null MACE conclusion materially; the estimates should nonetheless be read as cause-specific rather than subdistribution quantities.

Fourth, outcome ascertainment relies on electronic health record coding and is subject to misclassification in two opposing directions. Care delivered outside a contributing healthcare organization is not observed, and for acute events such as myocardial infarction and stroke—which frequently present to the nearest available hospital rather than to the usual provider—this undercapture is likely to be appreciable and broadly non-differential, biasing comparisons toward the null. Against this, JAKi initiators prescribed under the 2021 boxed warning may undergo more intensive cardiovascular monitoring, which would differentially inflate event capture in the JAKi arm; the longer duration of available observation in the JAKi arm is consistent with greater healthcare contact. The prespecified cataract negative-control outcome bears directly on this question: a commonly coded diagnosis with no mechanistic link to either drug class showed no excess with JAKi (HR 0.934, 95% CI 0.867–1.007) and if anything favored JAKi, providing empirical evidence against surveillance-driven overcapture as the dominant mechanism. This evidence is specific to diagnosis-coded outcomes and does not extend to mortality. Cataract, like myocardial infarction and stroke, is ascertained from diagnosis codes generated at healthcare encounters, whereas all-cause mortality is ascertained from vital status recorded within the network; the two rely on different capture mechanisms, and a negative control of this kind tests only the former. Because availability of observation was longer in the JAKi arm (median 1,320 versus 1,111 days), a greater proportion of deaths occurring in that arm may fall within the observed record, which would inflate the mortality association while leaving cataract unaffected. We cannot exclude this with the data available, and the surviving mortality association should therefore be read against two unresolved alternatives—residual channeling and differential completeness of death ascertainment—as well as against its E-value. On balance, we therefore consider non-differential undercapture, which biases toward the null, the more likely net direction for the nonfatal outcomes. Because patients with the outcome recorded at baseline were retained, outcomes with high baseline prevalence—notably heart failure, recorded in 7.8% of JAKi and 8.0% of TNFi initiators—will include re-recording of pre-existing disease alongside new events. Baseline prevalence was closely balanced after matching (SMD <0.01), so the between-arm comparison is unaffected, but the absolute 5-year heart failure risks should be read as event rates rather than incidence.

Fifth, the platform constrains time-to-event reporting in three respects, which we report transparently rather than approximate. Person-time at risk cannot be derived from the available exports; the duration metric we report measures the availability of observation and is not equivalent to time at risk. Risk-set counts are not exported, so conventional numbers-at-risk cannot be tabulated beneath the survival curves. Median availability of observation was 1,320 days (JAKi) and 1,111 days (TNFi), well short of the 1,825-day window, so 5-year estimates rest on a diminishing risk set; crude and Kaplan–Meier values diverge accordingly (MACE 6.35% [1,191/18,757] versus 9.474% in the JAKi arm), which is why Kaplan–Meier estimates are reported throughout for consistency with the Cox models. These distributions could not be exported for any stratified cohort before access ended. Survival curves for the four principal outcomes are plotted as step functions from the platform’s day-level survival function with its confidence bounds from day 90 to day 1,825, so every plotted point is platform output and no value is interpolated; for the three-component composite and the negative-control outcome, only endpoint estimates were available and no curve is shown. Finally, the longer availability of observation in the JAKi arm (median 1,320 versus 1,111 days) raises the possibility of informative censoring, whereby loss of observation is related to both exposure and prognosis; this cannot be excluded in a federated network in which patients may move between contributing organizations.

Sixth, the pooled mortality signal was not reproduced in any agent-specific cohort, which may reflect either loss of power in the smaller separately matched cohorts or the pooled estimate being driven by residual confounding rather than a class effect; these possibilities cannot be distinguished in this design.

Seventh, the baricitinib analysis is based on a smaller matched sample (*n* = 1,720 per group versus 13,078 for tofacitinib and 7,927 for upadacitinib), yielding wider confidence intervals; the baricitinib venous thromboembolism estimate (HR 1.364, 198 events) and the pre-2021 baricitinib mortality estimate (HR 2.271, 85 events among 469 pairs) should be interpreted as hypothesis-generating and are far more sensitive to model specification than the other agent estimates. Residual confounding by inflammatory burden was greatest in the baricitinib cohorts, where baseline C-reactive protein remained imbalanced after matching (SMD 0.283 in the pooled agent cohort and 0.373 post-2021) in the direction that would inflate any apparent harm.

Eighth, agent-specific, subgroup, and index-year cohorts were matched separately, precluding formal interaction testing. Index date was not itself a matching variable, and because JAK inhibitors entered practice later, JAKi follow-up is displaced toward later calendar years; separately matched era cohorts address this only in part since a split at January 1, 2021 does not cleanly separate the 2020–2022 period of pandemic-related excess mortality, which straddles it. Calendar time therefore remains partially confounded with exposure, and finer stratification could not be extracted before network access ended. Both era-stratified mortality estimates were attenuated and non-significant (pre-2021 HR 1.079, 95% CI 0.971–1.199; post-2021 HR 1.061, 0.920–1.225), but both intervals include the pooled estimate, so calendar time contribution and reduced power cannot be distinguished. Agent uptake is likewise correlated with calendar time, so agent and era effects cannot be disentangled.

Finally, real-world comparative effect estimates remain sensitive to confounder specification, and our findings—like all observational analyses of JAKi cardiovascular safety—should be interpreted as estimates conditional on the variables available for adjustment.

## Conclusion

In this 5-year target trial emulation, JAKi initiation was associated with a modest excess in all-cause mortality compared with TNFi, while no significant excess was observed in MACE, heart failure hospitalization, or venous thromboembolism after improved propensity-score matching incorporating individual csDMARDs, corticosteroids, prior non-TNF biologic exposure, and C-reactive protein. No individual agent carried a significant mortality signal, and the only nominally significant agent-level finding was venous thromboembolism with baricitinib in a small matched cohort. The confinement of the surviving signal to all-cause mortality, together with an E-value of 1.586, indicates that the null MACE result should be read neither as class-wide cardiovascular safety reassurance nor as confirmation of an atherothrombotic hazard. These findings refine rather than overturn the ORAL Surveillance signal (9). They do not support class-wide avoidance of JAK inhibitors, nor do they establish differential safety between agents, and continued pharmacovigilance for cardiovascular and non-cardiovascular mortality remains warranted.

## Data Availability

The datasets analyzed are the property of TriNetX LLC and were accessed under a license agreement. They are not publicly available and cannot be deposited or redistributed by the authors. The platform returns aggregate results only; no patient-level data were available to the investigators at any stage. Aggregate results may be accessed through the TriNetX platform by qualified researchers, subject to institutional agreements and platform governance policies. The authors’ access to the platform has since ended. All aggregate results underlying the conclusions of this study are reported in the article and **Supplementary Material**. The aggregate, day-level survival estimates underlying the Kaplan-Meier figures, and the R script used to generate the figures from them, are available from the corresponding author on reasonable request. No patient-level data are held by the authors and none can be shared. Requests to access these datasets should be directed to Laith Alamlih, layth.amleh@staff.alquds.edu — enquiries will be directed to TriNetX LLC (https://trinetx.com/contact/), which holds the data.
