# Supplementary material for: Cardiovascular and mortality outcomes with JAK versus TNF inhibitors in rheumatoid arthritis: a target trial emulation (CARDINAL-RA)
Source: Front Immunol. 2026 Sep 30;17:1908442. doi: 10.3389/fimmu.2026.1908442 (PMC13662084; doi:10.3389/fimmu.2026.1908442)
Supplement: Supplementary file 1 [file SupplementaryFile1.docx]

**Supplementary Figure S1. Kaplan–Meier cumulative incidence of each outcome in the main improved propensity score–matched cohort (n = 18,757 pairs).**

Panels show major adverse cardiovascular events, all-cause mortality, heart failure hospitalisation and venous thromboembolism in JAKi versus TNFi initiators. Curves are plotted as step functions from the platform's day-level survival function with shaded 95% confidence bands across the interval from day 90 (the landmark) to day 1,825; every plotted point is platform output and no value is interpolated. Risk-set counts are not exported by the platform, so numbers at risk cannot be tabulated beneath the curves. Vertical scales differ between panels to accommodate the different absolute event rates, so curve heights should not be compared across panels.

**Supplementary Figure S2. Kaplan–Meier cumulative incidence by individual JAK inhibitor.**

Curves are arranged by agent (tofacitinib, n = 13,078 per group; baricitinib, n = 1,720 per group; upadacitinib, n = 7,927 per group) and by outcome, each against its own separately matched TNFi comparator. Plotting conventions and the day 90 to day 1,825 interval are as for **Supplementary Figure S1**; vertical scales differ between agents to accommodate the higher absolute event rates in the baricitinib cohort, so curve heights should not be compared across rows.

**Supplementary Figure S3. Forest plot of subgroup analyses in separately matched cohorts.**

Hazard ratios with 95% confidence intervals in each separately matched stratum. For the sex strata (women, n = 15,551 per group; men, n = 3,983 per group) and the ASCVD-free cardiovascular risk strata (1 risk factor, n = 5,880; 2 risk factors, n = 4,550; ≥3 risk factors, n = 2,508 per group), MACE, all-cause mortality and the 3-component MACE composite are shown. For the index-era strata (pre-2021, n = 8,437; post-2021, n = 10,230 per group), heart failure hospitalisation and venous thromboembolism are shown in addition, giving five outcomes per era. Each stratum was matched separately, so formal interaction testing was not performed and results are interpreted by effect-size pattern rather than by tests of heterogeneity.

**Supplementary Figure S4. Forest plot of agent-by-index-year estimates.**

Hazard ratios with 95% confidence intervals for each JAK inhibitor within the pre-2021 and post-2021 index-era strata. Because agent uptake is strongly correlated with calendar time, agent and era effects cannot be separated in these cohorts; the nominally significant estimates are era-confounded and hypothesis-generating rather than evidence of differential agent safety.

**Supplementary Figure S5. Forest plot of agent-specific estimates across all outcomes.**

Hazard ratios with 95% confidence intervals for MACE, all-cause mortality, heart failure hospitalisation, venous thromboembolism and the 3-component MACE composite for each JAK inhibitor against its separately matched TNFi comparator. Open markers denote comparisons in which the proportional hazards assumption was violated on the platform's proportionality test — upadacitinib MACE (p = 0.037), upadacitinib venous thromboembolism (p < 0.0001) and the upadacitinib 3-component composite (p = 0.011) — whose hazard ratios are interpreted as time-averaged.

**CARDINAL-RA — Supplementary baseline characteristics tables**

**Supplementary Table S1. Baseline characteristics in the tofacitinib versus TNFi propensity score–matched cohort**

| **Characteristic** | **Before: Tofacitinib (n = 13,125)** | **Before: TNFi (n = 56,175)** | **SMD** | **After: Tofacitinib (n = 13,078)** | **After: TNFi (n = 13,078)** | **SMD** |
| --- | --- | --- | --- | --- | --- | --- |
| **Demographics** |  |  |  |  |  |  |
| Age, current, years (mean ± SD) | 68.1 ± 9.23 | 68.1 ± 9.55 | 0.0095 | 68.1 ± 9.22 | 68.1 ± 9.54 | 0.0009 |
| Age at index, years (mean ± SD) | 61.8 ± 9.18 | 61.6 ± 9.50 | 0.0297 | 61.8 ± 9.17 | 62.0 ± 9.47 | 0.0191 |
| Female, % | 80.02 | 75.33 | 0.1130 | 79.99 | 80.86 | 0.0220 |
| Male, % | 19.97 | 24.67 | 0.1131 | 20.00 | 19.14 | 0.0218 |
| Unknown gender, % | 0.08 | 0.02 | 0.0269 | 0.08 | 0 | 0.0391 |
| White, % | 67.25 | 69.75 | 0.0538 | 67.28 | 66.87 | 0.0088 |
| Black or African American, % | 14.32 | 14.57 | 0.0073 | 14.30 | 13.93 | 0.0105 |
| Asian, % | 4.19 | 2.92 | 0.0685 | 4.19 | 4.63 | 0.0212 |
| American Indian or Alaska Native, % | 0.65 | 0.84 | 0.0220 | 0.65 | 0.67 | 0.0028 |
| Native Hawaiian or Other Pacific Islander, % | 0.24 | 0.28 | 0.0089 | 0.24 | 0.34 | 0.0186 |
| Other race, % | 2.63 | 3.13 | 0.0298 | 2.62 | 2.64 | 0.0014 |
| Unknown race, % | 10.73 | 8.51 | 0.0754 | 10.73 | 10.93 | 0.0064 |
| Hispanic or Latino, % | 6.77 | 7.81 | 0.0397 | 6.74 | 6.15 | 0.0243 |
| Not Hispanic or Latino, % | 67.80 | 67.41 | 0.0084 | 67.84 | 68.33 | 0.0105 |
| Unknown ethnicity, % | 25.43 | 24.79 | 0.0147 | 25.42 | 25.52 | 0.0025 |
| **Cardiovascular risk factors and comorbidities** |  |  |  |  |  |  |
| Hypertension, % | 65.42 | 64.61 | 0.0170 | 65.41 | 65.97 | 0.0119 |
| Dyslipidemia, % | 54.50 | 51.45 | 0.0611 | 54.46 | 54.63 | 0.0035 |
| Overweight and obesity, % | 28.54 | 27.66 | 0.0195 | 28.46 | 28.12 | 0.0075 |
| Type 2 diabetes, % | 24.15 | 23.61 | 0.0127 | 24.09 | 24.19 | 0.0023 |
| Nicotine dependence, % | 17.16 | 18.17 | 0.0265 | 17.17 | 16.40 | 0.0207 |
| Tobacco use, % | 6.02 | 6.00 | 0.0009 | 6.03 | 6.09 | 0.0026 |
| Ischaemic heart disease, % | 16.27 | 16.41 | 0.0038 | 16.27 | 16.19 | 0.0023 |
| Cerebral infarction, % | 3.28 | 3.46 | 0.0101 | 3.27 | 3.10 | 0.0100 |
| Heart failure, % | 7.50 | 5.59 | 0.0773 | 7.49 | 7.91 | 0.0161 |
| Atrial fibrillation, % | 6.10 | 5.77 | 0.0136 | 6.10 | 5.90 | 0.0084 |
| Chronic kidney disease, % | 8.15 | 7.74 | 0.0148 | 8.13 | 8.30 | 0.0061 |
| Acute kidney injury, % | 6.11 | 5.19 | 0.0398 | 6.09 | 6.45 | 0.0148 |
| **Other autoimmune and inflammatory diagnoses** |  |  |  |  |  |  |
| Systemic lupus erythematosus, % | 4.95 | 3.38 | 0.0786 | 4.92 | 4.74 | 0.0086 |
| Systemic sclerosis, % | 1.12 | 0.66 | 0.0489 | 1.11 | 1.14 | 0.0029 |
| Dermatomyositis, % | 1.01 | 0.38 | 0.0759 | 0.95 | 0.98 | 0.0031 |
| Antiphospholipid syndrome, % | 0.63 | 0.54 | 0.0122 | 0.62 | 0.63 | 0.0010 |
| Arthropathic psoriasis, % | 0.38 | 0.27 | 0.0194 | 0.38 | 0.29 | 0.0158 |
| Ankylosing spondylitis, % | 0.30 | 0.17 | 0.0270 | 0.30 | 0.27 | 0.0058 |
| Crohn's disease, % | 0.18 | 0.14 | 0.0097 | 0.18 | 0.18 | 0.0018 |
| Ulcerative colitis, % | 0.11 | 0.12 | 0.0048 | 0.11 | 0.18 | 0.0201 |
| **csDMARDs and corticosteroids** |  |  |  |  |  |  |
| Methotrexate, % | 51.95 | 51.02 | 0.0185 | 51.92 | 51.35 | 0.0115 |
| Hydroxychloroquine, % | 36.40 | 30.75 | 0.1198 | 36.37 | 37.01 | 0.0133 |
| Leflunomide, % | 23.97 | 15.79 | 0.2061 | 23.83 | 24.55 | 0.0168 |
| Sulfasalazine, % | 16.95 | 13.02 | 0.1105 | 16.95 | 17.14 | 0.0049 |
| Prednisone, % | 62.15 | 55.10 | 0.1435 | 62.05 | 63.02 | 0.0201 |
| Methylprednisolone, % | 42.37 | 33.90 | 0.1751 | 42.22 | 42.66 | 0.0088 |
| Dexamethasone, % | 25.50 | 22.20 | 0.0776 | 25.46 | 25.66 | 0.0046 |
| **Prior non-TNF biologic DMARDs** |  |  |  |  |  |  |
| Abatacept, % | 12.16 | 3.44 | 0.3298 | 11.92 | 11.59 | 0.0102 |
| Tocilizumab, % | 7.86 | 1.92 | 0.2781 | 7.59 | 6.70 | 0.0347 |
| Rituximab, % | 3.86 | 1.03 | 0.1837 | 3.75 | 3.30 | 0.0245 |
| **Laboratory parameters** |  |  |  |  |  |  |
| C-reactive protein, mg/L (mean ± SD) | 15.3 ± 29.0 | 16.1 ± 31.0 | 0.0238 | 15.4 ± 29.0 | 15.8 ± 29.4 | 0.0155 |
| C-reactive protein, mg/L (median [IQR]) | 6.2 [12.9] | 6.6 [12.9] | — | 6.2 [12.9] | 6.5 [13.3] | — |
| C-reactive protein recorded, % | 60.66 | 55.10 | 0.1128 | 60.54 | 61.38 | 0.0171 |
| Erythrocyte sedimentation rate, mm/h (mean ± SD) | 28.3 ± 24.8 | 27.4 ± 24.2 | 0.0336 | 28.3 ± 24.8 | 27.9 ± 24.7 | 0.0128 |
| Erythrocyte sedimentation rate, mm/h (median [IQR]) | 21 [30] | 20 [28] | — | 21 [30] | 21 [29] | — |
| Erythrocyte sedimentation rate recorded, % | 60.31 | 56.16 | 0.0842 | 60.21 | 60.76 | 0.0113 |

Baseline characteristics of patients initiating tofacitinib versus tumour necrosis factor inhibitors (TNFi), before and after 1:1 propensity score matching. The propensity score model included demographics, cardiovascular risk factors and comorbidities, other autoimmune and inflammatory diagnoses, conventional synthetic DMARDs, corticosteroids, prior non-TNF biologic DMARDs, and baseline C-reactive protein and erythrocyte sedimentation rate. Values are mean ± standard deviation (SD) or median [interquartile range] for continuous variables and percentages for categorical variables. Standardised mean differences (SMD) < 0.1 indicate adequate balance. After matching all SMDs were < 0.04, indicating good covariate balance; the largest residual differences were for unknown gender (0.039), tocilizumab (0.035), rituximab (0.025) and Hispanic or Latino ethnicity (0.024). The unknown-gender difference reflects a cell of 10 versus 0 patients and is not clinically meaningful. C-reactive protein and erythrocyte sedimentation rate values were recorded in approximately 60% of patients; the proportion with a recorded value differed between groups before matching (SMD 0.113 for C-reactive protein) but was balanced after matching (SMD 0.017). Interquartile range is reported as the width of the interquartile range. JAKi = Janus kinase inhibitors; TNFi = tumour necrosis factor inhibitors; SD = standard deviation; SMD = standardised mean difference.

**Supplementary Table S2. Baseline characteristics in the baricitinib versus TNFi propensity score–matched cohort**

| **Characteristic** | **Before: Baricitinib (n = 1,729)** | **Before: TNFi (n = 57,077)** | **SMD** | **After: Baricitinib (n = 1,720)** | **After: TNFi (n = 1,720)** | **SMD** |
| --- | --- | --- | --- | --- | --- | --- |
| **Demographics** |  |  |  |  |  |  |
| Age, current, years (mean ± SD) | 68.7 ± 9.49 | 68.1 ± 9.56 | 0.0658 | 68.7 ± 9.50 | 68.9 ± 9.47 | 0.0208 |
| Age at index, years (mean ± SD) | 64.2 ± 9.62 | 61.5 ± 9.50 | 0.2778 | 64.2 ± 9.63 | 64.5 ± 9.52 | 0.0281 |
| Female, % | 76.40 | 75.37 | 0.0242 | 76.34 | 78.49 | 0.0515 |
| Male, % | 23.60 | 24.63 | 0.0241 | 23.66 | 21.51 | 0.0515 |
| Unknown gender, % | 0 | 0.02 | 0.0187 | 0 | 0 | — |
| White, % | 59.92 | 69.64 | 0.2045 | 60.17 | 59.42 | 0.0154 |
| Black or African American, % | 9.72 | 14.87 | 0.1574 | 9.71 | 8.08 | 0.0572 |
| Asian, % | 4.57 | 2.89 | 0.0888 | 4.54 | 4.77 | 0.0110 |
| American Indian or Alaska Native, % | 0.93 | 0.83 | 0.0100 | 0.93 | 0.93 | < 0.0001 |
| Native Hawaiian or Other Pacific Islander, % | 0.58 | 0.28 | 0.0462 | 0.58 | 0.58 | < 0.0001 |
| Other race, % | 2.43 | 3.11 | 0.0417 | 2.44 | 3.08 | 0.0390 |
| Unknown race, % | 22.09 | 8.38 | 0.3886 | 21.86 | 23.37 | 0.0361 |
| Hispanic or Latino, % | 6.54 | 7.73 | 0.0463 | 6.57 | 7.09 | 0.0207 |
| Not Hispanic or Latino, % | 54.37 | 67.86 | 0.2796 | 54.48 | 53.14 | 0.0268 |
| Unknown ethnicity, % | 39.10 | 24.41 | 0.3195 | 38.95 | 39.77 | 0.0167 |
| **Cardiovascular risk factors and comorbidities** |  |  |  |  |  |  |
| Hypertension, % | 69.00 | 64.67 | 0.0920 | 69.01 | 69.07 | 0.0013 |
| Dyslipidemia, % | 58.70 | 51.58 | 0.1436 | 58.78 | 59.19 | 0.0083 |
| Overweight and obesity, % | 36.73 | 27.58 | 0.1967 | 36.57 | 37.04 | 0.0096 |
| Type 2 diabetes, % | 32.45 | 23.64 | 0.1970 | 32.33 | 32.62 | 0.0062 |
| Nicotine dependence, % | 19.38 | 18.11 | 0.0325 | 19.30 | 18.72 | 0.0148 |
| Tobacco use, % | 5.73 | 6.01 | 0.0121 | 5.76 | 5.58 | 0.0075 |
| Ischaemic heart disease, % | 27.36 | 16.41 | 0.2671 | 27.21 | 27.50 | 0.0065 |
| Cerebral infarction, % | 5.55 | 3.50 | 0.0991 | 5.58 | 4.54 | 0.0478 |
| Heart failure, % | 15.56 | 5.61 | 0.3278 | 15.41 | 15.64 | 0.0064 |
| Atrial fibrillation, % | 12.78 | 5.78 | 0.2432 | 12.67 | 12.04 | 0.0194 |
| Chronic kidney disease, % | 17.53 | 7.75 | 0.2975 | 17.44 | 16.74 | 0.0185 |
| Acute kidney injury, % | 19.55 | 5.22 | 0.4458 | 19.42 | 18.49 | 0.0237 |
| **Other autoimmune and inflammatory diagnoses** |  |  |  |  |  |  |
| Systemic lupus erythematosus, % | 6.71 | 3.40 | 0.1513 | 6.63 | 6.34 | 0.0118 |
| Systemic sclerosis, % | 0.98 | 0.67 | 0.0345 | 0.93 | 0.99 | 0.0060 |
| Dermatomyositis, % | 1.16 | 0.38 | 0.0888 | 1.11 | 0.99 | 0.0114 |
| Antiphospholipid syndrome, % | 0.75 | 0.54 | 0.0267 | 0.70 | 0.76 | 0.0068 |
| Arthropathic psoriasis, % | 0.58 | 0.27 | 0.0475 | 0.58 | 0.58 | < 0.0001 |
| Ankylosing spondylitis, % | 0.58 | 0.17 | 0.0677 | 0.58 | 0.58 | < 0.0001 |
| Crohn's disease, % | 0.58 | 0.14 | 0.0744 | 0.58 | 0.58 | < 0.0001 |
| Ulcerative colitis, % | 0.58 | 0.12 | 0.0776 | 0.58 | 0.58 | < 0.0001 |
| **csDMARDs and corticosteroids** |  |  |  |  |  |  |
| Methotrexate, % | 37.65 | 50.41 | 0.2592 | 37.67 | 35.76 | 0.0398 |
| Hydroxychloroquine, % | 33.37 | 30.32 | 0.0655 | 33.37 | 32.91 | 0.0099 |
| Leflunomide, % | 20.42 | 15.56 | 0.1266 | 20.35 | 19.30 | 0.0263 |
| Sulfasalazine, % | 16.37 | 12.86 | 0.0994 | 16.34 | 15.64 | 0.0190 |
| Prednisone, % | 48.35 | 54.48 | 0.1229 | 48.43 | 45.12 | 0.0665 |
| Methylprednisolone, % | 43.55 | 33.88 | 0.1996 | 43.55 | 42.56 | 0.0200 |
| Dexamethasone, % | 41.76 | 22.26 | 0.4274 | 41.74 | 40.52 | 0.0248 |
| **Prior non-TNF biologic DMARDs** |  |  |  |  |  |  |
| Abatacept, % | 12.20 | 3.42 | 0.3319 | 11.98 | 11.22 | 0.0236 |
| Tocilizumab, % | 12.32 | 1.91 | 0.4136 | 11.98 | 10.76 | 0.0385 |
| Rituximab, % | 7.29 | 1.03 | 0.3177 | 6.86 | 5.70 | 0.0479 |
| **Laboratory parameters** |  |  |  |  |  |  |
| C-reactive protein, mg/L (mean ± SD) | 33.3 ± 54.8 | 16.0 ± 31.0 | 0.3872 | 33.3 ± 54.9 | 19.8 ± 39.1 | 0.2831 |
| C-reactive protein, mg/L (median [IQR]) | 9.6 [32.0] | 6.6 [12.9] | — | 9.6 [32.0] | 6.7 [14.9] | — |
| C-reactive protein recorded, % | 63.85 | 55.04 | 0.1801 | 63.66 | 60.64 | 0.0624 |
| Erythrocyte sedimentation rate, mm/h (mean ± SD) | 29.8 ± 25.7 | 27.4 ± 24.2 | 0.0941 | 29.8 ± 25.7 | 29.3 ± 26.2 | 0.0183 |
| Erythrocyte sedimentation rate, mm/h (median [IQR]) | 22 [33] | 20 [28] | — | 22 [33] | 21 [31] | — |
| Erythrocyte sedimentation rate recorded, % | 48.18 | 56.54 | 0.1679 | 48.26 | 45.47 | 0.0559 |

Baseline characteristics of patients initiating baricitinib versus tumour necrosis factor inhibitors (TNFi), before and after 1:1 propensity score matching. The propensity score model included demographics, cardiovascular risk factors and comorbidities, other autoimmune and inflammatory diagnoses, conventional synthetic DMARDs, corticosteroids, prior non-TNF biologic DMARDs, and baseline inflammatory markers. Values are mean ± standard deviation (SD) or median [interquartile range] for continuous variables and percentages for categorical variables. Standardised mean differences (SMD) < 0.1 indicate adequate balance. Baricitinib initiators differed substantially from unmatched TNFi initiators across cardiovascular and renal comorbidity, corticosteroid exposure and prior non-TNF biologic use, and these differences resolved after matching: type 2 diabetes (0.006), heart failure (0.006), ischaemic heart disease (0.007), chronic kidney disease (0.019), abatacept (0.024) and tocilizumab (0.039). Among categorical covariates the largest residual differences after matching were for prednisone (0.067), Black or African American race (0.057), sex (0.052), rituximab (0.048) and cerebral infarction (0.048). Baseline C-reactive protein remained imbalanced after matching (33.3 ± 54.9 versus 19.8 ± 39.1 mg/L; SMD 0.283), indicating that baricitinib initiators carried a systematically higher inflammatory burden than any available matched TNFi comparator; erythrocyte sedimentation rate was balanced (SMD 0.018). C-reactive protein was recorded in approximately 61–64% of patients and erythrocyte sedimentation rate in 45–48%; the proportion with a recorded value was balanced after matching for both markers. Interquartile range is reported as the width of the interquartile range. TNFi = tumour necrosis factor inhibitors; SD = standard deviation; SMD = standardised mean difference.

**Supplementary Table S3. Baseline characteristics in the upadacitinib versus TNFi propensity score–matched cohort**

| **Characteristic** | **Before: Upadacitinib (n = 8,192)** | **Before: TNFi (n = 57,077)** | **SMD** | **After: Upadacitinib (n = 7,927)** | **After: TNFi (n = 7,927)** | **SMD** |
| --- | --- | --- | --- | --- | --- | --- |
| **Demographics** |  |  |  |  |  |  |
| Age, current, years (mean ± SD) | 65.4 ± 8.76 | 68.1 ± 9.56 | 0.2948 | 65.5 ± 8.76 | 65.3 ± 9.10 | 0.0143 |
| Age at index, years (mean ± SD) | 62.3 ± 8.76 | 61.5 ± 9.50 | 0.0870 | 62.3 ± 8.77 | 62.5 ± 9.17 | 0.0150 |
| Female, % | 80.86 | 75.37 | 0.1331 | 80.56 | 80.13 | 0.0108 |
| Male, % | 19.12 | 24.63 | 0.1336 | 19.42 | 19.86 | 0.0111 |
| Unknown gender, % | 0.12 | 0.02 | 0.0396 | 0.13 | 0.13 | < 0.0001 |
| White, % | 69.60 | 69.64 | 0.0007 | 69.56 | 69.36 | 0.0044 |
| Black or African American, % | 13.77 | 14.87 | 0.0314 | 13.81 | 13.99 | 0.0051 |
| Asian, % | 3.77 | 2.89 | 0.0492 | 3.70 | 3.76 | 0.0033 |
| American Indian or Alaska Native, % | 0.72 | 0.83 | 0.0128 | 0.73 | 0.63 | 0.0123 |
| Native Hawaiian or Other Pacific Islander, % | 0.33 | 0.28 | 0.0096 | 0.33 | 0.29 | 0.0068 |
| Other race, % | 2.11 | 3.11 | 0.0628 | 2.12 | 2.07 | 0.0035 |
| Unknown race, % | 9.69 | 8.38 | 0.0457 | 9.75 | 9.90 | 0.0051 |
| Hispanic or Latino, % | 8.51 | 7.73 | 0.0286 | 8.50 | 8.50 | < 0.0001 |
| Not Hispanic or Latino, % | 68.34 | 67.86 | 0.0101 | 68.16 | 68.16 | < 0.0001 |
| Unknown ethnicity, % | 23.16 | 24.41 | 0.0294 | 23.34 | 23.34 | < 0.0001 |
| **Cardiovascular risk factors and comorbidities** |  |  |  |  |  |  |
| Hypertension, % | 66.41 | 64.67 | 0.0365 | 66.29 | 65.61 | 0.0144 |
| Dyslipidemia, % | 61.22 | 51.58 | 0.1953 | 60.97 | 60.54 | 0.0088 |
| Overweight and obesity, % | 35.24 | 27.58 | 0.1655 | 35.15 | 35.47 | 0.0069 |
| Type 2 diabetes, % | 24.61 | 23.64 | 0.0226 | 24.59 | 24.88 | 0.0067 |
| Nicotine dependence, % | 17.53 | 18.11 | 0.0151 | 17.62 | 17.79 | 0.0043 |
| Tobacco use, % | 6.86 | 6.01 | 0.0347 | 6.88 | 7.12 | 0.0094 |
| Ischaemic heart disease, % | 17.62 | 16.41 | 0.0320 | 17.52 | 17.33 | 0.0050 |
| Cerebral infarction, % | 3.31 | 3.50 | 0.0103 | 3.36 | 3.61 | 0.0138 |
| Heart failure, % | 7.28 | 5.61 | 0.0680 | 7.20 | 7.22 | 0.0005 |
| Atrial fibrillation, % | 5.87 | 5.78 | 0.0041 | 5.90 | 5.80 | 0.0043 |
| Chronic kidney disease, % | 10.46 | 7.75 | 0.0944 | 10.46 | 10.55 | 0.0029 |
| Acute kidney injury, % | 7.12 | 5.22 | 0.0791 | 7.09 | 7.27 | 0.0068 |
| **Other autoimmune and inflammatory diagnoses** |  |  |  |  |  |  |
| Systemic lupus erythematosus, % | 5.71 | 3.40 | 0.1109 | 5.41 | 5.16 | 0.0113 |
| Systemic sclerosis, % | 1.16 | 0.67 | 0.0513 | 1.07 | 1.15 | 0.0072 |
| Dermatomyositis, % | 1.07 | 0.38 | 0.0815 | 0.96 | 0.93 | 0.0026 |
| Antiphospholipid syndrome, % | 0.44 | 0.54 | 0.0141 | 0.40 | 0.44 | 0.0058 |
| Arthropathic psoriasis, % | 0.65 | 0.27 | 0.0559 | 0.59 | 0.48 | 0.0155 |
| Ankylosing spondylitis, % | 0.68 | 0.17 | 0.0796 | 0.49 | 0.42 | 0.0113 |
| Crohn's disease, % | 0.16 | 0.14 | 0.0062 | 0.15 | 0.20 | 0.0120 |
| Ulcerative colitis, % | 0.17 | 0.12 | 0.0131 | 0.16 | 0.16 | < 0.0001 |
| **csDMARDs and corticosteroids** |  |  |  |  |  |  |
| Methotrexate, % | 53.64 | 50.41 | 0.0646 | 53.31 | 52.87 | 0.0088 |
| Hydroxychloroquine, % | 39.39 | 30.32 | 0.1912 | 38.78 | 38.53 | 0.0052 |
| Leflunomide, % | 27.54 | 15.56 | 0.2944 | 26.58 | 25.03 | 0.0355 |
| Sulfasalazine, % | 16.27 | 12.86 | 0.0968 | 16.06 | 15.52 | 0.0149 |
| Prednisone, % | 69.09 | 54.48 | 0.3041 | 68.44 | 67.52 | 0.0197 |
| Methylprednisolone, % | 55.46 | 33.88 | 0.4446 | 54.42 | 53.20 | 0.0245 |
| Dexamethasone, % | 36.61 | 22.26 | 0.3188 | 36.16 | 35.35 | 0.0168 |
| **Prior non-TNF biologic DMARDs** |  |  |  |  |  |  |
| Abatacept, % | 17.88 | 3.42 | 0.4824 | 15.88 | 13.65 | 0.0630 |
| Tocilizumab, % | 12.81 | 1.91 | 0.4268 | 10.76 | 8.53 | 0.0757 |
| Rituximab, % | 4.97 | 1.03 | 0.2329 | 4.26 | 3.46 | 0.0419 |
| **Laboratory parameters** |  |  |  |  |  |  |
| C-reactive protein, mg/L (mean ± SD) | 14.7 ± 29.5 | 16.0 ± 31.0 | 0.0443 | 14.7 ± 29.7 | 15.0 ± 28.4 | 0.0102 |
| C-reactive protein, mg/L (median [IQR]) | 5.9 [12.0] | 6.6 [12.9] | — | 5.9 [12.0] | 6.0 [12.5] | — |
| C-reactive protein recorded, % | 67.90 | 55.05 | 0.2663 | 67.28 | 67.21 | 0.0013 |
| Erythrocyte sedimentation rate, mm/h (mean ± SD) | 26.3 ± 24.2 | 27.4 ± 24.2 | 0.0472 | 26.2 ± 24.1 | 26.7 ± 24.0 | 0.0217 |
| Erythrocyte sedimentation rate, mm/h (median [IQR]) | 19 [28] | 20 [28] | — | 19 [27] | 19 [27] | — |
| Erythrocyte sedimentation rate recorded, % | 66.72 | 56.54 | 0.2107 | 66.20 | 66.00 | 0.0043 |

Baseline characteristics of patients initiating upadacitinib versus tumour necrosis factor inhibitors (TNFi), before and after 1:1 propensity score matching. The propensity score model included demographics, cardiovascular risk factors and comorbidities, other autoimmune and inflammatory diagnoses, conventional synthetic DMARDs, corticosteroids, prior non-TNF biologic DMARDs, and baseline inflammatory markers. Values are mean ± standard deviation (SD) or median [interquartile range] for continuous variables and percentages for categorical variables. Standardised mean differences (SMD) < 0.1 indicate adequate balance. Before matching, upadacitinib initiators were younger (65.4 versus 68.1 years), more heavily treated with csDMARDs and corticosteroids, and far more likely to have received a prior non-TNF biologic DMARD (abatacept SMD 0.482; tocilizumab 0.427; rituximab 0.233) than unmatched TNFi initiators. After matching all SMDs were < 0.08. The largest residual differences were for prior non-TNF biologic exposure — tocilizumab (0.076), abatacept (0.063) and rituximab (0.042) — each remaining more frequent in the upadacitinib arm; leflunomide (0.036), methylprednisolone (0.025) and prednisone (0.020) followed. All demographic and comorbidity SMDs were ≤ 0.016. Baseline C-reactive protein was well balanced after matching (14.7 ± 29.7 versus 15.0 ± 28.4 mg/L; SMD 0.010), as was erythrocyte sedimentation rate (SMD 0.022). C-reactive protein was recorded in approximately 67% of patients and erythrocyte sedimentation rate in approximately 66%; the proportion with a recorded value differed between groups before matching (SMD 0.266 and 0.211, respectively) but was balanced after matching (0.001 and 0.004). Interquartile range is reported as the width of the interquartile range. TNFi = tumour necrosis factor inhibitors; SD = standard deviation; SMD = standardised mean difference.

**Supplementary Table S4. Baseline characteristics in the pre-2021 tofacitinib versus TNFi propensity score–matched cohort**

| **Characteristic** | **Before: Tofacitinib (n = 8,160)** | **Before: TNFi (n = 31,643)** | **SMD** | **After: Tofacitinib (n = 7,777)** | **After: TNFi (n = 7,777)** | **SMD** |
| --- | --- | --- | --- | --- | --- | --- |
| **Demographics** |  |  |  |  |  |  |
| Age, current, years (mean ± SD) | 69.1 ± 9.21 | 69.7 ± 9.49 | 0.0687 | 69.1 ± 9.21 | 69.0 ± 9.37 | 0.0169 |
| Age at index, years (mean ± SD) | 61.0 ± 9.18 | 59.9 ± 9.38 | 0.1180 | 61.0 ± 9.18 | 61.0 ± 9.33 | 0.0046 |
| Female, % | 80.06 | 75.44 | 0.1113 | 79.77 | 80.07 | 0.0074 |
| Male, % | 19.94 | 24.56 | 0.1113 | 20.23 | 19.93 | 0.0074 |
| Unknown gender, % | 0 | 0 | — | 0 | 0 | — |
| White, % | 69.87 | 70.43 | 0.0124 | 69.81 | 70.10 | 0.0065 |
| Black or African American, % | 14.93 | 15.11 | 0.0050 | 14.97 | 14.84 | 0.0036 |
| Asian, % | 3.87 | 3.02 | 0.0467 | 3.83 | 3.83 | < 0.0001 |
| American Indian or Alaska Native, % | 0.63 | 0.75 | 0.0150 | 0.63 | 0.51 | 0.0153 |
| Native Hawaiian or Other Pacific Islander, % | 0.26 | 0.27 | 0.0028 | 0.27 | 0.23 | 0.0077 |
| Other race, % | 2.30 | 2.72 | 0.0263 | 2.37 | 2.55 | 0.0116 |
| Unknown race, % | 8.15 | 7.71 | 0.0165 | 8.13 | 7.93 | 0.0071 |
| Hispanic or Latino, % | 6.67 | 7.84 | 0.0453 | 6.66 | 6.69 | 0.0010 |
| Not Hispanic or Latino, % | 70.66 | 69.88 | 0.0170 | 70.57 | 70.81 | 0.0054 |
| Unknown ethnicity, % | 22.67 | 22.28 | 0.0095 | 22.77 | 22.50 | 0.0065 |
| **Cardiovascular risk factors and comorbidities** |  |  |  |  |  |  |
| Hypertension, % | 65.99 | 63.36 | 0.0551 | 65.78 | 65.45 | 0.0070 |
| Dyslipidemia, % | 52.70 | 46.56 | 0.1230 | 52.10 | 51.82 | 0.0057 |
| Overweight and obesity, % | 26.92 | 23.29 | 0.0839 | 26.80 | 26.86 | 0.0015 |
| Type 2 diabetes, % | 23.85 | 22.53 | 0.0312 | 23.71 | 23.61 | 0.0024 |
| Nicotine dependence, % | 17.61 | 17.39 | 0.0059 | 17.42 | 17.23 | 0.0051 |
| Tobacco use, % | 6.01 | 4.99 | 0.0445 | 5.89 | 5.75 | 0.0060 |
| Ischaemic heart disease, % | 16.72 | 14.48 | 0.0615 | 16.55 | 15.94 | 0.0164 |
| Cerebral infarction, % | 3.47 | 2.99 | 0.0269 | 3.50 | 3.83 | 0.0178 |
| Heart failure, % | 7.53 | 4.58 | 0.1238 | 7.18 | 7.20 | 0.0010 |
| Atrial fibrillation, % | 6.52 | 4.70 | 0.0792 | 6.35 | 6.40 | 0.0021 |
| Chronic kidney disease, % | 7.54 | 5.83 | 0.0684 | 7.41 | 7.41 | < 0.0001 |
| Acute kidney injury, % | 5.78 | 3.90 | 0.0880 | 5.63 | 5.22 | 0.0182 |
| **Other autoimmune and inflammatory diagnoses** |  |  |  |  |  |  |
| Systemic lupus erythematosus, % | 4.83 | 3.45 | 0.0694 | 4.58 | 4.23 | 0.0169 |
| Systemic sclerosis, % | 1.09 | 0.74 | 0.0365 | 0.95 | 1.04 | 0.0091 |
| Dermatomyositis, % | 0.67 | 0.40 | 0.0378 | 0.63 | 0.55 | 0.0101 |
| Antiphospholipid syndrome, % | 0.74 | 0.56 | 0.0215 | 0.73 | 0.76 | 0.0030 |
| Arthropathic psoriasis, % | 0.28 | 0.27 | 0.0031 | 0.28 | 0.26 | 0.0050 |
| Ankylosing spondylitis, % | 0.31 | 0.12 | 0.0396 | 0.30 | 0.21 | 0.0180 |
| Crohn's disease, % | 0.12 | 0.13 | 0.0011 | 0.13 | 0.14 | 0.0035 |
| Ulcerative colitis, % | 0.12 | 0.06 | 0.0207 | 0.13 | 0.13 | < 0.0001 |
| **csDMARDs and corticosteroids** |  |  |  |  |  |  |
| Methotrexate, % | 52.75 | 48.43 | 0.0864 | 52.13 | 52.53 | 0.0080 |
| Hydroxychloroquine, % | 36.23 | 27.79 | 0.1815 | 35.57 | 35.31 | 0.0054 |
| Leflunomide, % | 24.60 | 13.81 | 0.2765 | 23.51 | 23.27 | 0.0055 |
| Sulfasalazine, % | 17.16 | 12.86 | 0.1206 | 16.64 | 16.36 | 0.0076 |
| Prednisone, % | 63.98 | 50.74 | 0.2701 | 62.79 | 63.42 | 0.0131 |
| Methylprednisolone, % | 42.39 | 28.45 | 0.2946 | 41.04 | 40.49 | 0.0113 |
| Dexamethasone, % | 21.95 | 15.06 | 0.1781 | 21.31 | 21.00 | 0.0076 |
| **Prior non-TNF biologic DMARDs** |  |  |  |  |  |  |
| Abatacept, % | 12.70 | 2.57 | 0.3885 | 10.16 | 8.42 | 0.0598 |
| Tocilizumab, % | 8.43 | 1.45 | 0.3262 | 6.62 | 5.08 | 0.0658 |
| Rituximab, % | 4.27 | 0.89 | 0.2138 | 3.52 | 2.73 | 0.0458 |
| **Laboratory parameters** |  |  |  |  |  |  |
| C-reactive protein, mg/L (mean ± SD) | 15.5 ± 29.0 | 16.3 ± 31.1 | 0.0248 | 15.5 ± 28.5 | 16.0 ± 30.5 | 0.0169 |
| C-reactive protein, mg/L (median [IQR]) | 6.8 [12.9] | 7.0 [13.3] | — | 6.8 [12.8] | 7.0 [13.2] | — |
| C-reactive protein recorded, % | 60.87 | 51.35 | 0.1926 | 60.00 | 59.03 | 0.0196 |
| Erythrocyte sedimentation rate, mm/h (mean ± SD) | 28.3 ± 24.6 | 27.7 ± 24.4 | 0.0232 | 28.4 ± 24.5 | 28.3 ± 25.0 | 0.0038 |
| Erythrocyte sedimentation rate, mm/h (median [IQR]) | 21 [29] | 20 [28] | — | 21 [30] | 21 [29] | — |
| Erythrocyte sedimentation rate recorded, % | 61.74 | 54.59 | 0.1454 | 60.94 | 59.86 | 0.0221 |

Baseline characteristics of patients initiating tofacitinib before 2021 versus contemporaneous tumour necrosis factor inhibitor (TNFi) initiators, before and after 1:1 propensity score matching. The propensity score model included demographics, cardiovascular risk factors and comorbidities, other autoimmune and inflammatory diagnoses, conventional synthetic DMARDs, corticosteroids, prior non-TNF biologic DMARDs, and baseline inflammatory markers. Values are mean ± standard deviation (SD) or median [interquartile range] for continuous variables and percentages for categorical variables. Standardised mean differences (SMD) < 0.1 indicate adequate balance. Before matching, pre-2021 tofacitinib initiators had a substantially higher burden of csDMARD and corticosteroid exposure (methylprednisolone 42.4% versus 28.5%; leflunomide 24.6% versus 13.8%; prednisone 64.0% versus 50.7%), of cardiac and renal comorbidity (heart failure SMD 0.124; acute kidney injury 0.088), and of prior non-TNF biologic DMARD exposure (abatacept SMD 0.389; tocilizumab 0.326; rituximab 0.214). After matching all SMDs were < 0.07 and all demographic, comorbidity and csDMARD or corticosteroid SMDs were ≤ 0.019. The largest residual differences were for prior non-TNF biologic exposure — tocilizumab (0.066), abatacept (0.060) and rituximab (0.046) — each remaining more frequent in the tofacitinib arm; these reflect the smaller pool of biologic-experienced TNFi initiators available for matching within the pre-2021 window. Baseline C-reactive protein (15.5 ± 28.5 versus 16.0 ± 30.5 mg/L; SMD 0.017) and erythrocyte sedimentation rate (SMD 0.004) were well balanced after matching. C-reactive protein was recorded in approximately 60% of patients and erythrocyte sedimentation rate in approximately 60%; the proportion with a recorded value differed between groups before matching but was balanced after matching. Interquartile range is reported as the width of the interquartile range. TNFi = tumour necrosis factor inhibitors; SD = standard deviation; SMD = standardised mean difference.

**Supplementary Table S5. Baseline characteristics in the post-2021 tofacitinib versus TNFi propensity score–matched cohort**

| **Characteristic** | **Before: Tofacitinib (n = 4,826)** | **Before: TNFi (n = 24,123)** | **SMD** | **After: Tofacitinib (n = 4,802)** | **After: TNFi (n = 4,802)** | **SMD** |
| --- | --- | --- | --- | --- | --- | --- |
| **Demographics** |  |  |  |  |  |  |
| Age, current, years (mean ± SD) | 66.5 ± 9.08 | 66.2 ± 9.28 | 0.0377 | 66.6 ± 9.08 | 66.7 ± 9.43 | 0.0181 |
| Age at index, years (mean ± SD) | 63.2 ± 9.06 | 63.4 ± 9.31 | 0.0236 | 63.2 ± 9.06 | 63.3 ± 9.44 | 0.0150 |
| Female, % | 79.42 | 74.75 | 0.1115 | 79.38 | 78.88 | 0.0123 |
| Male, % | 20.56 | 25.24 | 0.1117 | 20.60 | 21.10 | 0.0123 |
| Unknown gender, % | 0.21 | 0.04 | 0.0471 | 0.21 | 0.21 | < 0.0001 |
| White, % | 63.03 | 68.61 | 0.1177 | 63.02 | 62.97 | 0.0009 |
| Black or African American, % | 14.19 | 15.00 | 0.0229 | 14.24 | 13.97 | 0.0078 |
| Asian, % | 4.48 | 2.63 | 0.0997 | 4.44 | 4.44 | < 0.0001 |
| American Indian or Alaska Native, % | 0.66 | 0.86 | 0.0224 | 0.65 | 0.54 | 0.0136 |
| Native Hawaiian or Other Pacific Islander, % | 0.21 | 0.29 | 0.0174 | 0.21 | 0.21 | < 0.0001 |
| Other race, % | 3.23 | 3.54 | 0.0168 | 3.23 | 3.48 | 0.0139 |
| Unknown race, % | 14.19 | 9.07 | 0.1603 | 14.22 | 14.43 | 0.0059 |
| Hispanic or Latino, % | 6.96 | 7.54 | 0.0221 | 6.96 | 6.85 | 0.0041 |
| Not Hispanic or Latino, % | 64.44 | 65.99 | 0.0324 | 64.41 | 64.12 | 0.0061 |
| Unknown ethnicity, % | 28.60 | 26.48 | 0.0474 | 28.63 | 29.03 | 0.0087 |
| **Cardiovascular risk factors and comorbidities** |  |  |  |  |  |  |
| Hypertension, % | 66.72 | 67.74 | 0.0217 | 66.74 | 66.76 | 0.0004 |
| Dyslipidemia, % | 58.14 | 59.12 | 0.0198 | 58.10 | 58.85 | 0.0152 |
| Overweight and obesity, % | 31.83 | 33.80 | 0.0421 | 31.82 | 31.74 | 0.0018 |
| Type 2 diabetes, % | 26.01 | 26.02 | 0.0004 | 26.03 | 25.28 | 0.0172 |
| Nicotine dependence, % | 17.20 | 19.83 | 0.0678 | 17.22 | 16.83 | 0.0105 |
| Tobacco use, % | 6.47 | 7.59 | 0.0442 | 6.48 | 6.35 | 0.0051 |
| Ischaemic heart disease, % | 16.27 | 19.36 | 0.0808 | 16.24 | 15.66 | 0.0159 |
| Cerebral infarction, % | 3.03 | 4.24 | 0.0648 | 3.04 | 3.17 | 0.0072 |
| Heart failure, % | 7.90 | 7.15 | 0.0284 | 7.85 | 7.68 | 0.0062 |
| Atrial fibrillation, % | 5.78 | 7.20 | 0.0576 | 5.79 | 5.79 | < 0.0001 |
| Chronic kidney disease, % | 9.57 | 10.40 | 0.0275 | 9.62 | 9.27 | 0.0121 |
| Acute kidney injury, % | 7.09 | 6.99 | 0.0038 | 7.12 | 6.73 | 0.0156 |
| **Other autoimmune and inflammatory diagnoses** |  |  |  |  |  |  |
| Systemic lupus erythematosus, % | 5.33 | 3.41 | 0.0937 | 5.21 | 4.64 | 0.0260 |
| Systemic sclerosis, % | 1.10 | 0.62 | 0.0521 | 1.06 | 1.13 | 0.0060 |
| Dermatomyositis, % | 1.62 | 0.36 | 0.1272 | 1.46 | 1.27 | 0.0162 |
| Antiphospholipid syndrome, % | 0.41 | 0.54 | 0.0175 | 0.42 | 0.48 | 0.0094 |
| Arthropathic psoriasis, % | 0.56 | 0.31 | 0.0385 | 0.56 | 0.58 | 0.0028 |
| Ankylosing spondylitis, % | 0.33 | 0.22 | 0.0222 | 0.33 | 0.29 | 0.0075 |
| Crohn's disease, % | 0.25 | 0.17 | 0.0172 | 0.25 | 0.21 | 0.0087 |
| Ulcerative colitis, % | 0.21 | 0.22 | 0.0036 | 0.21 | 0.21 | < 0.0001 |
| **csDMARDs and corticosteroids** |  |  |  |  |  |  |
| Methotrexate, % | 49.11 | 51.46 | 0.0471 | 49.08 | 47.92 | 0.0233 |
| Hydroxychloroquine, % | 35.45 | 32.87 | 0.0544 | 35.30 | 34.67 | 0.0131 |
| Leflunomide, % | 22.40 | 17.15 | 0.1320 | 22.22 | 22.80 | 0.0140 |
| Sulfasalazine, % | 16.14 | 12.47 | 0.1050 | 16.12 | 16.29 | 0.0045 |
| Prednisone, % | 58.12 | 58.45 | 0.0066 | 57.96 | 57.96 | < 0.0001 |
| Methylprednisolone, % | 41.96 | 39.71 | 0.0458 | 41.80 | 40.59 | 0.0245 |
| Dexamethasone, % | 30.98 | 30.81 | 0.0038 | 30.95 | 30.03 | 0.0199 |
| **Prior non-TNF biologic DMARDs** |  |  |  |  |  |  |
| Abatacept, % | 11.44 | 3.81 | 0.2903 | 11.06 | 10.75 | 0.0100 |
| Tocilizumab, % | 6.98 | 2.06 | 0.2386 | 6.69 | 6.48 | 0.0084 |
| Rituximab, % | 3.25 | 1.04 | 0.1531 | 3.06 | 2.92 | 0.0086 |
| **Laboratory parameters** |  |  |  |  |  |  |
| C-reactive protein, mg/L (mean ± SD) | 15.9 ± 30.6 | 16.2 ± 30.9 | 0.0105 | 15.9 ± 30.6 | 16.2 ± 30.2 | 0.0104 |
| C-reactive protein, mg/L (median [IQR]) | 6.0 [12.9] | 6.6 [13.2] | — | 6.0 [13.0] | 6.8 [13.6] | — |
| C-reactive protein recorded, % | 59.84 | 58.69 | 0.0235 | 59.75 | 58.35 | 0.0284 |
| Erythrocyte sedimentation rate, mm/h (mean ± SD) | 28.9 ± 25.4 | 27.6 ± 24.2 | 0.0527 | 29.0 ± 25.5 | 29.2 ± 25.3 | 0.0076 |
| Erythrocyte sedimentation rate, mm/h (median [IQR]) | 21 [31] | 20 [28] | — | 21 [31] | 22 [31] | — |
| Erythrocyte sedimentation rate recorded, % | 57.75 | 58.29 | 0.0110 | 57.62 | 56.12 | 0.0303 |

Baseline characteristics of patients initiating tofacitinib from 2021 onward versus contemporaneous tumour necrosis factor inhibitor (TNFi) initiators, before and after 1:1 propensity score matching. The propensity score model included demographics, cardiovascular risk factors and comorbidities, other autoimmune and inflammatory diagnoses, conventional synthetic DMARDs, corticosteroids, prior non-TNF biologic DMARDs, and baseline inflammatory markers. Values are mean ± standard deviation (SD) or median [interquartile range] for continuous variables and percentages for categorical variables. Standardised mean differences (SMD) < 0.1 indicate adequate balance. In contrast to the pre-2021 era, post-2021 tofacitinib and TNFi initiators were closely comparable before matching on age (SMD 0.038), hypertension (0.022), dyslipidemia (0.020), type 2 diabetes (< 0.001), prednisone (0.007) and methotrexate (0.047), and TNFi initiators carried a higher burden of ischaemic heart disease (19.4% versus 16.3%) and atrial fibrillation (7.2% versus 5.8%). Residual channeling before matching was concentrated in prior non-TNF biologic DMARD exposure (abatacept SMD 0.290; tocilizumab 0.239; rituximab 0.153) rather than in cardiovascular risk or corticosteroid burden. After matching all SMDs were ≤ 0.031; the largest residual differences were for the proportion with a recorded erythrocyte sedimentation rate (0.030) and C-reactive protein (0.028), systemic lupus erythematosus (0.026), methylprednisolone (0.025) and methotrexate (0.023). Baseline C-reactive protein (15.9 ± 30.6 versus 16.2 ± 30.2 mg/L; SMD 0.010) and erythrocyte sedimentation rate (SMD 0.008) were well balanced. Interquartile range is reported as the width of the interquartile range. TNFi = tumour necrosis factor inhibitors; SD = standard deviation; SMD = standardised mean difference.

**Supplementary Table S6. Baseline characteristics in the pre-2021 baricitinib versus TNFi propensity score–matched cohort**

| **Characteristic** | **Before: Baricitinib (n = 481)** | **Before: TNFi (n = 31,643)** | **SMD** | **After: Baricitinib (n = 469)** | **After: TNFi (n = 469)** | **SMD** |
| --- | --- | --- | --- | --- | --- | --- |
| **Demographics** |  |  |  |  |  |  |
| Age, current, years (mean ± SD) | 68.1 ± 9.11 | 69.7 ± 9.49 | 0.1763 | 68.0 ± 9.12 | 68.2 ± 9.49 | 0.0213 |
| Age at index, years (mean ± SD) | 61.3 ± 9.12 | 59.9 ± 9.38 | 0.1518 | 61.2 ± 9.13 | 61.5 ± 9.53 | 0.0311 |
| Female, % | 80.04 | 75.44 | 0.1108 | 80.17 | 80.17 | < 0.0001 |
| Male, % | 19.96 | 24.56 | 0.1108 | 19.83 | 19.83 | < 0.0001 |
| Unknown gender, % | 0 | 0 | — | 0 | 0 | — |
| White, % | 55.72 | 70.43 | 0.3085 | 56.50 | 53.09 | 0.0686 |
| Black or African American, % | 8.32 | 15.11 | 0.2124 | 8.10 | 8.10 | < 0.0001 |
| Asian, % | 7.07 | 3.02 | 0.1857 | 6.61 | 6.18 | 0.0174 |
| American Indian or Alaska Native, % | 2.08 | 0.75 | 0.1128 | 2.13 | 2.13 | < 0.0001 |
| Native Hawaiian or Other Pacific Islander, % | 2.08 | 0.27 | 0.1683 | 2.13 | 0 | 0.2087 ‡ |
| Other race, % | 3.95 | 2.72 | 0.0689 | 4.05 | 4.26 | 0.0107 |
| Unknown race, % | 24.12 | 7.71 | 0.4604 | 23.88 | 27.93 | 0.0926 |
| Hispanic or Latino, % | 7.48 | 7.84 | 0.0134 | 7.68 | 9.17 | 0.0538 |
| Not Hispanic or Latino, % | 49.27 | 69.88 | 0.4296 | 48.83 | 45.20 | 0.0727 |
| Unknown ethnicity, % | 43.24 | 22.28 | 0.4583 | 43.50 | 45.63 | 0.0429 |
| **Cardiovascular risk factors and comorbidities** |  |  |  |  |  |  |
| Hypertension, % | 62.58 | 63.36 | 0.0162 | 62.26 | 62.69 | 0.0088 |
| Dyslipidemia, % | 53.64 | 46.56 | 0.1420 | 53.09 | 50.96 | 0.0427 |
| Overweight and obesity, % | 30.15 | 23.29 | 0.1554 | 30.06 | 26.65 | 0.0757 |
| Type 2 diabetes, % | 25.78 | 22.53 | 0.0760 | 25.16 | 26.23 | 0.0244 |
| Nicotine dependence, % | 20.79 | 17.39 | 0.0867 | 20.68 | 19.62 | 0.0266 |
| Tobacco use, % | 4.37 | 4.99 | 0.0296 | 4.26 | 3.84 | 0.0216 |
| Ischaemic heart disease, % | 17.05 | 14.48 | 0.0704 | 17.06 | 16.63 | 0.0114 |
| Cerebral infarction, % | 3.53 | 2.99 | 0.0305 | 3.41 | 2.99 | 0.0242 |
| Heart failure, % | 7.07 | 4.58 | 0.1064 | 7.04 | 6.61 | 0.0169 |
| Atrial fibrillation, % | 6.24 | 4.70 | 0.0677 | 6.18 | 7.04 | 0.0343 |
| Chronic kidney disease, % | 9.36 | 5.83 | 0.1334 | 8.96 | 10.02 | 0.0364 |
| Acute kidney injury, % | 7.28 | 3.90 | 0.1476 | 7.04 | 6.61 | 0.0169 |
| **Other autoimmune and inflammatory diagnoses** |  |  |  |  |  |  |
| Systemic lupus erythematosus, % | 6.65 | 3.45 | 0.1468 | 6.18 | 7.04 | 0.0343 |
| Systemic sclerosis, % | 2.08 | 0.74 | 0.1135 | 2.13 | 0 | 0.2087 ‡ |
| Dermatomyositis, % | 2.08 | 0.40 | 0.1524 | 2.13 | 0 | 0.2087 ‡ |
| Antiphospholipid syndrome, % | 2.08 | 0.56 | 0.1331 | 2.13 | 2.13 | < 0.0001 |
| Arthropathic psoriasis, % | 2.08 | 0.27 | 0.1691 | 2.13 | 2.13 | < 0.0001 |
| Ankylosing spondylitis, % | 0 | 0.12 | 0.0497 | 0 | 0 | — |
| Crohn's disease, % | 0 | 0.13 | 0.0503 | 0 | 0 | — |
| Ulcerative colitis, % | 0 | 0.06 | 0.0347 | 0 | 2.13 | 0.2087 ‡ |
| **csDMARDs and corticosteroids** |  |  |  |  |  |  |
| Methotrexate, % | 46.36 | 48.43 | 0.0414 | 46.48 | 43.92 | 0.0514 |
| Hydroxychloroquine, % | 33.26 | 27.79 | 0.1190 | 32.62 | 33.90 | 0.0272 |
| Leflunomide, % | 27.44 | 13.81 | 0.3419 | 26.65 | 25.80 | 0.0194 |
| Sulfasalazine, % | 20.37 | 12.86 | 0.2030 | 20.04 | 20.68 | 0.0159 |
| Prednisone, % | 54.89 | 50.74 | 0.0830 | 55.01 | 53.31 | 0.0342 |
| Methylprednisolone, % | 42.62 | 28.45 | 0.2993 | 42.22 | 39.23 | 0.0608 |
| Dexamethasone, % | 22.45 | 15.06 | 0.1903 | 21.75 | 19.62 | 0.0527 |
| **Prior non-TNF biologic DMARDs** |  |  |  |  |  |  |
| Abatacept, % | 17.67 | 2.57 | 0.5172 | 16.63 | 12.58 | 0.1149 |
| Tocilizumab, % | 19.13 | 1.45 | 0.6080 | 17.27 | 14.71 | 0.0699 |
| Rituximab, % | 10.81 | 0.89 | 0.4322 | 9.17 | 8.10 | 0.0380 |
| **Laboratory parameters** |  |  |  |  |  |  |
| C-reactive protein, mg/L (mean ± SD) | 15.4 ± 32.5 | 16.3 ± 31.1 | 0.0256 | 15.7 ± 33.1 | 14.5 ± 26.2 | 0.0407 |
| C-reactive protein, mg/L (median [IQR]) | 6.3 [13.0] | 7.0 [13.3] | — | 6.0 [13.0] | 6.0 [12.5] | — |
| C-reactive protein recorded, % | 64.45 | 51.35 | 0.2676 | 63.54 | 63.11 | 0.0088 |
| Erythrocyte sedimentation rate, mm/h (mean ± SD) | 29.4 ± 26.7 | 27.7 ± 24.4 | 0.0668 | 29.2 ± 26.6 | 28.4 ± 24.2 | 0.0313 |
| Erythrocyte sedimentation rate, mm/h (median [IQR]) | 22 [33] | 20 [28] | — | 22 [33] | 21 [31] | — |
| Erythrocyte sedimentation rate recorded, % | 47.19 | 54.59 | 0.1483 | 47.12 | 46.06 | 0.0214 |

‡ Standardised mean difference derived from a cell in which one arm contained fewer than the minimum reportable patient count; the platform reports such counts as 10 or 0, so these values reflect count obfuscation rather than a measurable imbalance and should not be interpreted as evidence of poor covariate balance.

Baseline characteristics of patients initiating baricitinib before 2021 versus contemporaneous tumour necrosis factor inhibitor (TNFi) initiators, before and after 1:1 propensity score matching. The propensity score model included demographics, cardiovascular risk factors and comorbidities, other autoimmune and inflammatory diagnoses, conventional synthetic DMARDs, corticosteroids, prior non-TNF biologic DMARDs, and baseline inflammatory markers. Values are mean ± standard deviation (SD) or median [interquartile range] for continuous variables and percentages for categorical variables. Standardised mean differences (SMD) < 0.1 indicate adequate balance. This is the smallest matched cohort in the study (469 pairs), reflecting limited baricitinib uptake before 2021, and covariate balance after matching is correspondingly the least complete of any stratum. Excluding covariates affected by low-count obfuscation, the largest residual difference was for prior abatacept exposure (16.6% versus 12.6%; SMD 0.115), which exceeded the conventional 0.1 threshold, followed by unknown race (0.093), overweight and obesity (0.076), non-Hispanic or Latino ethnicity (0.073), prior tocilizumab exposure (0.070), White race (0.069) and methylprednisolone (0.061). Cardiovascular comorbidities were well balanced (hypertension 0.009, ischaemic heart disease 0.011, heart failure 0.017, chronic kidney disease 0.036), as were baseline C-reactive protein (15.7 ± 33.1 versus 14.5 ± 26.2 mg/L; SMD 0.041) and erythrocyte sedimentation rate (SMD 0.031); the nominally significant *P* value for C-reactive protein after matching (*P* = 0.021) reflects a distributional rather than a mean difference. Given the cohort size and the residual imbalance in prior non-TNF biologic DMARD exposure — which marks more refractory disease and is not fully removed by matching — estimates derived from this stratum should be regarded as hypothesis-generating and are not interpreted as evidence of a drug-specific effect. Interquartile range is reported as the width of the interquartile range. TNFi = tumour necrosis factor inhibitors; SD = standard deviation; SMD = standardised mean difference.

**Supplementary Table S7. Baseline characteristics in the post-2021 baricitinib versus TNFi propensity score–matched cohort**

| **Characteristic** | **Before: Baricitinib (n = 1,231)** | **Before: TNFi (n = 24,123)** | **SMD** | **After: Baricitinib (n = 1,205)** | **After: TNFi (n = 1,205)** | **SMD** |
| --- | --- | --- | --- | --- | --- | --- |
| **Demographics** |  |  |  |  |  |  |
| Age, current, years (mean ± SD) | 69.0 ± 9.64 | 66.2 ± 9.28 | 0.2998 | 68.9 ± 9.62 | 69.1 ± 9.80 | 0.0218 |
| Age at index, years (mean ± SD) | 65.3 ± 9.61 | 63.4 ± 9.31 | 0.2012 | 65.2 ± 9.60 | 65.5 ± 9.89 | 0.0253 |
| Female, % | 74.82 | 74.75 | 0.0016 | 75.35 | 75.19 | 0.0038 |
| Male, % | 25.18 | 25.24 | 0.0014 | 24.65 | 24.81 | 0.0038 |
| Unknown gender, % | 0 | 0.04 | 0.0288 | 0 | 0 | — |
| White, % | 61.82 | 68.61 | 0.1429 | 62.08 | 58.26 | 0.0780 |
| Black or African American, % | 10.40 | 15.00 | 0.1386 | 10.62 | 10.87 | 0.0080 |
| Asian, % | 3.66 | 2.63 | 0.0587 | 3.40 | 3.90 | 0.0265 |
| American Indian or Alaska Native, % | 1.14 | 0.86 | 0.0281 | 1.16 | 1.08 | 0.0079 |
| Native Hawaiian or Other Pacific Islander, % | 0.81 | 0.29 | 0.0699 | 0.83 | 0.83 | < 0.0001 |
| Other race, % | 1.87 | 3.54 | 0.1030 | 1.91 | 2.82 | 0.0601 |
| Unknown race, % | 20.80 | 9.07 | 0.3335 | 20.50 | 22.74 | 0.0545 |
| Hispanic or Latino, % | 6.17 | 7.54 | 0.0539 | 6.31 | 6.64 | 0.0135 |
| Not Hispanic or Latino, % | 57.03 | 65.99 | 0.1849 | 57.18 | 54.77 | 0.0485 |
| Unknown ethnicity, % | 36.80 | 26.48 | 0.2233 | 36.52 | 38.59 | 0.0429 |
| **Cardiovascular risk factors and comorbidities** |  |  |  |  |  |  |
| Hypertension, % | 71.81 | 67.74 | 0.0887 | 71.95 | 74.27 | 0.0524 |
| Dyslipidemia, % | 61.17 | 59.12 | 0.0419 | 61.16 | 61.99 | 0.0171 |
| Overweight and obesity, % | 39.81 | 33.80 | 0.1247 | 39.75 | 37.43 | 0.0477 |
| Type 2 diabetes, % | 35.42 | 26.02 | 0.2048 | 35.02 | 35.85 | 0.0174 |
| Nicotine dependence, % | 19.33 | 19.83 | 0.0125 | 18.92 | 18.67 | 0.0064 |
| Tobacco use, % | 6.42 | 7.59 | 0.0461 | 6.47 | 6.56 | 0.0034 |
| Ischaemic heart disease, % | 32.09 | 19.36 | 0.2944 | 31.62 | 32.03 | 0.0089 |
| Cerebral infarction, % | 6.34 | 4.24 | 0.0939 | 6.22 | 6.56 | 0.0136 |
| Heart failure, % | 19.17 | 7.15 | 0.3615 | 18.26 | 18.51 | 0.0064 |
| Atrial fibrillation, % | 15.44 | 7.20 | 0.2621 | 14.94 | 15.60 | 0.0185 |
| Chronic kidney disease, % | 20.96 | 10.40 | 0.2936 | 20.17 | 21.49 | 0.0327 |
| Acute kidney injury, % | 24.78 | 6.99 | 0.5017 | 23.73 | 24.32 | 0.0136 |
| **Other autoimmune and inflammatory diagnoses** |  |  |  |  |  |  |
| Systemic lupus erythematosus, % | 6.66 | 3.41 | 0.1490 | 6.64 | 6.14 | 0.0204 |
| Systemic sclerosis, % | 1.30 | 0.62 | 0.0700 | 1.33 | 0.83 | 0.0482 |
| Dermatomyositis, % | 1.46 | 0.36 | 0.1161 | 1.41 | 1.33 | 0.0071 |
| Antiphospholipid syndrome, % | 0.81 | 0.54 | 0.0339 | 0.83 | 0.83 | < 0.0001 |
| Arthropathic psoriasis, % | 0.81 | 0.31 | 0.0678 | 0.83 | 0 | 0.1294 ‡ |
| Ankylosing spondylitis, % | 0.81 | 0.22 | 0.0835 | 0.83 | 0.83 | < 0.0001 |
| Crohn's disease, % | 0.81 | 0.17 | 0.0920 | 0.83 | 0.83 | < 0.0001 |
| Ulcerative colitis, % | 0.81 | 0.22 | 0.0820 | 0.83 | 0.83 | < 0.0001 |
| **csDMARDs and corticosteroids** |  |  |  |  |  |  |
| Methotrexate, % | 34.53 | 51.46 | 0.3472 | 34.77 | 35.93 | 0.0243 |
| Hydroxychloroquine, % | 33.88 | 32.87 | 0.0212 | 33.78 | 34.94 | 0.0245 |
| Leflunomide, % | 18.03 | 17.15 | 0.0231 | 18.09 | 19.42 | 0.0340 |
| Sulfasalazine, % | 15.11 | 12.47 | 0.0766 | 15.10 | 15.85 | 0.0207 |
| Prednisone, % | 46.39 | 58.45 | 0.2433 | 46.47 | 47.05 | 0.0116 |
| Methylprednisolone, % | 44.76 | 39.71 | 0.1024 | 44.73 | 45.81 | 0.0217 |
| Dexamethasone, % | 49.80 | 30.81 | 0.3947 | 49.05 | 47.22 | 0.0365 |
| **Prior non-TNF biologic DMARDs** |  |  |  |  |  |  |
| Abatacept, % | 10.15 | 3.81 | 0.2507 | 9.71 | 10.62 | 0.0302 |
| Tocilizumab, % | 9.91 | 2.06 | 0.3356 | 9.38 | 10.04 | 0.0224 |
| Rituximab, % | 6.09 | 1.04 | 0.2750 | 5.64 | 5.15 | 0.0220 |
| **Laboratory parameters** |  |  |  |  |  |  |
| C-reactive protein, mg/L (mean ± SD) | 40.5 ± 60.1 | 16.2 ± 30.9 | 0.5082 | 40.4 ± 60.4 | 21.7 ± 37.3 | 0.3734 |
| C-reactive protein, mg/L (median [IQR]) | 12.0 [48.2] | 6.6 [13.2] | — | 12.0 [47.1] | 7.1 [19.0] | — |
| C-reactive protein recorded, % | 63.85 | 58.69 | 0.1062 | 63.49 | 64.65 | 0.0242 |
| Erythrocyte sedimentation rate, mm/h (mean ± SD) | 30.0 ± 25.4 | 27.6 ± 24.2 | 0.0970 | 29.9 ± 25.2 | 32.7 ± 27.7 | 0.1023 |
| Erythrocyte sedimentation rate, mm/h (median [IQR]) | 22 [34] | 20 [28] | — | 22 [33] | 24 [34] | — |
| Erythrocyte sedimentation rate recorded, % | 48.58 | 58.29 | 0.1957 | 48.47 | 47.97 | 0.0100 |

‡ Standardised mean difference derived from a cell in which one arm contained fewer than the minimum reportable patient count; the platform reports such counts as 10 or 0, so this value reflects count obfuscation rather than a measurable imbalance.

Baseline characteristics of patients initiating baricitinib from 2021 onward versus contemporaneous tumour necrosis factor inhibitor (TNFi) initiators, before and after 1:1 propensity score matching. The propensity score model included demographics, cardiovascular risk factors and comorbidities, other autoimmune and inflammatory diagnoses, conventional synthetic DMARDs, corticosteroids, prior non-TNF biologic DMARDs, and baseline inflammatory markers. Values are mean ± standard deviation (SD) or median [interquartile range] for continuous variables and percentages for categorical variables. Standardised mean differences (SMD) < 0.1 indicate adequate balance. Before matching, post-2021 baricitinib initiators carried a markedly higher burden of comorbidity than contemporaneous TNFi initiators (acute kidney injury 24.8% versus 7.0%; heart failure 19.2% versus 7.1%; chronic kidney disease 21.0% versus 10.4%; ischaemic heart disease 32.1% versus 19.4%) and were less likely to be receiving methotrexate (34.5% versus 51.5%) or prednisone (46.4% versus 58.4%). Matching resolved all diagnosis-based covariates to SMDs ≤ 0.053 and all csDMARD, corticosteroid and prior biologic covariates to SMDs ≤ 0.037; among categorical covariates the largest residual differences were for White race (0.078), other race (0.060), unknown race (0.055) and hypertension (0.052). Baseline C-reactive protein remained substantially imbalanced after matching (40.4 ± 60.4 versus 21.7 ± 37.3 mg/L; SMD 0.373), the largest residual difference in any cohort in this study, indicating that post-2021 baricitinib initiators carried an inflammatory burden for which no comparable TNFi population existed in the source data. Erythrocyte sedimentation rate was marginally imbalanced in the opposite direction (29.9 ± 25.2 versus 32.7 ± 27.7 mm/h; SMD 0.102); the two markers were recorded in different and partly non-overlapping subsets (C-reactive protein in approximately 63–65% of patients, erythrocyte sedimentation rate in approximately 48%), and the C-reactive protein distribution was strongly right-skewed (median 12.0 mg/L, interquartile range width 47.1), consistent with a subgroup of patients with acute intercurrent illness at index. Estimates derived from this stratum are therefore susceptible to residual confounding by disease severity and are interpreted as hypothesis-generating. Interquartile range is reported as the width of the interquartile range. TNFi = tumour necrosis factor inhibitors; SD = standard deviation; SMD = standardised mean difference.

**Supplementary Table S8. Baseline characteristics in the pre-2021 upadacitinib versus TNFi propensity score–matched cohort**

| **Characteristic** | **Before: Upadacitinib (n = 1,060)** | **Before: TNFi (n = 31,643)** | **SMD** | **After: Upadacitinib (n = 992)** | **After: TNFi (n = 992)** | **SMD** |
| --- | --- | --- | --- | --- | --- | --- |
| **Demographics** |  |  |  |  |  |  |
| Age, current, years (mean ± SD) | 67.2 ± 8.88 | 69.7 ± 9.49 | 0.2798 | 67.1 ± 8.89 | 67.2 ± 9.17 | 0.0095 |
| Age at index, years (mean ± SD) | 61.1 ± 8.89 | 59.9 ± 9.38 | 0.1253 | 61.0 ± 8.90 | 61.1 ± 9.17 | 0.0177 |
| Female, % | 82.93 | 75.44 | 0.1852 | 82.06 | 83.17 | 0.0293 |
| Male, % | 17.08 | 24.56 | 0.1852 | 17.94 | 16.84 | 0.0293 |
| Unknown gender, % | 0 | 0 | — | 0 | 0 | — |
| White, % | 73.96 | 70.43 | 0.0788 | 74.19 | 72.48 | 0.0388 |
| Black or African American, % | 14.15 | 15.11 | 0.0270 | 14.11 | 14.32 | 0.0058 |
| Asian, % | 3.96 | 3.02 | 0.0513 | 3.63 | 3.93 | 0.0159 |
| American Indian or Alaska Native, % | 0.94 | 0.75 | 0.0212 | 1.01 | 1.01 | < 0.0001 |
| Native Hawaiian or Other Pacific Islander, % | 0.94 | 0.27 | 0.0865 | 1.01 | 1.01 | < 0.0001 |
| Other race, % | 1.70 | 2.72 | 0.0692 | 1.71 | 1.71 | < 0.0001 |
| Unknown race, % | 4.62 | 7.71 | 0.1284 | 4.94 | 5.75 | 0.0359 |
| Hispanic or Latino, % | 7.93 | 7.84 | 0.0031 | 8.07 | 7.96 | 0.0037 |
| Not Hispanic or Latino, % | 72.93 | 69.88 | 0.0674 | 72.58 | 71.37 | 0.0269 |
| Unknown ethnicity, % | 19.15 | 22.28 | 0.0772 | 19.36 | 20.67 | 0.0328 |
| **Cardiovascular risk factors and comorbidities** |  |  |  |  |  |  |
| Hypertension, % | 69.25 | 63.36 | 0.1248 | 68.75 | 68.85 | 0.0022 |
| Dyslipidemia, % | 58.21 | 46.56 | 0.2349 | 57.56 | 56.96 | 0.0122 |
| Overweight and obesity, % | 36.51 | 23.29 | 0.2919 | 35.79 | 35.48 | 0.0063 |
| Type 2 diabetes, % | 24.81 | 22.53 | 0.0537 | 24.09 | 22.28 | 0.0430 |
| Nicotine dependence, % | 18.40 | 17.39 | 0.0264 | 18.25 | 17.44 | 0.0211 |
| Tobacco use, % | 7.93 | 4.99 | 0.1196 | 7.86 | 7.26 | 0.0229 |
| Ischaemic heart disease, % | 18.11 | 14.48 | 0.0984 | 18.15 | 17.64 | 0.0132 |
| Cerebral infarction, % | 3.59 | 2.99 | 0.0332 | 3.63 | 2.72 | 0.0518 |
| Heart failure, % | 6.89 | 4.58 | 0.0994 | 7.06 | 6.96 | 0.0039 |
| Atrial fibrillation, % | 5.38 | 4.70 | 0.0310 | 5.54 | 6.05 | 0.0216 |
| Chronic kidney disease, % | 8.68 | 5.83 | 0.1100 | 8.57 | 8.27 | 0.0109 |
| Acute kidney injury, % | 7.17 | 3.90 | 0.1435 | 7.16 | 6.35 | 0.0321 |
| **Other autoimmune and inflammatory diagnoses** |  |  |  |  |  |  |
| Systemic lupus erythematosus, % | 6.98 | 3.45 | 0.1594 | 6.45 | 5.14 | 0.0561 |
| Systemic sclerosis, % | 1.04 | 0.74 | 0.0314 | 1.11 | 1.01 | 0.0099 |
| Dermatomyositis, % | 0.94 | 0.40 | 0.0668 | 1.01 | 1.01 | < 0.0001 |
| Antiphospholipid syndrome, % | 0.94 | 0.56 | 0.0441 | 1.01 | 1.01 | < 0.0001 |
| Arthropathic psoriasis, % | 0.94 | 0.27 | 0.0875 | 1.01 | 1.01 | < 0.0001 |
| Ankylosing spondylitis, % | 0.94 | 0.12 | 0.1128 | 1.01 | 0 | 0.1427 ‡ |
| Crohn's disease, % | 0.94 | 0.13 | 0.1122 | 1.01 | 1.01 | < 0.0001 |
| Ulcerative colitis, % | 0.94 | 0.06 | 0.1253 | 1.01 | 1.01 | < 0.0001 |
| **csDMARDs and corticosteroids** |  |  |  |  |  |  |
| Methotrexate, % | 58.87 | 48.43 | 0.2105 | 57.76 | 56.65 | 0.0224 |
| Hydroxychloroquine, % | 37.36 | 27.79 | 0.2051 | 37.20 | 36.29 | 0.0188 |
| Leflunomide, % | 31.13 | 13.81 | 0.4243 | 29.13 | 29.44 | 0.0066 |
| Sulfasalazine, % | 16.79 | 12.86 | 0.1110 | 16.73 | 15.93 | 0.0218 |
| Prednisone, % | 77.17 | 50.74 | 0.5725 | 76.01 | 76.11 | 0.0024 |
| Methylprednisolone, % | 60.09 | 28.45 | 0.6720 | 58.17 | 57.06 | 0.0224 |
| Dexamethasone, % | 28.77 | 15.06 | 0.3362 | 28.23 | 27.82 | 0.0090 |
| **Prior non-TNF biologic DMARDs** |  |  |  |  |  |  |
| Abatacept, % | 21.70 | 2.57 | 0.6127 | 18.35 | 15.73 | 0.0698 |
| Tocilizumab, % | 16.70 | 1.45 | 0.5504 | 12.40 | 9.38 | 0.0972 |
| Rituximab, % | 7.45 | 0.89 | 0.3324 | 5.65 | 4.23 | 0.0652 |
| **Laboratory parameters** |  |  |  |  |  |  |
| C-reactive protein, mg/L (mean ± SD) | 14.5 ± 26.8 | 16.3 ± 31.1 | 0.0607 | 15.0 ± 27.7 | 15.3 ± 29.9 | 0.0099 |
| C-reactive protein, mg/L (median [IQR]) | 6.0 [12.5] | 7.0 [13.3] | — | 6.2 [12.8] | 7.0 [12.6] | — |
| C-reactive protein recorded, % | 71.04 | 51.35 | 0.4124 | 69.76 | 68.85 | 0.0197 |
| Erythrocyte sedimentation rate, mm/h (mean ± SD) | 26.4 ± 23.5 | 27.7 ± 24.4 | 0.0528 | 26.5 ± 23.7 | 28.1 ± 24.3 | 0.0663 |
| Erythrocyte sedimentation rate, mm/h (median [IQR]) | 19 [28] | 20 [28] | — | 19 [27] | 20 [28] | — |
| Erythrocyte sedimentation rate recorded, % | 71.89 | 54.59 | 0.3647 | 70.46 | 69.56 | 0.0198 |

‡ Standardised mean difference derived from a cell in which one arm contained fewer than the minimum reportable patient count; the platform reports such counts as 10 or 0, so this value reflects count obfuscation rather than a measurable imbalance.

Baseline characteristics of patients initiating upadacitinib before 2021 versus contemporaneous tumour necrosis factor inhibitor (TNFi) initiators, before and after 1:1 propensity score matching. The propensity score model included demographics, cardiovascular risk factors and comorbidities, other autoimmune and inflammatory diagnoses, conventional synthetic DMARDs, corticosteroids, prior non-TNF biologic DMARDs, and baseline inflammatory markers. Values are mean ± standard deviation (SD) or median [interquartile range] for continuous variables and percentages for categorical variables. Standardised mean differences (SMD) < 0.1 indicate adequate balance. Upadacitinib was approved for rheumatoid arthritis in August 2019, so this stratum captures the immediate post-approval period, and its initiators were the most heavily pre-treated group in the study: before matching they differed from contemporaneous TNFi initiators most markedly in prior non-TNF biologic DMARD exposure (abatacept 21.7% versus 2.6%, SMD 0.613; tocilizumab 16.7% versus 1.5%, SMD 0.550; rituximab 7.5% versus 0.9%, SMD 0.332) and in corticosteroid and csDMARD exposure (methylprednisolone SMD 0.672; prednisone 0.573; leflunomide 0.424). After matching, all demographic, cardiovascular, csDMARD and corticosteroid SMDs were ≤ 0.043; excluding covariates affected by low-count obfuscation, the largest residual differences were for prior tocilizumab (12.4% versus 9.4%; SMD 0.097; *P* = 0.031), abatacept (0.070), erythrocyte sedimentation rate (0.066), rituximab (0.065), systemic lupus erythematosus (0.056) and cerebral infarction (0.052), each remaining more frequent in the upadacitinib arm. Of 1,060 eligible upadacitinib initiators, 68 could not be matched, and these were almost entirely biologic-experienced (48 had prior abatacept, 54 prior tocilizumab and 23 prior rituximab exposure), indicating that no TNFi comparator was available for the most refractory initiators; the matched cohort therefore under-represents this group. Baseline C-reactive protein was well balanced after matching (15.0 ± 27.7 versus 15.3 ± 29.9 mg/L; SMD 0.010). Because the residual imbalance in prior biologic exposure marks more refractory disease and runs in the direction of the upadacitinib arm, any residual confounding in this stratum would be expected to bias estimates toward harm. Interquartile range is reported as the width of the interquartile range. TNFi = tumour necrosis factor inhibitors; SD = standard deviation; SMD = standardised mean difference.

**Supplementary Table S9. Baseline characteristics in the post-2021 upadacitinib versus TNFi propensity score–matched cohort**

| **Characteristic** | **Before: Upadacitinib (n = 7,053)** | **Before: TNFi (n = 24,123)** | **SMD** | **After: Upadacitinib (n = 6,482)** | **After: TNFi (n = 6,482)** | **SMD** |
| --- | --- | --- | --- | --- | --- | --- |
| **Demographics** |  |  |  |  |  |  |
| Age, current, years (mean ± SD) | 65.2 ± 8.74 | 66.2 ± 9.28 | 0.1122 | 65.3 ± 8.75 | 65.5 ± 9.20 | 0.0184 |
| Age at index, years (mean ± SD) | 62.6 ± 8.74 | 63.4 ± 9.31 | 0.0949 | 62.7 ± 8.74 | 62.8 ± 9.25 | 0.0196 |
| Female, % | 80.52 | 74.75 | 0.1389 | 79.76 | 80.35 | 0.0147 |
| Male, % | 19.45 | 25.24 | 0.1393 | 20.21 | 19.62 | 0.0147 |
| Unknown gender, % | 0.14 | 0.04 | 0.0332 | 0.15 | 0.15 | < 0.0001 |
| White, % | 68.79 | 68.61 | 0.0040 | 68.98 | 68.05 | 0.0199 |
| Black or African American, % | 13.80 | 15.00 | 0.0344 | 13.93 | 14.66 | 0.0207 |
| Asian, % | 3.74 | 2.63 | 0.0633 | 3.44 | 3.55 | 0.0059 |
| American Indian or Alaska Native, % | 0.71 | 0.86 | 0.0169 | 0.76 | 0.77 | 0.0018 |
| Native Hawaiian or Other Pacific Islander, % | 0.27 | 0.29 | 0.0047 | 0.26 | 0.36 | 0.0167 |
| Other race, % | 2.20 | 3.54 | 0.0803 | 2.18 | 1.81 | 0.0265 |
| Unknown race, % | 10.49 | 9.07 | 0.0479 | 10.46 | 10.81 | 0.0115 |
| Hispanic or Latino, % | 8.61 | 7.54 | 0.0393 | 8.49 | 8.33 | 0.0056 |
| Not Hispanic or Latino, % | 67.84 | 65.99 | 0.0395 | 67.53 | 67.94 | 0.0089 |
| Unknown ethnicity, % | 23.55 | 26.48 | 0.0676 | 23.99 | 23.73 | 0.0062 |
| **Cardiovascular risk factors and comorbidities** |  |  |  |  |  |  |
| Hypertension, % | 66.40 | 67.74 | 0.0286 | 66.49 | 67.17 | 0.0144 |
| Dyslipidemia, % | 61.87 | 59.12 | 0.0564 | 61.43 | 62.82 | 0.0286 |
| Overweight and obesity, % | 35.22 | 33.80 | 0.0298 | 35.07 | 35.24 | 0.0036 |
| Type 2 diabetes, % | 24.70 | 26.02 | 0.0304 | 24.73 | 24.36 | 0.0086 |
| Nicotine dependence, % | 17.67 | 19.83 | 0.0555 | 17.97 | 17.26 | 0.0186 |
| Tobacco use, % | 6.83 | 7.59 | 0.0294 | 6.87 | 6.26 | 0.0243 |
| Ischaemic heart disease, % | 17.75 | 19.36 | 0.0413 | 17.97 | 17.73 | 0.0064 |
| Cerebral infarction, % | 3.30 | 4.24 | 0.0490 | 3.33 | 3.47 | 0.0077 |
| Heart failure, % | 7.43 | 7.15 | 0.0109 | 7.47 | 7.70 | 0.0087 |
| Atrial fibrillation, % | 5.98 | 7.20 | 0.0491 | 6.09 | 6.03 | 0.0026 |
| Chronic kidney disease, % | 10.86 | 10.40 | 0.0151 | 10.77 | 10.85 | 0.0025 |
| Acute kidney injury, % | 7.17 | 6.99 | 0.0072 | 7.19 | 7.25 | 0.0024 |
| **Other autoimmune and inflammatory diagnoses** |  |  |  |  |  |  |
| Systemic lupus erythematosus, % | 5.69 | 3.41 | 0.1093 | 5.38 | 5.37 | 0.0007 |
| Systemic sclerosis, % | 1.21 | 0.62 | 0.0619 | 1.06 | 1.02 | 0.0046 |
| Dermatomyositis, % | 1.12 | 0.36 | 0.0887 | 0.97 | 0.93 | 0.0048 |
| Antiphospholipid syndrome, % | 0.48 | 0.54 | 0.0074 | 0.48 | 0.40 | 0.0117 |
| Arthropathic psoriasis, % | 0.67 | 0.31 | 0.0517 | 0.53 | 0.62 | 0.0123 |
| Ankylosing spondylitis, % | 0.78 | 0.22 | 0.0802 | 0.49 | 0.51 | 0.0022 |
| Crohn's disease, % | 0.16 | 0.17 | 0.0035 | 0.17 | 0.15 | 0.0038 |
| Ulcerative colitis, % | 0.17 | 0.22 | 0.0121 | 0.19 | 0.20 | 0.0035 |
| **csDMARDs and corticosteroids** |  |  |  |  |  |  |
| Methotrexate, % | 52.79 | 51.46 | 0.0265 | 51.94 | 51.37 | 0.0114 |
| Hydroxychloroquine, % | 39.53 | 32.87 | 0.1388 | 38.28 | 38.60 | 0.0067 |
| Leflunomide, % | 26.94 | 17.15 | 0.2377 | 25.12 | 25.33 | 0.0050 |
| Sulfasalazine, % | 16.14 | 12.47 | 0.1048 | 15.37 | 14.66 | 0.0199 |
| Prednisone, % | 67.79 | 58.45 | 0.1945 | 65.92 | 66.45 | 0.0111 |
| Methylprednisolone, % | 54.57 | 39.71 | 0.3011 | 52.38 | 52.28 | 0.0019 |
| Dexamethasone, % | 37.77 | 30.81 | 0.1472 | 36.49 | 36.64 | 0.0032 |
| **Prior non-TNF biologic DMARDs** |  |  |  |  |  |  |
| Abatacept, % | 17.18 | 3.81 | 0.4469 | 12.56 | 12.57 | 0.0005 |
| Tocilizumab, % | 12.12 | 2.06 | 0.3998 | 7.81 | 7.28 | 0.0199 |
| Rituximab, % | 4.58 | 1.04 | 0.2154 | 3.27 | 3.02 | 0.0141 |
| **Laboratory parameters** |  |  |  |  |  |  |
| C-reactive protein, mg/L (mean ± SD) | 14.9 ± 30.1 | 16.2 ± 30.9 | 0.0428 | 15.2 ± 31.0 | 15.6 ± 29.3 | 0.0155 |
| C-reactive protein, mg/L (median [IQR]) | 6.0 [12.0] | 6.6 [13.2] | — | 6.0 [12.0] | 6.4 [13.3] | — |
| C-reactive protein recorded, % | 67.21 | 58.69 | 0.1771 | 65.75 | 66.32 | 0.0121 |
| Erythrocyte sedimentation rate, mm/h (mean ± SD) | 26.4 ± 24.4 | 27.6 ± 24.2 | 0.0491 | 26.5 ± 24.5 | 27.3 ± 24.6 | 0.0309 |
| Erythrocyte sedimentation rate, mm/h (median [IQR]) | 19 [28] | 20 [28] | — | 19 [28] | 19 [28] | — |
| Erythrocyte sedimentation rate recorded, % | 65.76 | 58.29 | 0.1543 | 64.61 | 65.18 | 0.0120 |

Baseline characteristics of patients initiating upadacitinib from 2021 onward versus contemporaneous tumour necrosis factor inhibitor (TNFi) initiators, before and after 1:1 propensity score matching. The propensity score model included demographics, cardiovascular risk factors and comorbidities, other autoimmune and inflammatory diagnoses, conventional synthetic DMARDs, corticosteroids, prior non-TNF biologic DMARDs, and baseline inflammatory markers. Values are mean ± standard deviation (SD) or median [interquartile range] for continuous variables and percentages for categorical variables. Standardised mean differences (SMD) < 0.1 indicate adequate balance. This is the largest agent-by-era stratum (6,482 pairs). Before matching, upadacitinib and TNFi initiators were closely comparable on cardiovascular comorbidity (hypertension 66.4% versus 67.7%, SMD 0.029; heart failure 7.4% versus 7.1%, SMD 0.011; acute kidney injury 7.2% versus 7.0%, SMD 0.007), with TNFi initiators carrying slightly more ischaemic heart disease (19.4% versus 17.8%) and atrial fibrillation (7.2% versus 6.0%). The dominant pre-matching differences were in prior non-TNF biologic DMARD exposure (abatacept 17.2% versus 3.8%, SMD 0.447; tocilizumab 12.1% versus 2.1%, SMD 0.400; rituximab 4.6% versus 1.0%, SMD 0.215) and in corticosteroid and csDMARD exposure (methylprednisolone SMD 0.301; leflunomide 0.238; prednisone 0.195). After matching, every SMD was ≤ 0.031 and prior biologic exposure was balanced to within 0.020 on all three agents, the most complete balance achieved in any stratum in this study; the largest residual differences were for erythrocyte sedimentation rate (0.031), dyslipidemia (0.029), other race (0.027) and tobacco use (0.024). Baseline C-reactive protein was well balanced (15.2 ± 31.0 versus 15.6 ± 29.3 mg/L; SMD 0.016). Of 7,053 eligible upadacitinib initiators, 571 could not be matched, of whom 398 had prior abatacept and 349 prior tocilizumab exposure. Interquartile range is reported as the width of the interquartile range. TNFi = tumour necrosis factor inhibitors; SD = standard deviation; SMD = standardised mean difference.

**Supplementary Table S10. Baseline characteristics in the pre-2021 all–JAK inhibitor versus TNFi propensity score–matched cohort**

| **Characteristic** | **Before: JAKi (n = 9,075)** | **Before: TNFi (n = 31,643)** | **SMD** | **After: JAKi (n = 8,437)** | **After: TNFi (n = 8,437)** | **SMD** |
| --- | --- | --- | --- | --- | --- | --- |
| **Demographics** |  |  |  |  |  |  |
| Age, current, years (mean ± SD) | 69.0 ± 9.21 | 69.7 ± 9.49 | 0.0844 | 69.0 ± 9.19 | 68.9 ± 9.48 | 0.0117 |
| Age at index, years (mean ± SD) | 61.1 ± 9.17 | 59.9 ± 9.38 | 0.1220 | 61.1 ± 9.15 | 61.1 ± 9.41 | 0.0040 |
| Female, % | 80.24 | 75.44 | 0.1159 | 79.79 | 80.22 | 0.0107 |
| Male, % | 19.76 | 24.56 | 0.1159 | 20.21 | 19.78 | 0.0107 |
| Unknown gender, % | 0 | 0 | — | 0 | 0 | — |
| White, % | 69.39 | 70.43 | 0.0228 | 69.30 | 70.49 | 0.0258 |
| Black or African American, % | 14.57 | 15.11 | 0.0152 | 14.69 | 14.37 | 0.0091 |
| Asian, % | 4.06 | 3.02 | 0.0560 | 3.98 | 3.57 | 0.0218 |
| American Indian or Alaska Native, % | 0.63 | 0.75 | 0.0146 | 0.65 | 0.55 | 0.0138 |
| Native Hawaiian or Other Pacific Islander, % | 0.30 | 0.27 | 0.0048 | 0.31 | 0.30 | 0.0022 |
| Other race, % | 2.34 | 2.72 | 0.0241 | 2.37 | 2.19 | 0.0119 |
| Unknown race, % | 8.73 | 7.71 | 0.0372 | 8.70 | 8.55 | 0.0055 |
| Hispanic or Latino, % | 6.88 | 7.84 | 0.0369 | 6.90 | 6.98 | 0.0033 |
| Not Hispanic or Latino, % | 69.72 | 69.88 | 0.0036 | 69.61 | 69.82 | 0.0046 |
| Unknown ethnicity, % | 23.41 | 22.28 | 0.0269 | 23.49 | 23.20 | 0.0070 |
| **Cardiovascular risk factors and comorbidities** |  |  |  |  |  |  |
| Hypertension, % | 66.11 | 63.36 | 0.0575 | 65.68 | 65.87 | 0.0040 |
| Dyslipidemia, % | 52.66 | 46.56 | 0.1223 | 52.21 | 51.43 | 0.0157 |
| Overweight and obesity, % | 27.35 | 23.29 | 0.0935 | 26.73 | 26.19 | 0.0121 |
| Type 2 diabetes, % | 23.74 | 22.53 | 0.0286 | 23.59 | 23.28 | 0.0073 |
| Nicotine dependence, % | 17.82 | 17.39 | 0.0114 | 17.64 | 17.60 | 0.0009 |
| Tobacco use, % | 6.02 | 4.99 | 0.0450 | 5.90 | 5.68 | 0.0096 |
| Ischaemic heart disease, % | 16.89 | 14.48 | 0.0663 | 16.67 | 16.43 | 0.0064 |
| Cerebral infarction, % | 3.46 | 2.99 | 0.0264 | 3.59 | 3.54 | 0.0026 |
| Heart failure, % | 7.44 | 4.58 | 0.1205 | 7.23 | 7.15 | 0.0032 |
| Atrial fibrillation, % | 6.44 | 4.70 | 0.0758 | 6.23 | 6.52 | 0.0116 |
| Chronic kidney disease, % | 7.71 | 5.83 | 0.0750 | 7.53 | 7.48 | 0.0018 |
| Acute kidney injury, % | 5.97 | 3.90 | 0.0960 | 5.74 | 5.52 | 0.0093 |
| **Other autoimmune and inflammatory diagnoses** |  |  |  |  |  |  |
| Systemic lupus erythematosus, % | 4.96 | 3.45 | 0.0753 | 4.54 | 4.23 | 0.0150 |
| Systemic sclerosis, % | 1.06 | 0.74 | 0.0334 | 0.97 | 0.89 | 0.0086 |
| Dermatomyositis, % | 0.69 | 0.40 | 0.0402 | 0.62 | 0.58 | 0.0046 |
| Antiphospholipid syndrome, % | 0.71 | 0.56 | 0.0180 | 0.70 | 0.65 | 0.0058 |
| Arthropathic psoriasis, % | 0.30 | 0.27 | 0.0061 | 0.30 | 0.27 | 0.0045 |
| Ankylosing spondylitis, % | 0.28 | 0.12 | 0.0341 | 0.25 | 0.20 | 0.0100 |
| Crohn's disease, % | 0.12 | 0.13 | 0.0015 | 0.12 | 0.14 | 0.0066 |
| Ulcerative colitis, % | 0.11 | 0.06 | 0.0172 | 0.12 | 0.12 | < 0.0001 |
| **csDMARDs and corticosteroids** |  |  |  |  |  |  |
| Methotrexate, % | 52.58 | 48.43 | 0.0832 | 51.91 | 51.52 | 0.0078 |
| Hydroxychloroquine, % | 35.75 | 27.79 | 0.1714 | 34.46 | 34.57 | 0.0025 |
| Leflunomide, % | 24.66 | 13.81 | 0.2780 | 23.03 | 22.50 | 0.0127 |
| Sulfasalazine, % | 17.17 | 12.86 | 0.1209 | 16.53 | 16.84 | 0.0083 |
| Prednisone, % | 63.96 | 50.74 | 0.2696 | 62.53 | 62.44 | 0.0020 |
| Methylprednisolone, % | 42.85 | 28.45 | 0.3041 | 40.87 | 40.97 | 0.0022 |
| Dexamethasone, % | 22.14 | 15.06 | 0.1827 | 21.28 | 21.47 | 0.0046 |
| **Prior non-TNF biologic DMARDs** |  |  |  |  |  |  |
| Abatacept, % | 12.87 | 2.57 | 0.3933 | 9.07 | 7.93 | 0.0408 |
| Tocilizumab, % | 8.82 | 1.45 | 0.3383 | 6.21 | 4.82 | 0.0608 |
| Rituximab, % | 4.45 | 0.89 | 0.2219 | 3.20 | 2.57 | 0.0375 |
| **Laboratory parameters** |  |  |  |  |  |  |
| C-reactive protein, mg/L (mean ± SD) | 15.6 ± 29.5 | 16.3 ± 31.1 | 0.0225 | 15.5 ± 29.1 | 16.4 ± 31.2 | 0.0322 |
| C-reactive protein, mg/L (median [IQR]) | 6.7 [12.9] | 7.0 [13.3] | — | 6.6 [13.0] | 6.9 [13.6] | — |
| C-reactive protein recorded, % | 61.29 | 51.35 | 0.2013 | 59.92 | 58.55 | 0.0277 |
| Erythrocyte sedimentation rate, mm/h (mean ± SD) | 28.2 ± 24.6 | 27.7 ± 24.4 | 0.0188 | 28.2 ± 24.5 | 28.4 ± 25.2 | 0.0091 |
| Erythrocyte sedimentation rate, mm/h (median [IQR]) | 21 [29] | 20 [28] | — | 21 [29] | 20 [29] | — |
| Erythrocyte sedimentation rate recorded, % | 61.26 | 54.59 | 0.1354 | 59.93 | 59.71 | 0.0044 |

Baseline characteristics of patients initiating any JAK inhibitor before 2021 versus contemporaneous tumour necrosis factor inhibitor (TNFi) initiators, before and after 1:1 propensity score matching. The propensity score model included demographics, cardiovascular risk factors and comorbidities, other autoimmune and inflammatory diagnoses, conventional synthetic DMARDs, corticosteroids, prior non-TNF biologic DMARDs, and baseline inflammatory markers. Values are mean ± standard deviation (SD) or median [interquartile range] for continuous variables and percentages for categorical variables. Standardised mean differences (SMD) < 0.1 indicate adequate balance. Before matching, pre-2021 JAK inhibitor initiators differed from contemporaneous TNFi initiators most markedly in prior non-TNF biologic DMARD exposure (abatacept 12.9% versus 2.6%, SMD 0.393; tocilizumab 8.8% versus 1.5%, SMD 0.338; rituximab 4.5% versus 0.9%, SMD 0.222) and in corticosteroid and csDMARD exposure (methylprednisolone SMD 0.304; leflunomide 0.278; prednisone 0.270); they were also older at index (61.1 versus 59.9 years) and had more heart failure (7.4% versus 4.6%) and acute kidney injury (6.0% versus 3.9%). After matching, all demographic, cardiovascular, csDMARD and corticosteroid SMDs were ≤ 0.026. The largest residual differences were for prior tocilizumab (6.2% versus 4.8%; SMD 0.061; *P* < 0.001), abatacept (0.041; *P* = 0.008), rituximab (0.038; *P* = 0.015) and baseline C-reactive protein (0.032), each remaining more frequent or higher in the JAK inhibitor arm. Of 9,075 eligible JAK inhibitor initiators, 638 could not be matched, of whom 403 had prior abatacept, 276 prior tocilizumab and 134 prior rituximab exposure, indicating that the unmatched remainder consisted almost entirely of biologic-experienced patients for whom no contemporaneous TNFi comparator was available. Baseline C-reactive protein (15.5 ± 29.1 versus 16.4 ± 31.2 mg/L) and erythrocyte sedimentation rate (SMD 0.009) were well balanced after matching. Interquartile range is reported as the width of the interquartile range. JAKi = Janus kinase inhibitors; TNFi = tumour necrosis factor inhibitors; SD = standard deviation; SMD = standardised mean difference.

**Supplementary Table S11. Baseline characteristics in the post-2021 all–JAK inhibitor versus TNFi propensity score–matched cohort**

| **Characteristic** | **Before: JAKi (n = 10,766)** | **Before: TNFi (n = 24,123)** | **SMD** | **After: JAKi (n = 10,230)** | **After: TNFi (n = 10,230)** | **SMD** |
| --- | --- | --- | --- | --- | --- | --- |
| **Demographics** |  |  |  |  |  |  |
| Age, current, years (mean ± SD) | 66.2 ± 9.09 | 66.2 ± 9.28 | 0.0014 | 66.2 ± 9.10 | 66.1 ± 9.29 | 0.0149 |
| Age at index, years (mean ± SD) | 63.2 ± 9.03 | 63.4 ± 9.31 | 0.0231 | 63.3 ± 9.05 | 63.1 ± 9.30 | 0.0158 |
| Female, % | 78.64 | 74.75 | 0.0921 | 78.29 | 78.50 | 0.0050 |
| Male, % | 21.34 | 25.24 | 0.0925 | 21.68 | 21.48 | 0.0050 |
| Unknown gender, % | 0.09 | 0.04 | 0.0199 | 0.10 | 0.10 | < 0.0001 |
| White, % | 65.32 | 68.61 | 0.0700 | 65.50 | 65.01 | 0.0105 |
| Black or African American, % | 13.61 | 15.00 | 0.0398 | 13.76 | 13.48 | 0.0083 |
| Asian, % | 4.06 | 2.63 | 0.0794 | 3.84 | 4.02 | 0.0091 |
| American Indian or Alaska Native, % | 0.73 | 0.86 | 0.0151 | 0.73 | 0.73 | < 0.0001 |
| Native Hawaiian or Other Pacific Islander, % | 0.23 | 0.29 | 0.0121 | 0.23 | 0.20 | 0.0064 |
| Other race, % | 2.56 | 3.54 | 0.0566 | 2.62 | 2.28 | 0.0221 |
| Unknown race, % | 13.50 | 9.07 | 0.1402 | 13.31 | 14.29 | 0.0283 |
| Hispanic or Latino, % | 7.51 | 7.54 | 0.0008 | 7.52 | 7.38 | 0.0052 |
| Not Hispanic or Latino, % | 64.89 | 65.99 | 0.0231 | 64.68 | 65.09 | 0.0086 |
| Unknown ethnicity, % | 27.60 | 26.48 | 0.0252 | 27.80 | 27.53 | 0.0061 |
| **Cardiovascular risk factors and comorbidities** |  |  |  |  |  |  |
| Hypertension, % | 66.63 | 67.74 | 0.0237 | 66.94 | 66.71 | 0.0050 |
| Dyslipidemia, % | 59.84 | 59.12 | 0.0146 | 59.75 | 59.98 | 0.0048 |
| Overweight and obesity, % | 33.41 | 33.80 | 0.0083 | 33.39 | 33.63 | 0.0050 |
| Type 2 diabetes, % | 26.13 | 26.02 | 0.0025 | 26.25 | 25.85 | 0.0091 |
| Nicotine dependence, % | 17.17 | 19.83 | 0.0685 | 17.40 | 16.81 | 0.0156 |
| Tobacco use, % | 6.34 | 7.59 | 0.0495 | 6.42 | 6.07 | 0.0145 |
| Ischaemic heart disease, % | 18.55 | 19.36 | 0.0206 | 18.58 | 17.91 | 0.0175 |
| Cerebral infarction, % | 3.60 | 4.24 | 0.0326 | 3.66 | 3.46 | 0.0106 |
| Heart failure, % | 8.82 | 7.15 | 0.0616 | 8.71 | 8.68 | 0.0010 |
| Atrial fibrillation, % | 6.84 | 7.20 | 0.0143 | 6.83 | 7.04 | 0.0081 |
| Chronic kidney disease, % | 11.00 | 10.40 | 0.0194 | 11.04 | 10.50 | 0.0173 |
| Acute kidney injury, % | 8.87 | 6.99 | 0.0697 | 8.77 | 8.89 | 0.0041 |
| **Other autoimmune and inflammatory diagnoses** |  |  |  |  |  |  |
| Systemic lupus erythematosus, % | 5.62 | 3.41 | 0.1065 | 5.14 | 5.22 | 0.0035 |
| Systemic sclerosis, % | 1.10 | 0.62 | 0.0519 | 1.01 | 0.85 | 0.0163 |
| Dermatomyositis, % | 1.37 | 0.36 | 0.1088 | 0.96 | 0.83 | 0.0135 |
| Antiphospholipid syndrome, % | 0.45 | 0.54 | 0.0127 | 0.44 | 0.45 | 0.0015 |
| Arthropathic psoriasis, % | 0.51 | 0.31 | 0.0320 | 0.50 | 0.53 | 0.0041 |
| Ankylosing spondylitis, % | 0.46 | 0.22 | 0.0428 | 0.42 | 0.37 | 0.0078 |
| Crohn's disease, % | 0.19 | 0.17 | 0.0038 | 0.20 | 0.15 | 0.0118 |
| Ulcerative colitis, % | 0.16 | 0.22 | 0.0151 | 0.17 | 0.16 | 0.0024 |
| **csDMARDs and corticosteroids** |  |  |  |  |  |  |
| Methotrexate, % | 47.07 | 51.46 | 0.0878 | 46.75 | 45.69 | 0.0212 |
| Hydroxychloroquine, % | 35.03 | 32.87 | 0.0455 | 34.33 | 34.31 | 0.0004 |
| Leflunomide, % | 21.74 | 17.15 | 0.1162 | 20.70 | 21.50 | 0.0194 |
| Sulfasalazine, % | 14.92 | 12.47 | 0.0713 | 14.46 | 14.49 | 0.0008 |
| Prednisone, % | 58.41 | 58.45 | 0.0008 | 57.54 | 57.28 | 0.0051 |
| Methylprednisolone, % | 45.52 | 39.71 | 0.1178 | 44.13 | 44.41 | 0.0057 |
| Dexamethasone, % | 34.92 | 30.81 | 0.0876 | 34.21 | 34.06 | 0.0033 |
| **Prior non-TNF biologic DMARDs** |  |  |  |  |  |  |
| Abatacept, % | 11.56 | 3.81 | 0.2940 | 9.01 | 8.64 | 0.0131 |
| Tocilizumab, % | 7.61 | 2.06 | 0.2608 | 5.54 | 4.78 | 0.0345 |
| Rituximab, % | 3.34 | 1.04 | 0.1578 | 2.55 | 2.31 | 0.0159 |
| **Laboratory parameters** |  |  |  |  |  |  |
| C-reactive protein, mg/L (mean ± SD) | 18.7 ± 36.9 | 16.2 ± 30.9 | 0.0733 | 19.0 ± 37.5 | 16.0 ± 30.1 | 0.0897 |
| C-reactive protein, mg/L (median [IQR]) | 6.0 [14.4] | 6.6 [13.2] | — | 6.1 [14.5] | 6.4 [13.3] | — |
| C-reactive protein recorded, % | 61.37 | 58.69 | 0.0548 | 60.63 | 60.13 | 0.0102 |
| Erythrocyte sedimentation rate, mm/h (mean ± SD) | 27.7 ± 25.0 | 27.6 ± 24.2 | 0.0047 | 28.0 ± 25.2 | 27.6 ± 24.1 | 0.0167 |
| Erythrocyte sedimentation rate, mm/h (median [IQR]) | 20 [30] | 20 [28] | — | 20 [30] | 20 [28] | — |
| Erythrocyte sedimentation rate recorded, % | 58.33 | 58.29 | 0.0008 | 57.93 | 57.62 | 0.0063 |

Baseline characteristics of patients initiating any JAK inhibitor from 2021 onward versus contemporaneous tumour necrosis factor inhibitor (TNFi) initiators, before and after 1:1 propensity score matching. The propensity score model included demographics, cardiovascular risk factors and comorbidities, other autoimmune and inflammatory diagnoses, conventional synthetic DMARDs, corticosteroids, prior non-TNF biologic DMARDs, and baseline inflammatory markers. Values are mean ± standard deviation (SD) or median [interquartile range] for continuous variables and percentages for categorical variables. Standardised mean differences (SMD) < 0.1 indicate adequate balance. In marked contrast to the pre-2021 stratum, post-2021 JAK inhibitor and TNFi initiators were closely comparable before matching on age (66.2 versus 66.2 years; SMD 0.001), hypertension (66.6% versus 67.7%; 0.024), dyslipidemia (59.8% versus 59.1%; 0.015), type 2 diabetes (26.1% versus 26.0%; 0.003) and prednisone exposure (58.4% versus 58.4%; 0.001), indicating that channeling by cardiovascular risk and corticosteroid burden had largely resolved in the later era. Channeling by line of therapy persisted, although at reduced magnitude relative to the pre-2021 stratum: prior non-TNF biologic DMARD exposure remained the dominant pre-matching difference (abatacept 11.6% versus 3.8%, SMD 0.294, versus 0.393 pre-2021; tocilizumab 0.261; rituximab 0.158). After matching, prior biologic exposure was well balanced (abatacept 0.013; rituximab 0.016) and all demographic, cardiovascular, csDMARD and corticosteroid SMDs were ≤ 0.028. The largest residual difference was for baseline C-reactive protein (19.0 ± 37.5 versus 16.0 ± 30.1 mg/L; SMD 0.090; *P* < 0.001), which was not reduced by matching (pre-matching SMD 0.073); prior tocilizumab exposure (0.035; *P* = 0.014), unknown race (0.028), other race (0.022) and methotrexate (0.021) followed. Because the pooled cohort was matched on a single propensity score across all three agents rather than as the union of separately matched agent-specific cohorts, patients with markedly elevated inflammatory markers were retained but incompletely balanced; erythrocyte sedimentation rate was balanced (SMD 0.017). Estimates from this stratum are therefore subject to residual confounding by baseline inflammatory burden. Interquartile range is reported as the width of the interquartile range. JAKi = Janus kinase inhibitors; TNFi = tumour necrosis factor inhibitors; SD = standard deviation; SMD = standardised mean difference.

**Supplementary Table S12. Baseline characteristics in the female JAK inhibitor versus female TNF inhibitor propensity score–matched cohort**

| **Characteristic** | **Before: JAKi (n = 16,152)** | **Before: TNFi (n = 43,018)** | **SMD** | **After: JAKi (n = 15,551)** | **After: TNFi (n = 15,551)** | **SMD** |
| --- | --- | --- | --- | --- | --- | --- |
| **Demographics** |  |  |  |  |  |  |
| Age, current, years (mean ± SD) | 67.2 ± 9.29 | 68.0 ± 9.66 | 0.0843 | 67.3 ± 9.31 | 67.1 ± 9.55 | 0.0216 |
| Age at index, years (mean ± SD) | 62.1 ± 9.21 | 61.4 ± 9.60 | 0.0700 | 62.1 ± 9.23 | 62.2 ± 9.58 | 0.0164 |
| Female, % | 100 | 100 | – | 100 | 100 | – |
| Male, % | 0 | 0 | – | 0 | 0 | – |
| Unknown gender, % | 0 | 0 | – | 0 | 0 | – |
| White, % | 65.18 | 67.30 | 0.0448 | 65.33 | 65.29 | 0.0008 |
| Black or African American, % | 15.42 | 16.59 | 0.0319 | 15.52 | 15.61 | 0.0025 |
| Asian, % | 4.25 | 3.07 | 0.0628 | 4.09 | 4.17 | 0.0042 |
| American Indian or Alaska Native, % | 0.76 | 0.90 | 0.0154 | 0.77 | 0.73 | 0.0045 |
| Native Hawaiian or Other Pacific Islander, % | 0.27 | 0.29 | 0.0051 | 0.28 | 0.27 | 0.0012 |
| Other race, % | 2.57 | 3.28 | 0.0422 | 2.58 | 2.26 | 0.0209 |
| Unknown race, % | 11.56 | 8.58 | 0.0993 | 11.44 | 11.67 | 0.0072 |
| Hispanic or Latino, % | 7.85 | 8.40 | 0.0200 | 7.88 | 7.50 | 0.0142 |
| Not Hispanic or Latino, % | 66.13 | 67.05 | 0.0194 | 66.03 | 66.07 | 0.0007 |
| Unknown ethnicity, % | 26.02 | 24.55 | 0.0337 | 26.08 | 26.43 | 0.0079 |
| **Cardiovascular risk factors and comorbidities** |  |  |  |  |  |  |
| Hypertension, % | 65.05 | 64.16 | 0.0186 | 64.96 | 65.35 | 0.0082 |
| Dyslipidemia, % | 55.36 | 50.50 | 0.0976 | 55.14 | 55.78 | 0.0129 |
| Overweight and obesity, % | 31.73 | 29.51 | 0.0483 | 31.63 | 32.30 | 0.0143 |
| Type 2 diabetes, % | 23.97 | 23.27 | 0.0165 | 23.90 | 23.75 | 0.0035 |
| Nicotine dependence, % | 15.44 | 16.42 | 0.0267 | 15.54 | 15.63 | 0.0025 |
| Tobacco use, % | 5.37 | 5.43 | 0.0026 | 5.40 | 5.64 | 0.0107 |
| Ischaemic heart disease, % | 15.23 | 13.76 | 0.0417 | 15.09 | 14.99 | 0.0029 |
| Cerebral infarction, % | 3.34 | 3.46 | 0.0064 | 3.39 | 3.40 | 0.0004 |
| Heart failure, % | 7.42 | 5.21 | 0.0910 | 7.30 | 7.62 | 0.0122 |
| Atrial fibrillation, % | 5.53 | 4.66 | 0.0395 | 5.58 | 5.82 | 0.0105 |
| Chronic kidney disease, % | 8.95 | 7.46 | 0.0545 | 8.88 | 9.07 | 0.0067 |
| Acute kidney injury, % | 6.89 | 4.83 | 0.0878 | 6.80 | 7.00 | 0.0079 |
| **Other autoimmune and inflammatory diagnoses** |  |  |  |  |  |  |
| Systemic lupus erythematosus, % | 6.04 | 4.12 | 0.0873 | 5.78 | 5.56 | 0.0097 |
| Systemic sclerosis, % | 1.21 | 0.78 | 0.0435 | 1.16 | 1.07 | 0.0086 |
| Dermatomyositis, % | 1.06 | 0.40 | 0.0778 | 0.93 | 0.81 | 0.0125 |
| Antiphospholipid syndrome, % | 0.61 | 0.60 | 0.0011 | 0.59 | 0.53 | 0.0086 |
| Arthropathic psoriasis, % | 0.42 | 0.24 | 0.0303 | 0.38 | 0.39 | 0.0010 |
| Ankylosing spondylitis, % | 0.38 | 0.17 | 0.0398 | 0.34 | 0.27 | 0.0128 |
| Crohn's disease, % | 0.17 | 0.14 | 0.0077 | 0.17 | 0.18 | 0.0031 |
| Ulcerative colitis, % | 0.12 | 0.12 | 0.0015 | 0.12 | 0.12 | < 0.0001 |
| **csDMARDs and corticosteroids** |  |  |  |  |  |  |
| Methotrexate, % | 50.41 | 51.21 | 0.0161 | 50.04 | 50.11 | 0.0015 |
| Hydroxychloroquine, % | 37.07 | 32.03 | 0.1060 | 36.46 | 37.02 | 0.0116 |
| Leflunomide, % | 24.26 | 16.71 | 0.1877 | 23.45 | 23.54 | 0.0021 |
| Sulfasalazine, % | 15.84 | 12.74 | 0.0889 | 15.47 | 15.56 | 0.0025 |
| Prednisone, % | 61.29 | 54.44 | 0.1389 | 60.58 | 60.80 | 0.0046 |
| Methylprednisolone, % | 45.68 | 35.05 | 0.2180 | 44.55 | 45.44 | 0.0180 |
| Dexamethasone, % | 30.09 | 22.88 | 0.1640 | 29.43 | 30.07 | 0.0139 |
| **Prior non-TNF biologic DMARDs** |  |  |  |  |  |  |
| Abatacept, % | 13.05 | 3.82 | 0.3367 | 11.02 | 9.56 | 0.0481 |
| Tocilizumab, % | 8.53 | 2.09 | 0.2898 | 6.94 | 5.43 | 0.0625 |
| Rituximab, % | 3.98 | 1.10 | 0.1843 | 3.34 | 2.62 | 0.0424 |
| **Laboratory parameters** |  |  |  |  |  |  |
| C-reactive protein, mg/L (mean ± SD) | 16.0 ± 31.2 | 15.1 ± 29.3 | 0.0293 | 16.2 ± 31.6 | 14.7 ± 28.7 | 0.0504 |
| C-reactive protein, mg/L (median [IQR]) | 6.0 [12.9] | 6.4 [12.5] | — | 6.1 [12.8] | 6.0 [12.0] | — |
| C-reactive protein recorded, % | 62.16 | 55.47 | 0.1362 | 61.42 | 61.78 | 0.0074 |
| Erythrocyte sedimentation rate, mm/h (mean ± SD) | 28.7 ± 24.8 | 28.5 ± 24.1 | 0.0096 | 28.9 ± 24.8 | 28.3 ± 24.4 | 0.0229 |
| Erythrocyte sedimentation rate, mm/h (median [IQR]) | 21 [30] | 21 [28] | — | 21 [29] | 21 [28] | — |
| Erythrocyte sedimentation rate recorded, % | 60.78 | 57.26 | 0.0717 | 60.20 | 60.14 | 0.0012 |

Baseline characteristics of female patients initiating a JAK inhibitor versus female patients initiating a tumour necrosis factor inhibitor (TNFi), before and after 1:1 propensity score matching. The analysis was restricted to female patients, so sex is constant within this stratum and no standardised mean difference is defined for it. The propensity score model included demographics, cardiovascular risk factors and comorbidities, other autoimmune and inflammatory diagnoses, conventional synthetic DMARDs, corticosteroids, prior non-TNF biologic DMARDs, and baseline inflammatory markers. Values are mean ± standard deviation (SD) or median [interquartile range] for continuous variables and percentages for categorical variables. Standardised mean differences (SMD) < 0.1 indicate adequate balance. Before matching, female JAK inhibitor initiators differed from female TNFi initiators most markedly in prior non-TNF biologic DMARD exposure (abatacept 13.0% versus 3.8%, SMD 0.337; tocilizumab 8.5% versus 2.1%, SMD 0.290; rituximab 4.0% versus 1.1%, SMD 0.184) and in corticosteroid and csDMARD exposure (methylprednisolone SMD 0.218; leflunomide 0.188; dexamethasone 0.164; prednisone 0.139); they also had more heart failure (7.4% versus 5.2%) and acute kidney injury (6.9% versus 4.8%). After matching, all demographic, cardiovascular, csDMARD and corticosteroid SMDs were ≤ 0.022. The largest residual differences were for prior tocilizumab exposure (6.9% versus 5.4%; SMD 0.063; *P* < 0.001), baseline C-reactive protein (16.2 ± 31.6 versus 14.7 ± 28.7 mg/L; SMD 0.050; *P* < 0.001), abatacept (0.048; *P* < 0.001) and rituximab (0.042; *P* < 0.001), each remaining more frequent or higher in the JAK inhibitor arm. Matching did not reduce the difference in C-reactive protein (pre-matching SMD 0.029), and erythrocyte sedimentation rate showed a residual difference in the same direction (SMD 0.023). Estimates derived from this stratum are therefore subject to residual confounding by prior treatment line and baseline inflammatory burden, both of which mark more refractory disease and would be expected to bias estimates toward harm. Interquartile range is reported as the width of the interquartile range. JAKi = Janus kinase inhibitors; TNFi = tumour necrosis factor inhibitors; SD = standard deviation; SMD = standardised mean difference.

**Supplementary Table S13.** Baseline characteristics in the male JAK inhibitor versus male TNF inhibitor propensity score–matched cohort

| **Characteristic** | **Before: JAKi (n = 4,133)** | **Before: TNFi (n = 14,056)** | **SMD** | **After: JAKi (n = 3,983)** | **After: TNFi (n = 3,983)** | **SMD** |
| --- | --- | --- | --- | --- | --- | --- |
| **Demographics** |  |  |  |  |  |  |
| Age, current, years (mean ± SD) | 67.9 ± 8.97 | 68.5 ± 9.23 | 0.0650 | 68.0 ± 8.98 | 67.8 ± 9.03 | 0.0195 |
| Age at index, years (mean ± SD) | 62.9 ± 8.84 | 62.0 ± 9.18 | 0.0934 | 62.9 ± 8.86 | 63.0 ± 9.01 | 0.0207 |
| Male, % | 100 | 100 | – | 100 | 100 | – |
| Female, % | 0 | 0 | – | 0 | 0 | – |
| Unknown gender, % | 0 | 0 | – | 0 | 0 | – |
| White, % | 74.72 | 76.81 | 0.0488 | 74.79 | 75.42 | 0.0145 |
| Black or African American, % | 7.91 | 9.60 | 0.0597 | 7.98 | 7.76 | 0.0084 |
| Asian, % | 3.75 | 2.34 | 0.0821 | 3.72 | 3.24 | 0.0260 |
| American Indian or Alaska Native, % | 0.41 | 0.64 | 0.0317 | 0.43 | 0.63 | 0.0277 |
| Native Hawaiian or Other Pacific Islander, % | 0.24 | 0.23 | 0.0030 | 0.25 | 0.25 | < 0.0001 |
| Other race, % | 1.89 | 2.60 | 0.0484 | 1.93 | 1.83 | 0.0074 |
| Unknown race, % | 11.11 | 7.78 | 0.1138 | 10.92 | 10.90 | 0.0008 |
| Hispanic or Latino, % | 4.69 | 5.68 | 0.0444 | 4.65 | 4.67 | 0.0012 |
| Not Hispanic or Latino, % | 69.51 | 70.35 | 0.0182 | 69.42 | 69.85 | 0.0093 |
| Unknown ethnicity, % | 25.79 | 23.98 | 0.0420 | 25.94 | 25.48 | 0.0103 |
| **Cardiovascular risk factors and comorbidities** |  |  |  |  |  |  |
| Hypertension, % | 67.75 | 66.24 | 0.0322 | 67.81 | 67.39 | 0.0091 |
| Dyslipidemia, % | 59.62 | 54.90 | 0.0956 | 59.28 | 60.43 | 0.0236 |
| Overweight and obesity, % | 24.87 | 21.71 | 0.0750 | 24.43 | 25.18 | 0.0174 |
| Type 2 diabetes, % | 26.59 | 24.80 | 0.0410 | 26.31 | 26.61 | 0.0068 |
| Nicotine dependence, % | 23.40 | 23.29 | 0.0026 | 23.15 | 23.22 | 0.0018 |
| Tobacco use, % | 8.49 | 7.80 | 0.0254 | 8.41 | 8.69 | 0.0099 |
| Ischaemic heart disease, % | 25.62 | 24.52 | 0.0255 | 25.66 | 25.58 | 0.0017 |
| Cerebral infarction, % | 4.02 | 3.61 | 0.0214 | 3.94 | 4.19 | 0.0127 |
| Heart failure, % | 10.43 | 6.83 | 0.1284 | 10.24 | 10.47 | 0.0074 |
| Atrial fibrillation, % | 10.57 | 9.19 | 0.0463 | 10.44 | 10.12 | 0.0107 |
| Chronic kidney disease, % | 10.86 | 8.64 | 0.0751 | 10.75 | 10.50 | 0.0081 |
| Acute kidney injury, % | 9.15 | 6.40 | 0.1029 | 8.84 | 9.21 | 0.0131 |
| **Other autoimmune and inflammatory diagnoses** |  |  |  |  |  |  |
| Systemic lupus erythematosus, % | 2.03 | 1.21 | 0.0652 | 1.96 | 1.68 | 0.0207 |
| Systemic sclerosis, % | 0.58 | 0.33 | 0.0365 | 0.55 | 0.50 | 0.0069 |
| Dermatomyositis, % | 0.94 | 0.33 | 0.0765 | 0.75 | 0.75 | < 0.0001 |
| Antiphospholipid syndrome, % | 0.36 | 0.33 | 0.0048 | 0.35 | 0.33 | 0.0043 |
| Arthropathic psoriasis, % | 0.34 | 0.36 | 0.0029 | 0.33 | 0.43 | 0.0164 |
| Ankylosing spondylitis, % | 0.29 | 0.16 | 0.0283 | 0.25 | 0.28 | 0.0049 |
| Crohn's disease, % | 0.24 | 0.13 | 0.0265 | 0.25 | 0.25 | < 0.0001 |
| Ulcerative colitis, % | 0.24 | 0.13 | 0.0265 | 0.25 | 0.25 | < 0.0001 |
| **csDMARDs and corticosteroids** |  |  |  |  |  |  |
| Methotrexate, % | 47.74 | 47.97 | 0.0046 | 47.48 | 47.38 | 0.0020 |
| Hydroxychloroquine, % | 29.86 | 25.09 | 0.1069 | 29.35 | 29.45 | 0.0022 |
| Leflunomide, % | 18.99 | 12.05 | 0.1928 | 18.20 | 17.88 | 0.0085 |
| Sulfasalazine, % | 16.72 | 13.23 | 0.0978 | 16.37 | 16.27 | 0.0027 |
| Prednisone, % | 59.88 | 54.62 | 0.1066 | 59.08 | 60.93 | 0.0379 |
| Methylprednisolone, % | 40.50 | 30.29 | 0.2147 | 39.54 | 39.64 | 0.0021 |
| Dexamethasone, % | 26.18 | 20.37 | 0.1378 | 25.56 | 26.14 | 0.0132 |
| **Prior non-TNF biologic DMARDs** |  |  |  |  |  |  |
| Abatacept, % | 9.32 | 2.19 | 0.3096 | 7.68 | 6.85 | 0.0319 |
| Tocilizumab, % | 7.43 | 1.34 | 0.3009 | 5.67 | 4.39 | **0.0586** |
| Rituximab, % | 3.29 | 0.81 | 0.1756 | 2.69 | 2.26 | 0.0275 |
| **Laboratory parameters** |  |  |  |  |  |  |
| C-reactive protein, mg/L (mean ± SD) | 20.8 ± 40.0 | 18.9 ± 35.6 | 0.0505 | 20.6 ± 39.0 | 20.3 ± 38.8 | 0.0066 |
| C-reactive protein, mg/L (median [IQR]) | 7.0 [15.7] | 7.0 [15.3] | — | 7.0 [15.7] | 7.1 [16.7] | — |
| C-reactive protein recorded, % | 59.74 | 53.76 | 0.1210 | 59.13 | 59.28 | 0.0031 |
| Erythrocyte sedimentation rate, mm/h (mean ± SD) | 23.4 ± 23.8 | 24.0 ± 24.2 | 0.0248 | 23.5 ± 23.7 | 24.9 ± 25.0 | **0.0575** |
| Erythrocyte sedimentation rate, mm/h (median [IQR]) | 15 [25] | 15 [26] | — | 15 [25] | 16 [27] | — |
| Erythrocyte sedimentation rate recorded, % | 56.88 | 54.34 | 0.0512 | 56.24 | 56.57 | 0.0066 |

Baseline characteristics of male patients initiating a JAK inhibitor versus male patients initiating a tumour necrosis factor inhibitor (TNFi), before and after 1:1 propensity score matching. The analysis was restricted to male patients, so sex is constant within this stratum and no standardised mean difference is defined for it. The propensity score model included demographics, cardiovascular risk factors and comorbidities, other autoimmune and inflammatory diagnoses, conventional synthetic DMARDs, corticosteroids, prior non-TNF biologic DMARDs, and baseline inflammatory markers. Values are mean ± standard deviation (SD) or median [interquartile range] for continuous variables and percentages for categorical variables. Standardised mean differences (SMD) < 0.1 indicate adequate balance. Before matching, male JAK inhibitor initiators differed from male TNFi initiators most markedly in prior non-TNF biologic DMARD exposure (abatacept 9.3% versus 2.2%, SMD 0.310; tocilizumab 7.4% versus 1.3%, SMD 0.301; rituximab 3.3% versus 0.8%, SMD 0.176) and in corticosteroid and csDMARD exposure (methylprednisolone SMD 0.215; leflunomide 0.193; dexamethasone 0.138; prednisone 0.107); they also had more heart failure (10.4% versus 6.8%), acute kidney injury (9.1% versus 6.4%) and chronic kidney disease (10.9% versus 8.6%). After matching, every SMD was < 0.06. The largest residual differences were for prior tocilizumab exposure (5.7% versus 4.4%; SMD 0.059; *P* = 0.009), erythrocyte sedimentation rate (23.5 ± 23.7 versus 24.9 ± 25.0 mm/h; SMD 0.058), prednisone (0.038), abatacept (0.032), American Indian or Alaska Native race (0.028) and rituximab (0.028); all cardiovascular and renal comorbidities were balanced to ≤ 0.024. Baseline C-reactive protein was well balanced after matching (20.6 ± 39.0 versus 20.3 ± 38.8 mg/L; SMD 0.007), in contrast to the female stratum, where a residual difference in favour of the JAK inhibitor arm persisted. Male initiators of both treatments carried a substantially higher burden of cardiovascular and renal comorbidity and higher baseline C-reactive protein than their female counterparts. Interquartile range is reported as the width of the interquartile range. JAKi = Janus kinase inhibitors; TNFi = tumour necrosis factor inhibitors; SD = standard deviation; SMD = standardised mean difference.

**Supplementary Table S14. Baseline characteristics in the ASCVD-free, one–risk-factor JAK inhibitor versus TNF inhibitor propensity score–matched cohort**

| **Characteristic** | **Before: JAKi (n = 6,102)** | **Before: TNFi (n = 17,115)** | **SMD** | **After: JAKi (n = 5,880)** | **After: TNFi (n = 5,880)** | **SMD** |
| --- | --- | --- | --- | --- | --- | --- |
| **Demographics** |  |  |  |  |  |  |
| Age, current, years (mean ± SD) | 65.6 ± 8.95 | 66.6 ± 9.37 | 0.1006 | 65.7 ± 8.96 | 65.6 ± 9.06 | 0.0179 |
| Age at index, years (mean ± SD) | 60.7 ± 8.82 | 60.0 ± 9.27 | 0.0735 | 60.7 ± 8.83 | 60.9 ± 9.02 | 0.0170 |
| Female, % | 81.66 | 78.56 | 0.0778 | 81.31 | 81.53 | 0.0057 |
| Male, % | 18.34 | 21.44 | 0.0778 | 18.69 | 18.47 | 0.0057 |
| Unknown gender, % | 0 | 0 | — | 0 | 0 | — |
| White, % | 66.49 | 69.54 | 0.0654 | 66.46 | 66.63 | 0.0036 |
| Black or African American, % | 10.64 | 12.27 | 0.0513 | 10.77 | 10.82 | 0.0016 |
| Asian, % | 5.08 | 3.20 | 0.0947 | 4.98 | 4.69 | 0.0135 |
| American Indian or Alaska Native, % | 0.74 | 0.90 | 0.0180 | 0.77 | 0.80 | 0.0039 |
| Native Hawaiian or Other Pacific Islander, % | 0.18 | 0.25 | 0.0153 | 0.19 | 0.19 | < 0.0001 |
| Other race, % | 2.57 | 3.36 | 0.0464 | 2.62 | 2.57 | 0.0032 |
| Unknown race, % | 14.31 | 10.49 | 0.1161 | 14.22 | 14.30 | 0.0024 |
| Hispanic or Latino, % | 6.92 | 7.84 | 0.0354 | 6.91 | 7.31 | 0.0159 |
| Not Hispanic or Latino, % | 63.98 | 64.88 | 0.0188 | 64.15 | 63.64 | 0.0106 |
| Unknown ethnicity, % | 29.11 | 27.28 | 0.0406 | 28.95 | 29.05 | 0.0022 |
| **Cardiovascular risk factors and comorbidities** |  |  |  |  |  |  |
| Hypertension, % | 44.53 | 48.18 | 0.0733 | 44.71 | 44.22 | 0.0099 |
| Dyslipidemia, % | 34.43 | 28.34 | 0.1316 | 34.03 | 34.30 | 0.0057 |
| Overweight and obesity, % | 18.45 | 16.83 | 0.0427 | 18.38 | 19.05 | 0.0170 |
| Type 2 diabetes, % | 7.24 | 7.41 | 0.0063 | 7.25 | 7.18 | 0.0026 |
| Nicotine dependence, % | 11.87 | 13.87 | 0.0598 | 12.04 | 12.28 | 0.0073 |
| Tobacco use, % | 3.15 | 3.53 | 0.0213 | 3.13 | 3.03 | 0.0059 |
| Ischaemic heart disease, % | 1.25 | 0.98 | 0.0252 | 1.22 | 1.22 | < 0.0001 |
| Cerebral infarction, % | 0.33 | 0.18 | 0.0291 | 0.32 | 0.27 | 0.0094 |
| Heart failure, % | 2.15 | 1.51 | 0.0478 | 2.08 | 2.18 | 0.0071 |
| Atrial fibrillation, % | 3.05 | 2.66 | 0.0230 | 3.04 | 3.28 | 0.0136 |
| Chronic kidney disease, % | 3.80 | 3.74 | 0.0033 | 3.83 | 3.62 | 0.0108 |
| Acute kidney injury, % | 2.51 | 2.05 | 0.0310 | 2.47 | 2.48 | 0.0011 |
| **Other autoimmune and inflammatory diagnoses** |  |  |  |  |  |  |
| Systemic lupus erythematosus, % | 4.38 | 2.68 | 0.0923 | 4.07 | 3.81 | 0.0131 |
| Systemic sclerosis, % | 0.56 | 0.36 | 0.0288 | 0.53 | 0.46 | 0.0097 |
| Dermatomyositis, % | 0.82 | 0.33 | 0.0643 | 0.73 | 0.65 | 0.0103 |
| Antiphospholipid syndrome, % | 0.49 | 0.41 | 0.0123 | 0.46 | 0.44 | 0.0025 |
| Arthropathic psoriasis, % | 0.41 | 0.31 | 0.0167 | 0.39 | 0.44 | 0.0079 |
| Ankylosing spondylitis, % | 0.16 | 0.22 | 0.0133 | 0.17 | 0.17 | < 0.0001 |
| Crohn's disease, % | 0.16 | 0.09 | 0.0215 | 0.17 | 0.17 | < 0.0001 |
| Ulcerative colitis, % | 0.16 | 0.11 | 0.0160 | 0.17 | 0.17 | < 0.0001 |
| **csDMARDs and corticosteroids** |  |  |  |  |  |  |
| Methotrexate, % | 51.62 | 51.00 | 0.0124 | 51.26 | 50.41 | 0.0170 |
| Hydroxychloroquine, % | 35.81 | 29.10 | 0.1436 | 34.92 | 35.32 | 0.0086 |
| Leflunomide, % | 23.12 | 15.17 | 0.2032 | 22.19 | 22.25 | 0.0012 |
| Sulfasalazine, % | 16.59 | 13.57 | 0.0844 | 16.29 | 16.36 | 0.0018 |
| Prednisone, % | 57.03 | 51.36 | 0.1139 | 56.24 | 57.21 | 0.0196 |
| Methylprednisolone, % | 40.20 | 29.50 | 0.2261 | 38.95 | 38.98 | 0.0007 |
| Dexamethasone, % | 22.91 | 18.27 | 0.1151 | 22.43 | 22.93 | 0.0118 |
| **Prior non-TNF biologic DMARDs** |  |  |  |  |  |  |
| Abatacept, % | 11.91 | 3.39 | 0.3249 | 9.83 | 8.52 | 0.0454 |
| Tocilizumab, % | 7.90 | 1.93 | 0.2786 | 6.50 | 5.17 | 0.0566 |
| Rituximab, % | 3.29 | 0.98 | 0.1609 | 2.89 | 2.35 | 0.0341 |
| **Laboratory parameters** |  |  |  |  |  |  |
| C-reactive protein, mg/L (mean ± SD) | 14.0 ± 27.3 | 14.7 ± 29.2 | 0.0251 | 14.1 ± 27.5 | 15.2 ± 30.5 | 0.0351 |
| C-reactive protein, mg/L (median [IQR]) | 5.8 [11.6] | 6.0 [12.3] | — | 5.8 [11.7] | 5.8 [12.0] | — |
| C-reactive protein recorded, % | 58.62 | 52.69 | 0.1196 | 58.01 | 58.21 | 0.0041 |
| Erythrocyte sedimentation rate, mm/h (mean ± SD) | 25.2 ± 23.4 | 25.3 ± 22.8 | 0.0025 | 25.3 ± 23.3 | 25.7 ± 23.3 | 0.0189 |
| Erythrocyte sedimentation rate, mm/h (median [IQR]) | 18 [26] | 18 [25] | — | 18 [26] | 19 [26] | — |
| Erythrocyte sedimentation rate recorded, % | 57.28 | 53.60 | 0.0741 | 56.77 | 56.82 | 0.0010 |

Baseline characteristics of patients without established atherosclerotic cardiovascular disease and with exactly one baseline cardiovascular risk factor, initiating a JAK inhibitor versus a tumour necrosis factor inhibitor (TNFi), before and after 1:1 propensity score matching. The propensity score model included demographics, cardiovascular risk factors and comorbidities, other autoimmune and inflammatory diagnoses, conventional synthetic DMARDs, corticosteroids, prior non-TNF biologic DMARDs, and baseline inflammatory markers. Values are mean ± standard deviation (SD) or median [interquartile range] for continuous variables and percentages for categorical variables. Standardised mean differences (SMD) < 0.1 indicate adequate balance. Before matching, JAK inhibitor initiators in this stratum differed from TNFi initiators most markedly in prior non-TNF biologic DMARD exposure (abatacept 11.9% versus 3.4%, SMD 0.325; tocilizumab 7.9% versus 1.9%, SMD 0.279; rituximab 3.3% versus 1.0%, SMD 0.161) and in corticosteroid and csDMARD exposure (methylprednisolone SMD 0.226; leflunomide 0.203; hydroxychloroquine 0.144; dexamethasone 0.115; prednisone 0.114); TNFi initiators carried slightly more hypertension (48.2% versus 44.5%) and nicotine dependence (13.9% versus 11.9%). After matching, every SMD was below 0.06. The largest residual differences were for prior tocilizumab exposure (6.5% versus 5.2%; SMD 0.057; *P* = 0.002), abatacept (9.8% versus 8.5%; SMD 0.045; *P* = 0.014), baseline C-reactive protein (14.1 ± 27.5 versus 15.2 ± 30.5 mg/L; SMD 0.035) and rituximab (2.9% versus 2.3%; SMD 0.034); all cardiovascular, csDMARD and corticosteroid covariates were balanced to ≤ 0.020. As in every other stratum, residual prior biologic exposure remained more frequent in the JAK inhibitor arm; baseline C-reactive protein, by contrast, was marginally higher in the TNFi arm, so the two residuals act in opposing directions in this stratum. As expected for a low-risk stratum, baseline cardiovascular comorbidity was uncommon in both arms (ischaemic heart disease 1.2%, heart failure 2.1%, atrial fibrillation 3.0%). Interquartile range is reported as the width of the interquartile range. ASCVD = atherosclerotic cardiovascular disease; JAKi = Janus kinase inhibitors; TNFi = tumour necrosis factor inhibitors; SD = standard deviation; SMD = standardised mean difference.

**Supplementary Table S15. Baseline characteristics in the ASCVD-free, two–risk-factor JAK inhibitor versus TNF inhibitor propensity score–matched cohort**

| **Characteristic** | **Before: JAKi (n = 4,767)** | **Before: TNFi (n = 13,047)** | **SMD** | **After: JAKi (n = 4,550)** | **After: TNFi (n = 4,550)** | **SMD** |
| --- | --- | --- | --- | --- | --- | --- |
| **Demographics** |  |  |  |  |  |  |
| Age, current, years (mean ± SD) | 67.3 ± 8.93 | 68.3 ± 9.38 | 0.1064 | 67.4 ± 8.93 | 67.2 ± 9.21 | 0.0174 |
| Age at index, years (mean ± SD) | 62.2 ± 8.89 | 61.8 ± 9.29 | 0.0535 | 62.3 ± 8.90 | 62.5 ± 9.23 | 0.0210 |
| Female, % | 80.85 | 77.08 | 0.0927 | 80.57 | 81.01 | 0.0112 |
| Male, % | 19.15 | 22.91 | 0.0923 | 19.43 | 18.99 | 0.0112 |
| Unknown gender, % | 0 | 0.08 | 0.0392 | 0 | 0 | — |
| White, % | 66.23 | 70.21 | 0.0856 | 66.46 | 66.26 | 0.0042 |
| Black or African American, % | 13.89 | 14.92 | 0.0295 | 14.09 | 14.66 | 0.0163 |
| Asian, % | 4.51 | 2.85 | 0.0882 | 4.40 | 4.09 | 0.0153 |
| American Indian or Alaska Native, % | 0.65 | 0.74 | 0.0112 | 0.66 | 0.75 | 0.0105 |
| Native Hawaiian or Other Pacific Islander, % | 0.27 | 0.35 | 0.0130 | 0.26 | 0.24 | 0.0044 |
| Other race, % | 2.50 | 3.19 | 0.0417 | 2.44 | 2.44 | < 0.0001 |
| Unknown race, % | 11.96 | 7.74 | 0.1418 | 11.69 | 11.56 | 0.0041 |
| Hispanic or Latino, % | 6.46 | 7.41 | 0.0374 | 6.40 | 6.40 | < 0.0001 |
| Not Hispanic or Latino, % | 67.32 | 68.34 | 0.0218 | 67.34 | 67.74 | 0.0084 |
| Unknown ethnicity, % | 26.22 | 24.25 | 0.0454 | 26.26 | 25.87 | 0.0090 |
| **Cardiovascular risk factors and comorbidities** |  |  |  |  |  |  |
| Hypertension, % | 79.40 | 78.72 | 0.0166 | 79.63 | 79.82 | 0.0049 |
| Dyslipidemia, % | 67.88 | 64.07 | 0.0806 | 67.65 | 67.67 | 0.0005 |
| Overweight and obesity, % | 27.71 | 25.44 | 0.0515 | 27.36 | 27.47 | 0.0025 |
| Type 2 diabetes, % | 17.54 | 18.38 | 0.0219 | 17.58 | 17.52 | 0.0017 |
| Nicotine dependence, % | 15.48 | 15.77 | 0.0080 | 15.41 | 15.47 | 0.0018 |
| Tobacco use, % | 5.20 | 4.86 | 0.0157 | 5.01 | 5.60 | 0.0265 |
| Ischaemic heart disease, % | 2.37 | 1.73 | 0.0450 | 2.33 | 2.35 | 0.0015 |
| Cerebral infarction, % | 0.50 | 0.41 | 0.0145 | 0.51 | 0.48 | 0.0031 |
| Heart failure, % | 3.67 | 2.95 | 0.0403 | 3.71 | 3.63 | 0.0047 |
| Atrial fibrillation, % | 3.84 | 4.38 | 0.0275 | 3.98 | 3.87 | 0.0057 |
| Chronic kidney disease, % | 7.15 | 6.32 | 0.0331 | 7.23 | 7.43 | 0.0076 |
| Acute kidney injury, % | 5.08 | 3.55 | 0.0753 | 5.12 | 5.05 | 0.0030 |
| **Other autoimmune and inflammatory diagnoses** |  |  |  |  |  |  |
| Systemic lupus erythematosus, % | 3.99 | 2.91 | 0.0588 | 3.85 | 3.60 | 0.0128 |
| Systemic sclerosis, % | 0.36 | 0.38 | 0.0044 | 0.37 | 0.29 | 0.0153 |
| Dermatomyositis, % | 0.63 | 0.34 | 0.0421 | 0.57 | 0.46 | 0.0153 |
| Antiphospholipid syndrome, % | 0.25 | 0.26 | 0.0018 | 0.24 | 0.22 | 0.0046 |
| Arthropathic psoriasis, % | 0.42 | 0.28 | 0.0230 | 0.42 | 0.33 | 0.0144 |
| Ankylosing spondylitis, % | 0.29 | 0.13 | 0.0355 | 0.29 | 0.26 | 0.0042 |
| Crohn's disease, % | 0.21 | 0.15 | 0.0152 | 0.22 | 0.22 | < 0.0001 |
| Ulcerative colitis, % | 0.21 | 0.08 | 0.0352 | 0.22 | 0.22 | < 0.0001 |
| **csDMARDs and corticosteroids** |  |  |  |  |  |  |
| Methotrexate, % | 49.82 | 49.65 | 0.0034 | 49.32 | 50.64 | 0.0264 |
| Hydroxychloroquine, % | 33.02 | 29.69 | 0.0717 | 32.57 | 32.95 | 0.0080 |
| Leflunomide, % | 21.75 | 15.08 | 0.1727 | 20.84 | 19.98 | 0.0213 |
| Sulfasalazine, % | 15.54 | 12.42 | 0.0901 | 15.34 | 15.30 | 0.0012 |
| Prednisone, % | 59.85 | 53.52 | 0.1279 | 59.01 | 60.86 | 0.0377 |
| Methylprednisolone, % | 42.00 | 32.53 | 0.1968 | 40.59 | 40.95 | 0.0072 |
| Dexamethasone, % | 27.63 | 21.67 | 0.1386 | 27.25 | 27.98 | 0.0162 |
| **Prior non-TNF biologic DMARDs** |  |  |  |  |  |  |
| Abatacept, % | 11.85 | 3.31 | 0.3270 | 9.69 | 8.81 | 0.0303 |
| Tocilizumab, % | 8.54 | 1.96 | 0.2981 | 6.42 | 5.32 | 0.0468 |
| Rituximab, % | 3.67 | 0.82 | 0.1933 | 2.64 | 2.00 | 0.0424 |
| **Laboratory parameters** |  |  |  |  |  |  |
| C-reactive protein, mg/L (mean ± SD) | 15.8 ± 30.6 | 15.4 ± 30.0 | 0.0134 | 16.0 ± 31.1 | 15.8 ± 30.7 | 0.0078 |
| C-reactive protein, mg/L (median [IQR]) | 6.0 [13.0] | 6.3 [12.4] | — | 6.0 [12.9] | 6.2 [12.6] | — |
| C-reactive protein recorded, % | 61.72 | 54.08 | 0.1551 | 60.57 | 60.90 | 0.0068 |
| Erythrocyte sedimentation rate, mm/h (mean ± SD) | 27.3 ± 24.3 | 26.8 ± 24.2 | 0.0209 | 27.4 ± 24.3 | 26.9 ± 24.2 | 0.0215 |
| Erythrocyte sedimentation rate, mm/h (median [IQR]) | 20 [29] | 19 [28] | — | 20 [28] | 19 [28] | — |
| Erythrocyte sedimentation rate recorded, % | 59.89 | 55.98 | 0.0794 | 59.30 | 59.69 | 0.0081 |

Baseline characteristics of patients without established atherosclerotic cardiovascular disease and with two baseline cardiovascular risk factors, initiating a JAK inhibitor versus a tumour necrosis factor inhibitor (TNFi), before and after 1:1 propensity score matching. The propensity score model included demographics, cardiovascular risk factors and comorbidities, other autoimmune and inflammatory diagnoses, conventional synthetic DMARDs, corticosteroids, prior non-TNF biologic DMARDs, and baseline inflammatory markers. Values are mean ± standard deviation (SD) or median [interquartile range] for continuous variables and percentages for categorical variables. Standardised mean differences (SMD) < 0.1 indicate adequate balance. Before matching, JAK inhibitor initiators in this stratum differed from TNFi initiators most markedly in prior non-TNF biologic DMARD exposure (abatacept 11.9% versus 3.3%, SMD 0.327; tocilizumab 8.5% versus 2.0%, SMD 0.298; rituximab 3.7% versus 0.8%, SMD 0.193) and in corticosteroid and csDMARD exposure (methylprednisolone SMD 0.197; leflunomide 0.173; dexamethasone 0.139; prednisone 0.128); the cardiovascular risk factors defining the stratum were closely comparable between groups (hypertension SMD 0.017; type 2 diabetes 0.022; nicotine dependence 0.008), as expected given the stratification. After matching, all demographic, cardiovascular, csDMARD and corticosteroid SMDs were ≤ 0.038. The largest residual differences were for prior tocilizumab exposure (6.4% versus 5.3%; SMD 0.047; *P* = 0.026), rituximab (2.6% versus 2.0%; SMD 0.042; *P* = 0.043), prednisone (0.038) and abatacept (0.030), with prior biologic exposure remaining more frequent in the JAK inhibitor arm in each case. Baseline C-reactive protein (16.0 ± 31.1 versus 15.8 ± 30.7 mg/L; SMD 0.008) and erythrocyte sedimentation rate (SMD 0.022) were well balanced. By construction this stratum carries a higher cardiovascular risk factor burden than the one–risk-factor stratum (hypertension 79.6%, dyslipidemia 67.7%, type 2 diabetes 17.6%) while remaining free of established atherosclerotic disease. Interquartile range is reported as the width of the interquartile range. ASCVD = atherosclerotic cardiovascular disease; JAKi = Janus kinase inhibitors; TNFi = tumour necrosis factor inhibitors; SD = standard deviation; SMD = standardised mean difference.

**Supplementary Table S16. Baseline characteristics in the ASCVD-free, ≥3 risk factor JAK inhibitor versus TNF inhibitor propensity score–matched cohort**

| **Characteristic** | **Before: JAKi (n = 2,649)** | **Before: TNFi (n = 7,352)** | **SMD** | **After: JAKi (n = 2,508)** | **After: TNFi (n = 2,508)** | **SMD** |
| --- | --- | --- | --- | --- | --- | --- |
| **Demographics** |  |  |  |  |  |  |
| Age, current, years (mean ± SD) | 66.8 ± 8.72 | 67.2 ± 9.02 | 0.0440 | 66.8 ± 8.71 | 66.9 ± 9.05 | 0.0173 |
| Age at index, years (mean ± SD) | 61.8 ± 8.65 | 60.9 ± 8.94 | 0.1019 | 61.8 ± 8.64 | 62.2 ± 9.05 | 0.0458 |
| Female, % | 79.46 | 74.67 | 0.1141 | 79.03 | 79.59 | 0.0138 |
| Male, % | 20.50 | 25.33 | 0.1151 | 20.93 | 20.41 | 0.0128 |
| Unknown gender, % | 0.38 | 0 | 0.0871 | 0.40 | 0 | 0.0895 ‡ |
| White, % | 62.21 | 63.55 | 0.0276 | 62.52 | 62.04 | 0.0099 |
| Black or African American, % | 20.39 | 21.52 | 0.0278 | 20.53 | 21.65 | 0.0274 |
| Asian, % | 3.89 | 2.79 | 0.0613 | 3.67 | 3.35 | 0.0173 |
| American Indian or Alaska Native, % | 0.87 | 0.95 | 0.0088 | 0.92 | 0.80 | 0.0130 |
| Native Hawaiian or Other Pacific Islander, % | 0.57 | 0.46 | 0.0145 | 0.52 | 0.64 | 0.0158 |
| Other race, % | 2.72 | 3.75 | 0.0586 | 2.59 | 2.63 | 0.0025 |
| Unknown race, % | 9.36 | 6.98 | 0.0871 | 9.25 | 8.89 | 0.0125 |
| Hispanic or Latino, % | 9.29 | 9.34 | 0.0020 | 8.85 | 8.85 | < 0.0001 |
| Not Hispanic or Latino, % | 67.04 | 68.39 | 0.0288 | 67.46 | 66.99 | 0.0102 |
| Unknown ethnicity, % | 23.67 | 22.27 | 0.0334 | 23.68 | 24.16 | 0.0112 |
| **Cardiovascular risk factors and comorbidities** |  |  |  |  |  |  |
| Hypertension, % | 94.94 | 92.34 | 0.1067 | 94.86 | 95.77 | 0.0434 |
| Dyslipidemia, % | 90.26 | 86.33 | 0.1225 | 90.31 | 90.03 | 0.0094 |
| Type 2 diabetes, % | 73.46 | 70.70 | 0.0616 | 73.56 | 73.92 | 0.0082 |
| Overweight and obesity, % | 43.53 | 39.45 | 0.0829 | 43.10 | 43.82 | 0.0145 |
| Nicotine dependence, % | 32.16 | 32.82 | 0.0140 | 31.94 | 32.14 | 0.0043 |
| Tobacco use, % | 11.55 | 11.59 | 0.0012 | 11.56 | 11.04 | 0.0164 |
| Ischaemic heart disease, % | 3.51 | 3.22 | 0.0159 | 3.31 | 3.23 | 0.0045 |
| Cerebral infarction, % | 0.98 | 0.86 | 0.0131 | 1.00 | 1.28 | 0.0263 |
| Heart failure, % | 6.76 | 5.37 | 0.0580 | 6.86 | 7.38 | 0.0202 |
| Atrial fibrillation, % | 5.17 | 4.69 | 0.0221 | 5.30 | 5.58 | 0.0123 |
| Chronic kidney disease, % | 13.89 | 11.36 | 0.0764 | 13.64 | 14.07 | 0.0127 |
| Acute kidney injury, % | 9.85 | 7.07 | 0.1000 | 9.65 | 10.45 | 0.0265 |
| **Other autoimmune and inflammatory diagnoses** |  |  |  |  |  |  |
| Systemic lupus erythematosus, % | 4.83 | 3.24 | 0.0811 | 4.63 | 4.63 | < 0.0001 |
| Systemic sclerosis, % | 0.45 | 0.35 | 0.0157 | 0.44 | 0.40 | 0.0062 |
| Dermatomyositis, % | 0.91 | 0.33 | 0.0741 | 0.72 | 0.68 | 0.0048 |
| Antiphospholipid syndrome, % | 0.38 | 0.35 | 0.0040 | 0.40 | 0.40 | < 0.0001 |
| Arthropathic psoriasis, % | 0.45 | 0.30 | 0.0251 | 0.40 | 0.44 | 0.0062 |
| Ankylosing spondylitis, % | 0.53 | 0.16 | 0.0622 | 0.40 | 0.44 | 0.0062 |
| Crohn's disease, % | 0.38 | 0.20 | 0.0322 | 0.40 | 0.40 | < 0.0001 |
| Ulcerative colitis, % | 0.38 | 0.14 | 0.0477 | 0.40 | 0.40 | < 0.0001 |
| **csDMARDs and corticosteroids** |  |  |  |  |  |  |
| Methotrexate, % | 50.62 | 52.31 | 0.0338 | 49.92 | 48.60 | 0.0263 |
| Hydroxychloroquine, % | 36.09 | 30.18 | 0.1257 | 35.17 | 32.78 | 0.0505 |
| Leflunomide, % | 24.76 | 15.79 | 0.2246 | 23.48 | 23.48 | < 0.0001 |
| Sulfasalazine, % | 17.93 | 13.07 | 0.1346 | 17.11 | 17.34 | 0.0063 |
| Prednisone, % | 64.85 | 57.86 | 0.1440 | 63.84 | 65.03 | 0.0250 |
| Methylprednisolone, % | 48.02 | 38.14 | 0.2005 | 46.37 | 48.72 | 0.0471 |
| Dexamethasone, % | 34.01 | 26.31 | 0.1685 | 33.17 | 34.37 | 0.0253 |
| **Prior non-TNF biologic DMARDs** |  |  |  |  |  |  |
| Abatacept, % | 12.46 | 3.36 | 0.3420 | 9.77 | 8.45 | 0.0457 |
| Tocilizumab, % | 9.29 | 1.81 | 0.3311 | 6.62 | 5.18 | 0.0609 |
| Rituximab, % | 3.28 | 0.97 | 0.1613 | 2.39 | 2.27 | 0.0079 |
| **Laboratory parameters** |  |  |  |  |  |  |
| C-reactive protein, mg/L (mean ± SD) | 17.7 ± 34.3 | 17.0 ± 31.3 | 0.0205 | 17.9 ± 35.0 | 17.3 ± 31.5 | 0.0198 |
| C-reactive protein, mg/L (median [IQR]) | 7.0 [13.8] | 7.7 [13.7] | — | 7.0 [13.6] | 7.2 [14.0] | — |
| C-reactive protein recorded, % | 66.29 | 59.37 | 0.1435 | 65.27 | 66.27 | 0.0210 |
| Erythrocyte sedimentation rate, mm/h (mean ± SD) | 30.4 ± 25.2 | 30.3 ± 24.6 | 0.0035 | 30.3 ± 25.2 | 31.5 ± 25.4 | 0.0493 |
| Erythrocyte sedimentation rate, mm/h (median [IQR]) | 24 [32] | 24 [30] | — | 24 [31] | 24 [32] | — |
| Erythrocyte sedimentation rate recorded, % | 63.50 | 60.92 | 0.0531 | 62.72 | 63.12 | 0.0083 |

‡ Standardised mean difference derived from a cell in which one arm contained fewer than the minimum reportable patient count; the platform reports such counts as 10 or 0, so this value reflects count obfuscation rather than a measurable imbalance.

Baseline characteristics of patients without established atherosclerotic cardiovascular disease and with three or more baseline cardiovascular risk factors, initiating a JAK inhibitor versus a tumour necrosis factor inhibitor (TNFi), before and after 1:1 propensity score matching. The propensity score model included demographics, cardiovascular risk factors and comorbidities, other autoimmune and inflammatory diagnoses, conventional synthetic DMARDs, corticosteroids, prior non-TNF biologic DMARDs, and baseline inflammatory markers. Values are mean ± standard deviation (SD) or median [interquartile range] for continuous variables and percentages for categorical variables. Standardised mean differences (SMD) < 0.1 indicate adequate balance. This is the smallest of the three cardiovascular risk strata (2,508 pairs). Before matching, JAK inhibitor initiators differed from TNFi initiators most markedly in prior non-TNF biologic DMARD exposure (abatacept 12.5% versus 3.4%, SMD 0.342; tocilizumab 9.3% versus 1.8%, SMD 0.331; rituximab 3.3% versus 1.0%, SMD 0.161) and in corticosteroid and csDMARD exposure (leflunomide SMD 0.225; methylprednisolone 0.201; dexamethasone 0.169; prednisone 0.144); the cardiovascular risk factors defining the stratum differed only modestly (hypertension SMD 0.107; dyslipidemia 0.123; type 2 diabetes 0.062; nicotine dependence 0.014). Excluding covariates affected by low-count obfuscation, after matching the largest residual differences were for prior tocilizumab exposure (6.6% versus 5.2%; SMD 0.061; *P* = 0.031), hydroxychloroquine (0.051), erythrocyte sedimentation rate (30.3 ± 25.2 versus 31.5 ± 25.4 mm/h; SMD 0.049), methylprednisolone (0.047), age at index (0.046) and abatacept (0.046); all remaining covariates were balanced to ≤ 0.044. Baseline C-reactive protein was well balanced (17.9 ± 35.0 versus 17.3 ± 31.5 mg/L; SMD 0.020). Despite exclusion of established atherosclerotic disease, this stratum carries a heavy risk factor burden (hypertension 94.9%, dyslipidemia 90.3%, type 2 diabetes 73.6%, overweight or obesity 43.1%). Interquartile range is reported as the width of the interquartile range. ASCVD = atherosclerotic cardiovascular disease; JAKi = Janus kinase inhibitors; TNFi = tumour necrosis factor inhibitors; SD = standard deviation; SMD = standardised mean difference.

**Supplementary Table S17. Duration of available electronic health record observation after index**

| **Cohort** | **n** | **Mean (days)** | **SD** | **Median (days)** | **IQR (days)** |
| --- | --- | --- | --- | --- | --- |
| JAKi | 18,757 | 1,171.528 | 652.542 | 1,320 | 1,259 |
| TNFi | 18,757 | 1,080.026 | 651.737 | 1,111 | 1,360 |

This metric measures how long a patient's records persist in the network after the index date — that is, availability of observation — and is not equivalent to person-time at risk, which cannot be derived from the platform's exports. It should be reported as median duration of available EHR observation after index, and never as median follow-up.

Observation duration is longer in the JAKi arm by approximately 91 days at the mean and 209 days at the median. This direction is counterintuitive for the more recently introduced class and is consistent with more intensive monitoring. Could not be exported for the agent-specific, sex, cardiovascular-risk or index-era cohorts before network access ended; reported for the main matched cohort only.

**Supplementary Table S18. Agent × index-year strata**

Each agent-by-era cell was matched independently to contemporaneous TNFi initiators.

| **Agent** | **Index year** | **n per group** | **Outcome** | **Events JAKi** | **Events TNFi** | **5-yr surv. JAKi (%)** | **5-yr surv. TNFi (%)** | **Hazard ratio** | **95% CI** | **Log-rank P** | **PH P** |
| --- | --- | --- | --- | --- | --- | --- | --- | --- | --- | --- | --- |
| Tofacitinib | Pre-2021 | 7,777 | MACE | 694 | 701 | 90.128 | 90.124 | 0.998 | (0.898–1.108) | 0.9680 | 0.7681 |
|  |  |  | All-cause mortality | 645 | 581 | 90.926 | 91.882 | 1.123 | (1.004–1.256) | 0.0428 | 0.9902 |
|  |  |  | Heart failure hospitalisation | 994 | 923 | 86.019 | 87.111 | 1.091 | (0.998–1.194) | 0.0560 | 0.9318 |
|  |  |  | Venous thromboembolism | 576 | 520 | 91.851 | 92.680 | 1.120 | (0.995–1.261) | 0.0601 | 0.5466 |
|  |  |  | 3-component MACE | 1,203 | 1,158 | 83.227 | 83.979 | 1.049 | (0.967–1.137) | 0.2472 | 0.6169 |
| Tofacitinib | Post-2021 | 4,802 | MACE | 260 | 280 | 89.392 | 89.116 | 0.939 | (0.793–1.112) | 0.4638 | 0.1923 |
|  |  |  | All-cause mortality | 212 | 190 | 91.238 | 91.937 | 1.136 | (0.934–1.382) | 0.2013 | 0.3897 |
|  |  |  | Heart failure hospitalisation | 434 | 453 | 85.123 | 84.790 | 0.984 | (0.862–1.122) | 0.8060 | 0.2931 |
|  |  |  | Venous thromboembolism | 202 | 220 | 92.702 | 92.142 | 0.937 | (0.774–1.135) | 0.5067 | 0.9859 |
|  |  |  | 3-component MACE | 426 | 426 | 82.946 | 83.388 | 1.012 | (0.885–1.157) | 0.8626 | 0.2957 |
| Baricitinib | Pre-2021 | 469 | MACE | 46 | 39 | 88.746 | 90.493 | 1.181 | (0.771–1.809) | 0.4453 | 0.4622 |
|  |  |  | All-cause mortality | 59 | 26 | 86.051 | 93.644 | 2.271 | (1.432–3.602) | 0.0003 | 0.6607 |
|  |  |  | Heart failure hospitalisation | 58 | 62 | 86.048 | 84.796 | 0.917 | (0.641–1.312) | 0.6349 | 0.9890 |
|  |  |  | Venous thromboembolism | 29 | 26 | 92.878 | 93.837 | 1.105 | (0.651–1.876) | 0.7123 | 0.0978 |
|  |  |  | 3-component MACE | 95 | 62 | 77.762 | 85.003 | 1.540 | (1.119–2.121) | 0.0077 | 0.7234 |
| Baricitinib | Post-2021 | 1,205 | MACE | 91 | 120 | 84.856 | 83.554 | 0.885 | (0.674–1.163) | 0.3805 | 0.6419 |
|  |  |  | All-cause mortality | 75 | 77 | 86.639 | 86.469 | 1.142 | (0.831–1.569) | 0.4139 | 0.1371 |
|  |  |  | Heart failure hospitalisation | 172 | 217 | 75.300 | 72.718 | 0.920 | (0.753–1.123) | 0.4111 | 0.9197 |
|  |  |  | Venous thromboembolism | 82 | 83 | 86.006 | 88.114 | 1.172 | (0.864–1.590) | 0.3081 | 0.5831 |
|  |  |  | 3-component MACE | 146 | 179 | 77.401 | 74.560 | 0.956 | (0.768–1.190) | 0.6865 | 0.2561 |
| Upadacitinib | Pre-2021 | 992 | MACE | 88 | 86 | 89.874 | 90.453 | 1.055 | (0.784–1.420) | 0.7254 | 0.5388 |
|  |  |  | All-cause mortality | 81 | 77 | 90.764 | 91.439 | 1.088 | (0.796–1.486) | 0.5960 | 0.7953 |
|  |  |  | Heart failure hospitalisation | 122 | 123 | 86.158 | 86.602 | 1.018 | (0.792–1.308) | 0.8892 | 0.3883 |
|  |  |  | Venous thromboembolism | 67 | 68 | 92.328 | 92.454 | 1.011 | (0.721–1.417) | 0.9494 | 0.6910 |
|  |  |  | 3-component MACE | 150 | 145 | 83.113 | 84.176 | 1.066 | (0.848–1.339) | 0.5853 | 0.5932 |
| Upadacitinib | Post-2021 | 6,482 | MACE | 259 | 312 | 90.457 | 89.308 | 0.825 | (0.700–0.973) | 0.0221 | 0.6986 |
|  |  |  | All-cause mortality | 174 | 212 | 92.859 | 90.875 | 0.815 | (0.667–0.996) | 0.0451 | 0.8925 |
|  |  |  | Heart failure hospitalisation | 487 | 499 | 86.070 | 85.561 | 0.981 | (0.866–1.112) | 0.7640 | 0.7865 |
|  |  |  | Venous thromboembolism | 223 | 241 | 92.800 | 92.688 | 0.926 | (0.772–1.112) | 0.4109 | 0.0126 * |
|  |  |  | 3-component MACE | 395 | 472 | 85.383 | 83.779 | 0.831 | (0.727–0.950) | 0.0065 | 0.2949 |

* Proportional hazards assumption violated (*P* < 0.05).

**Supplementary Table S19. E-values for nominally significant associations**

E-values computed as E = HR + √(HR × (HR − 1)); protective hazard ratios are inverted before computation. Confidence-limit E-values use the bound closest to the null.

| **Association** | **Hazard ratio** | **95% CI** | **E-value** | **E-value at CI limit** | **Interpretation** |
| --- | --- | --- | --- | --- | --- |
| All-cause mortality, main cohort | 1.158 | (1.058–1.268) | 1.586 | 1.306 | Primary finding |
| All-cause mortality, female | 1.123 | (1.013–1.244) | 1.495 | 1.128 | Subgroup |
| Baricitinib venous thromboembolism | 1.364 | (1.028–1.809) | 2.069 | 1.198 | Hypothesis-generating |
| Tofacitinib pre-2021 mortality | 1.123 | (1.004–1.256) | 1.495 | 1.067 | Era-confounded |
| Baricitinib pre-2021 mortality | 2.271 | (1.432–3.602) | 3.970 | 2.219 | 85 events, n = 469 |
| Baricitinib pre-2021 3-component MACE | 1.540 | (1.119–2.121) | 2.452 | 1.484 | 157 events, n = 469 |
| Upadacitinib post-2021 MACE | 0.825 | (0.700–0.973) | 1.719 | 1.197 | Protective; era-confounded |
| Upadacitinib post-2021 mortality | 0.815 | (0.667–0.996) | 1.755 | 1.068 | Protective; era-confounded |
| Upadacitinib post-2021 3-component MACE | 0.831 | (0.727–0.950) | 1.698 | 1.288 | Protective; era-confounded |

An E-value of 1.586 for the primary mortality association means that an unmeasured confounder would need to be associated with both JAK inhibitor initiation and death by a risk ratio of approximately 1.59 each, over and above every measured covariate, to explain the association away.

**Supplementary Table S20. Hazard ratios by propensity score model specification, main matched cohort**

| **Outcome** | **Model 1: + individual csDMARDs, oral corticosteroids, ESR (submitted version; 17,607 pairs)** | **Model 2: + prior non-TNF biologics, C-reactive protein; refreshed extraction (18,757 pairs)** |
| --- | --- | --- |
| MACE | 1.024 (0.941–1.115), p = 0.5755 | 1.002 (0.923–1.088), p = 0.9579 |
| All-cause mortality | 1.205 (1.097–1.324), p = 0.0001 | 1.158 (1.058–1.268), p = 0.0014 |
| Heart failure hospitalisation | 1.111 (1.040–1.187), p = 0.0018 | 1.019 (0.955–1.088), p = 0.5653 |
| Venous thromboembolism | 1.069 (0.973–1.175), p = 0.1659 | 1.054 (0.961–1.156), p = 0.2631 |
| 3-component MACE | 1.084 (1.014–1.158), p = 0.0170 | 1.058 (0.992–1.127), p = 0.0844 |
| Cataract (negative control) | Not analysed | 0.934 (0.867–1.007), p = 0.0739 |

Hazard ratios (95% CI) for JAKi versus TNFi, five-year follow-up from the 90-day landmark. Model 1 adds individual conventional synthetic DMARDs (methotrexate, hydroxychloroquine, leflunomide, sulfasalazine), oral corticosteroids (prednisone, methylprednisolone, dexamethasone) and baseline erythrocyte sedimentation rate. Model 2 adds prior non-TNF biologic exposure (abatacept, tocilizumab, rituximab) and C-reactive protein, and was estimated on a refreshed network extraction; the effects of the expanded model and the refreshed extraction therefore cannot be separated. The specification reported in the earlier preprint [24] is not tabulated here: it differed from these models in cohort derivation, in the handling of prevalent events at baseline, and in the risk metric reported, and is therefore not directly comparable.

**Supplementary Table S21. Pre-matching eligible pools and extraction dates by cohort**

| **Cohort** | **Table** | **Extraction** | **JAKi pool** | **TNFi pool** |
| --- | --- | --- | --- | --- |
| **Main matched cohort** | **Table 1** | **23 Jul 2026** | **19,485** | **55,266** |
| **Tofacitinib** | **S1** | **25 Jul 2026** | **13,125** | **56,175** |
| **Baricitinib** | **S2** | **25 Jul 2026** | **1,729** | **57,077** |
| **Upadacitinib** | **S3** | **25 Jul 2026** | **8,192** | **57,077** |
| **Tofacitinib, pre-2021** | **S4** | **25 Jul 2026** | **8,160** | **31,643** |
| **Tofacitinib, post-2021** | **S5** | **25 Jul 2026** | **4,826** | **24,123** |
| **Baricitinib, pre-2021** | **S6** | **25 Jul 2026** | **481** | **31,643** |
| **Baricitinib, post-2021** | **S7** | **25 Jul 2026** | **1,231** | **24,123** |
| **Upadacitinib, pre-2021** | **S8** | **25 Jul 2026** | **1,060** | **31,643** |
| **Upadacitinib, post-2021** | **S9** | **25 Jul 2026** | **7,053** | **24,123** |
| **All JAKi, pre-2021** | **S10** | **25 Jul 2026** | **9,075** | **31,643** |
| **All JAKi, post-2021** | **S11** | **25 Jul 2026** | **10,766** | **24,123** |
| **Female** | **S12** | **25 Jul 2026** | **16,152** | **43,018** |
| **Male** | **S13** | **25 Jul 2026** | **4,133** | **14,056** |
| **ASCVD-free, 1 risk factor** | **S14** | **25 Jul 2026** | **6,102** | **17,115** |
| **ASCVD-free, 2 risk factors** | **S15** | **25 Jul 2026** | **4,767** | **13,047** |
| **ASCVD-free, ≥3 risk factors** | **S16** | **25 Jul 2026** | **2,649** | **7,352** |

Pre-matching eligible pools only. Each cohort was constructed as an independent query against the network rather than by subsetting a single extraction; because contributing organisations upload continuously, each denominator reflects the network state when that query ran, including between queries run on the same day. Individual strata are subsets of the eligible population and are smaller than the pooled figures by construction, and the ASCVD-free risk strata exclude patients with established atherosclerotic cardiovascular disease and so are not exhaustive. Wherever a denominator is directly comparable with a main-cohort figure — the full eligible TNFi pool, or an exhaustive partition of either pool — it exceeds the main-cohort value and is never smaller: the TNFi pool is 55,266 in the main cohort against 56,175, 57,077 and 57,077 in the three agent cohorts, 57,074 summed across the sex strata and 55,766 summed across the index-era strata; the JAKi pool is 19,485 against 20,285 summed across the sex strata and 19,841 across the index-era strata. Matched cohort sizes are unaffected and are reported in Figure 1, Table 1 and Supplementary Tables S1–S16.
